# The SGLT2 inhibitor dapagliflozin in heart failure with preserved ejection fraction: a multicenter randomized trial

**DOI:** 10.1038/s41591-021-01536-x

**Published:** 2021-10-28

**Authors:** Michael E. Nassif, Sheryl L. Windsor, Barry A. Borlaug, Dalane W. Kitzman, Sanjiv J. Shah, Fengming Tang, Yevgeniy Khariton, Ali O. Malik, Taiyeb Khumri, Guillermo Umpierrez, Sumant Lamba, Kavita Sharma, Sadiya S. Khan, Lokesh Chandra, Robert A. Gordon, John J. Ryan, Sunit-Preet Chaudhry, Susan M. Joseph, Chen H. Chow, Manreet K. Kanwar, Michael Pursley, Elias S. Siraj, Gregory D. Lewis, Barry S. Clemson, Michael Fong, Mikhail N. Kosiborod

**Affiliations:** 1 Saint Luke’s Mid America Heart Institute, Kansas City, MO USA; 2 Present Address: University of Missouri-Kansas City, Kansas City, MO USA; 3 Department of Cardiovascular Medicine, Mayo Clinic, Rochester, MN USA; 4 Department of Internal Medicine, Sections on Cardiovascular Medicine and Geriatrics, Wake Forest School of Medicine, Winston-Salem, NC USA; 5 Division of Cardiology, Department of Medicine and Bluhm Cardiovascular Institute, Northwestern University Feinberg School of Medicine, Chicago, IL USA; 6 Emory University, Atlanta, GA USA; 7 First Coast Cardiovascular Institute, Jacksonville, FL USA; 8 Johns Hopkins University School of Medicine, Baltimore, MD USA; 9 Chicago Medical Research, Hazel Crest, IL USA; 10 NorthShore University HealthSystem, Evanston, IL USA; 11 University of Utah, Salt Lake City, UT USA; 12 Ascension St. Vincent, Indianapolis, IN USA; 13 Division of Cardiovascular Medicine, University of Maryland School of Medicine, Baltimore, MD USA; 14 Stormont Vail Health, Topeka, KS USA; 15 Cardiovascular Institute, Allegheny Health Network, Pittsburgh, PA USA; 16 Heart Group of the Eastern Shore, Fairhope, AL USA; 17 Eastern Virginia Medical School, Norfolk, VA USA; 18 Cardiology Division, Massachusetts General Hospital, Boston, MA USA; 19 OSF HealthCare Cardiovascular Institute, Peoria, IL USA; 20 University of Southern California, Los Angeles, CA USA; 21 The George Institute for Global Health, Sydney, New South Wales Australia; 22 University of New South Wales, Sydney, New South Wales Australia

**Keywords:** Heart failure, Outcomes research

## Abstract

Patients with heart failure and preserved ejection fraction (HFpEF) have a high burden of symptoms and functional limitations, and have a poor quality of life. By targeting cardiometabolic abmormalities, sodium glucose cotransporter 2 (SGLT2) inhibitors may improve these impairments. In this multicenter, randomized trial of patients with HFpEF (NCT03030235), we evaluated whether the SGLT2 inhibitor dapagliflozin improves the primary endpoint of Kansas City Cardiomyopathy Questionnaire Clinical Summary Score (KCCQ-CS), a measure of heart failure-related health status, at 12 weeks after treatment initiation. Secondary endpoints included the 6-minute walk test (6MWT), KCCQ Overall Summary Score (KCCQ-OS), clinically meaningful changes in KCCQ-CS and -OS, and changes in weight, natriuretic peptides, glycated hemoglobin and systolic blood pressure. In total, 324 patients were randomized to dapagliflozin or placebo. Dapagliflozin improved KCCQ-CS (effect size, 5.8 points (95% confidence interval (CI) 2.3–9.2, *P* = 0.001), meeting the predefined primary endpoint, due to improvements in both KCCQ total symptom score (KCCQ-TS) (5.8 points (95% CI 2.0–9.6, *P* = 0.003)) and physical limitations scores (5.3 points (95% CI 0.7–10.0, *P* = 0.026)). Dapagliflozin also improved 6MWT (mean effect size of 20.1 m (95% CI 5.6–34.7, *P* = 0.007)), KCCQ-OS (4.5 points (95% CI 1.1–7.8, *P* = 0.009)), proportion of participants with 5-point or greater improvements in KCCQ-OS (odds ratio (OR) = 1.73 (95% CI 1.05–2.85, *P* = 0.03)) and reduced weight (mean effect size, 0.72 kg (95% CI 0.01–1.42, *P* = 0.046)). There were no significant differences in other secondary endpoints. Adverse events were similar between dapagliflozin and placebo (44 (27.2%) versus 38 (23.5%) patients, respectively). These results indicate that 12 weeks of dapagliflozin treatment significantly improved patient-reported symptoms, physical limitations and exercise function and was well tolerated in chronic HFpEF.

## Abstract (web summary)

In a multicenter, randomized trial, the SGLT2 inhibitor dapagliflozin improved the health status and exercise function of patients with heart failure with preserved ejection fraction (HFpEF), a condition for which effective treatments are lacking.

## Main

Heart failure (HF) with preserved ejection fraction (HFpEF) accounts for the majority of all HF in the community, and its prevalence is increasing as the population ages^1,2^. Patients with HFpEF experience an especially high burden of debilitating symptoms and physical limitations^3^. Improving health status (symptoms, functional status and quality of life) is therefore a key goal of HFpEF management, and is increasingly emphasized by practice guidelines and regulators^4–7^. To date, the wide range of pharmacotherapies tested have had minimal impact on these key outcomes, highlighting a critical unmet need.

Impaired health status in HFpEF is strongly linked to cardiometabolic abnormalities^8,9^. SGLT2 inhibitors target cardiometabolic conditions through a variety of mechanisms, and have been shown to reduce the risk of cardiovascular (CV) death or worsening HF, and to improve health status in patients with HF with reduced ejection fraction (HFrEF), regardless of diabetes status^10–13^. Although initial data from the outcome trials of empagliflozin and sotagliflozin (mixed SGLT1/2 inhibitor) suggest that they also reduce the risk of CV death and HF hospitalization in patients with HFpEF, the effects of SGLT2 inhibitors on patient-reported symptoms, physical limitations and objectively measured exercise function in this patient group remain uncertain^14,15^.

The PRESERVED-HF trial was designed to address this important knowledge gap by testing the hypothesis that treatment with the SGLT2 inhibitor dapagliflozin will improve symptoms, physical limitations and exercise function in patients with well-phenotyped HFpEF, both with and without type 2 diabetes (T2D).

## Results

### Patients

**Fig. 1:**
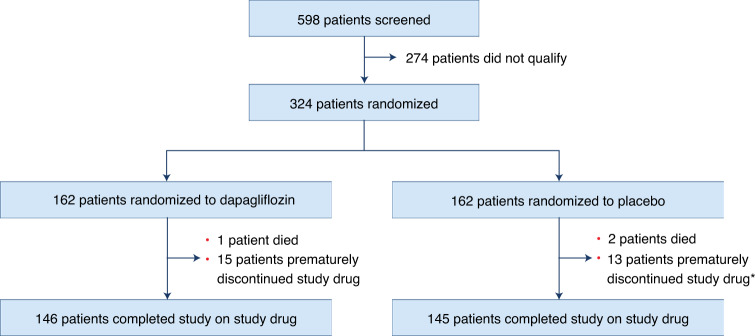
Trial flow chart. Breakdown of patients in study. *For two patients in the placebo group, no data were available on adherence with study medication at 12 weeks.

Between March 2017 and May 2021 a total of 598 patients were screened, of which 324 qualified and were randomized: 162 to dapagliflozin and 162 to placebo (Fig. 1). Baseline characteristics were generally well balanced between the two groups (Table 1). Overall, median age was 70.0 (63.0, 77.0) years, 57% of patients were women and 30% African American. The median duration of HF was 3.0 (1.0, 6.5) years and 56% had been hospitalized for HF at least once before study enrollment. Overall, 56% had T2D and 53% had AF; median body mass index (BMI) was 34.7 (interquartile range (IQR), 30.1–41.5). New York Heart Association (NYHA) class II symptoms were present in 57%, with class III/IV symptoms in 42%. Baseline pharmacotherapy included mineralocorticoid antagonists (MRA) in 36%, angiotensin-converting enzyme inhibitor (ACE-I), angiotensin II receptor blocker (ARB) or angiotensin receptor neprilysin inhibitor (ARNI) in 62% and loop diuretics in 88% of patients (with the remainder receiving thiazide diuretics, potassium-sparing diuretics or both). Those randomized to dapagliflozin were more likely to be on loop diuretics (93 versus 83%) and less likely to be on MRA (31 versus 42%) at baseline. Mean estimated glomerular filtration rate (eGFR) was 55 (41, 69) ml min^–1^ 1.73 m^–2^, median N-terminal pro B-type natriuretic peptide (NTproBNP) 671.0 (IQR = 355.0, 1297.0) pg ml^–1^ and median left ventricular ejection fraction (LVEF) was 60 (55, 65)%.

**Table 1:** Baseline characteristics

Baseline characteristics	Dapagliflozin (*n* = 162)	Placebo (*n* = 162)
**Demographics**		
Age, years	69 (64, 77)	71 (63, 78)
Women	92 (56.8%)	92 (56.8%)
White	108 (67.1%)	109 (69.0%)
African American	50 (31.1%)	47 (29.7%)
**Medical history**		
Duration of HF, years	3.0 (1.1, 6.5)	3.2 (1.0, 6.6)
Previous hospitalization for HF	98 (60.5%)	83 (51.2%)
Ejection fraction, %	60 (55, 65)	60 (54, 65)
Ischemic heart disease	32 (19.8%)	31 (19.1%)
T2D	90 (55.6%)	91 (56.2%)
AF	82 (50.6%)	89 (54.9%)
ICD	7 (4.3%)	9 (5.6%)
**Baseline HF/CV medications**		
ACE-I/ARB	98 (60.5%)	98 (60.5%)
ARNI	2 (1.2%)	3 (1.9%)
Beta-blockers	119 (73.5%)	116 (71.6%)
Hydralazine	25 (15.4%)	18 (11.1%)
Long-acting nitrates	34 (21.0%)	27 (16.7%)
MRA	50 (30.9%)	68 (42.0%)
Loop diuretics	151 (93.2%)	135 (83.3%)
Lipid-lowering agents	132 (81.5%)	127 (78.4%)
Anticoagulant agents	71 (43.8%)	84 (51.9%)
**Physical examination**		
BMI, median IQR	35.1 (30.4, 41.8)	34.6 (29.7, 40.4)
Heart rate	70 (61, 77)	68 (62, 75)
Systolic blood pressure	134 (120, 152)	132 (118, 148)
**Baseline laboratory studies**		
NTproBNP, pg ml^–1^, overall	641 (373, 1210)	710 (329, 1449)
NTproBNP, pg ml^–1^, AF	830 (555, 1711)	816 (481, 1687)
NTproBNP, pg ml^–1^, no AF	438 (269, 750)	485 (263, 1168)
BNP, pg ml^–1^, overall	137 (81, 222)	151 (90, 254)
BNP, pg ml^–1^, AF	169 (109,255)	151 (104, 258)
BNP, pg ml^–1^, no AF	107 (67, 179)	161 (77, 241)
eGFR, ml min^–1^	56 (42, 69)	54 (41, 69)
Hemoglobin A1c, %	6.0 (5.6, 7.3)	6.2 (5.6, 7.1)
Hemoglobin, g dl^–1^	12.7 (11.5, 13.9)	12.6 (11.6, 13.8)
**Functional measures**		
NYHA Class II	96 (59.3%)	90 (55.6%)
NYHA Class III/IV	65 (40.1%)	72 (44.4%)
KCCQ-OS	63.2 ± 20.4	62.3 ± 20.6
KCCQ-CS	63.4 ± 19.7	61.8 ± 20.3
6MWT (m), median (IQR)	244 (165, 329)	244 (154, 317)

Values are shown as absolute numbers (percentages) and median (IQR) or mean ± s.d.

ICD, internal cardiac defibrillator.

*Blood pressure and heart rate were measured from noninvasive cuff measumrents for patients in sinus rhythm and from manual pulse and blood pressure for patients in AF.

Premature, permanent treatment discontinuation for reasons other than death occurred in 15 dapagliflozin-treated and 13 placebo-treated patients, resulting in 146 and 145 patients in the dapagliflozin and placebo groups, respectively, completing the trial on study medication (Fig. 1). Safety follow-up was completed in all patients; no patients withdrew consent or were lost to follow-up, and the vital status was known for all participants.

### Primary endpoint

**Fig. 2:**
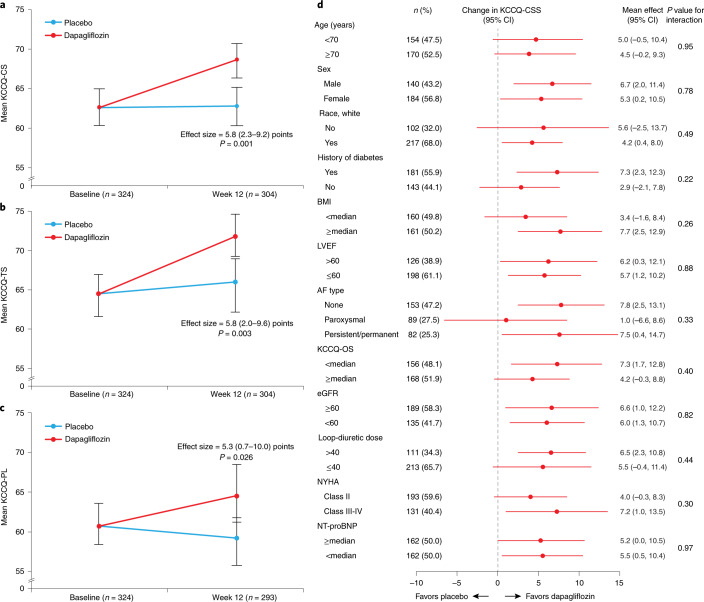
Effects of dapagliflozin on the primary endpoint and its components. **a**–**d**, Effects of dapagliflozin on the primary endpoint and its components. Effects of dapagliflozin versus placebo at 12 weeks on KCCQ-CS (**a**), KCCQ-TS (**b**), KCCQ-physical limitations score (KCCQ-PL) (**c**) and KCCQ-CS by subgroup (**d**). Units for loop diuretic dose (**d**), mg furosemide equivalents. Data are presented as mean values with 95% CI. **a**–**c**, An *F*-test was used in the data analysis. All *P* values are two-sided, with no adjustments made for multiple comparisons.

The primary endpoint was available in 304 (93.8%) patients at 12 weeks (152 (93.8%) in the dapagliflozin group and 152 (93.8%) in the placebo group). Dapagliflozin improved KCCQ-CS at 12 weeks (effect size, 5.8 points (95% CI 2.3–9.2), *P* = 0.001; Table 2 and Fig. 2a). This was due to improvements in both symptoms (effect size for KCCQ-TS, 5.8 points (95% CI 2.0–9.6), *P* = 0.003) and physical limitations (effect size for KCCQ-PL, 5.3 points (95% CI 0.7–10.0), *P* = 0.026; Fig. 2b,c, respectively). The results were consistent within subgroups of patients with and without T2D, ejection fraction above and below 60% as well as across all other prespecified subgroups (Fig. 2d; all *P* values for interaction are nonsignificant).

**Table 2:** Primary and secondary endpoints at 12 weeks after treatment initiation

Continuous secondary endpoints	Dapagliflozin (*n* = 162)	Placebo (*n* = 162)	Effect size	*P* value
KCCQ-CS, mean^a^	68.6 (66.2, 71.0)	62.8 (60.4, 65.3)	5.8 (2.3, 9.2)	0.001
KCCQ-OS, mean^a^	68.9 (66.5, 71.3)	64.5 (62.1, 66.8)	4.5 (1.1, 7.8)	0.009
6MWT, mean, m^a^	262 (252, 272)	242 (232, 252)	20.1 (5.6, 34.7)	0.007
NTproBNP, mean, pg ml^–1^^a^	733 (673, 799)	739 (678, 805)	0.99 (0.88, 1.12)^b^	0.900
BNP, mean, pg ml^–1^^a^	147 (136, 160)	147 (136, 160)	1.00 (0.89, 1.12)^b^	0.990
Systolic blood pressure, mean, mmHg^a^	133 (130, 135)	133 (131, 136)	−0.6 (−4.4, 3.3)	0.780
Weight, mean, kg^a^	101.3 (100.9, 101.8)	102.1 (101.6, 102.6)	−0.72 (−1.42, −0.01)	0.046

Values are shown as adjusted means (95% CI) for continuous variables.

^a^Adjusted for the corresponding baseline value, history of T2D, sex, AF, baseline eGFR and LVEF.

^b^Ratio of dapagliflozin compared to placebo.

### Secondary endpoints

**Fig. 3:**
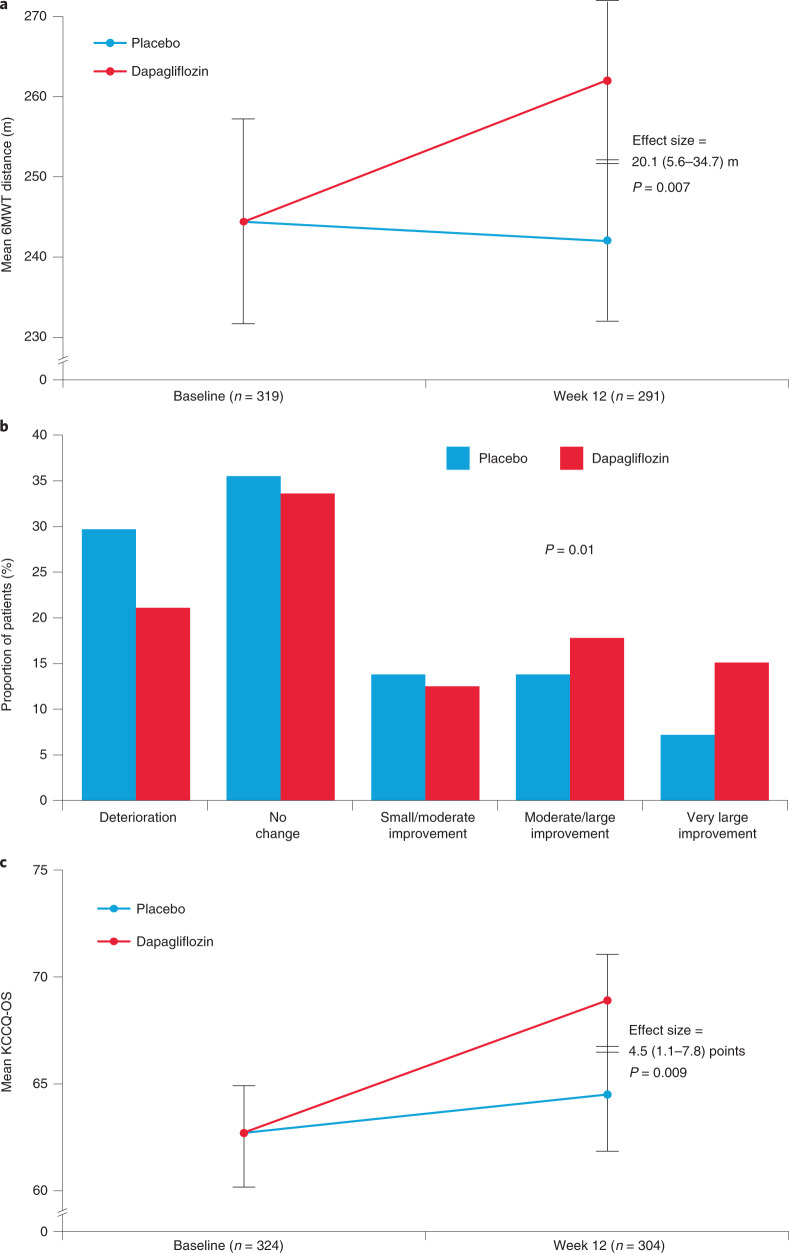
Effects of dapagliflozin on selected secondary endpoints and in supportive responder analysis. **a**–**c**, Effects of dapagliflozin on selected secondary endpoints and in supportive responder analysis. Effects of dapagliflozin versus placebo at 12 weeks on 6MWT distance (**a**), KCCQ-CS responder analysis (**b**) and KCCQ-OS (**c**). Data are presented as mean values with 95% CI. **a**,**c**, An *F*-test was used in data analysis; **b**, a chi-square test was used in data analysis All *P* values are two-sided, with no adjustments made for multiple comparisons.

The 6MWT results were available for 291 (89.8%) patients at 12 weeks (148 (91.4%) in the dapagliflozin group and 143 (88.3%) in the placebo group). Patients treated with dapagliflozin had an improvement in 6MWT distance at 12 weeks (effect size 20.1 m (95% CI 5.6–34.7), *P* = 0.007; Table 2 and Fig. 3a) that was proportionally large (8.2%) given the very low baseline value (244.4 m).

A numerically greater number of patients treated with dapagliflozin versus placebo had a 5-point or greater improvement in KCCQ-CS at 12 weeks (49.4 versus 38.2%; adjusted OR = 1.64 (95% CI 0.98–2.75), *P* = 0.06; Supplementary Table 1). In the supportive responder analysis, fewer patients treated with dapagliflozin versus placebo experienced deterioration or no change, while a greater proportion of patients treated with dapagliflozin experienced improvement in KCCQ-CS (*P* = 0.01; Fig. 3b).

Results were similar when using KCCQ-OS, with 45.4% of dapagliflozin-treated patients experiencing 5-point or greater improvement at 12 weeks versus 34.9% with placebo (adjusted OR = 1.73, 95% CI 1.05–2.85, *P* = 0.03; Supplementary Table 1). Mean KCCQ-OS was also higher with dapagliflozin versus placebo at 12 weeks (adjusted difference, 4.5 points (95% CI 1.1–7.8) versus placebo, *P* = 0.009; Table 2 and Fig. 3c).

Dapagliflozin also resulted in greater weight loss at 12 weeks (effect size, 0.72 kg (95% CI 0.01–1.42), *P* = 0.046; Table 2). There were no significant between-group differences in other secondary endpoints, including NTproBNP and BNP; proportion of patients with 20% or greater decrease in NTproBNP; proportion of patients with both a 5-point or greater increase in KCCQ-CS and 20% or greater decrease in NTproBNP; hemoglobin A1c (HbA1c); and systolic blood pressure at 12 weeks (Table 2 and Supplementary Table 1).

### Exploratory clinical endpoints

In total, nine patients (5.6%) in each of the treatment groups had adjudicated events of HF hospitalizations or urgent HF visits.

### Safety outcomes

The data regarding patients with reportable adverse events are shown in Table 3. One death occurred in the dapagliflozin group and two in the placebo group; all three were adjudicated as non-CV deaths. In the dapagliflozin and placebo groups, respectively, 44 (27.2%) and 38 (23.5%) patients had reported adverse events; 31 (19.1%) and 22 (13.6%) had serious adverse events; 18 (11.1%) and 15 (9.3%) had adverse events resulting in discontinuation of study medication; and 7 (4.3%) versus 8 (4.9%) had drug adverse events (those considered by the investigator to be due to, and resulting in, permanent discontinuation of the investigational product). Adverse events of volume depletion were reported in 11 (6.8%) versus 7 (4.3%) patients; and acute kidney injury in 5 (3.1%) versus 5 (3.1%) in the dapagliflozin and placebo groups, respectively. No events of diabetic ketoacidosis (DKA), severe hypoglycemia or lower limb amputation occurred during the trial.

**Table 3:** Safety analyses

	Dapagliflozin (*n* = 162)	Placebo (*n* = 162)
All reported adverse events	44 (27.2%)	38 (23.5%)
Serious adverse events	31 (19.1%)	22 (13.6%)
Adverse events resulting in discontinuation of study medication	18 (11.1%)	15 (9.3%)
Drug adverse events	7 (4.3%)	8 (4.9%)
All-cause death	1 (0.6%)	2 (1.2%)
Nonfatal MI	0 (0%)	1 (0.6%)
Stroke	0 (0%)	1 (0.6%)
Acute kidney injury	5 (3.1%)	5 (3.1%)
DKA	0 (0%)	0 (0%)
Volume depletion events	11 (6.8%)	7 (4.3%)
Severe hypoglycemic events	0 (0%)	0 (0%)
Lower limb amputations	0 (0%)	0 (0%)

Values are shown as absolute numbers (percentages) for patients with events.

## Discussion

In this multicenter, double-blind, randomized, placebo-controlled trial in patients with HFpEF, treatment with dapagliflozin improved HF-related symptoms and physical limitations as measured by KCCQ-CS after only 12 weeks of treatment. The magnitude of these benefits was clinically meaningful, statistically significant and consistent across all prespecified subgroups, including patients with and without T2D and those with ejection fraction above and below 60%. Patients treated with dapagliflozin also had a significant, clinically meaningful 20-m improvement in 6MWT distance. To our knowledge this may represent the first clinical trial to show a significant benefit of SGLT2 inhibitors on both patient-reported symptoms and physical limitations, as well as objectively measured physical function, in individuals with HFpEF.

Improvement in symptoms and physical function in HFpEF are key goals of management, given that this population has especially poor health status^16^. Although SGLT2 inhibitors have been shown to improve symptoms in individuals with HFrEF^10–12^, their effects on health status in symptomatic individuals with chronic HFpEF are not established^15,17^. The magnitude of benefit (5.8- and 4.5-point improvement in KCCQ-CS and KCCQ-OS, respectively) with dapagliflozin is especially notable, as previous therapies tested in HFpEF have generally not produced a clinically meaningful improvement in health status. Specifically, in the TOPCAT trial, treatment with spironolactone resulted in a 1.5-point improvement in KCCQ-OS versus placebo and, in the PARAGON trial, treatment with sacubitril valsartan resulted in a 1-point higher KCCQ-OS compared with valsartan^18,19^. Our results are further buttressed by the findings in responder analyses using KCCQ-CS, which indicated that dapagliflozin-treated patients were more likely to experience a very large (20-point or greater) improvement in health status and less likely to have worsening health status.

These results were consistent within subgroups of patients with and without T2D, as well as across all other prespecified subgroups. Of note, our study population was diverse with 57% women and 30% African American participants—consistent with the population-level demographic characteristics of individuals with HFpEF in the United States^1,2^. Notably, the health status benefits of dapagliflozin were consistent in each of these important subgroups that have traditionally been under-represented in HF trials and that have a great need for efficacious therapies. The use of baseline medical therapies observed in PRESERVED-HF was overall similar to that seen in several large, contemporary HFpEF trials. Specifically, >99% of participants in PRESERVED-HF were receiving diuretics (versus 86 and 96% in EMPEROR-PRESERVED and PARAGON-HF, respectively) and 36% of participants in PRESERVED-HF were receiving mineralocorticoid receptor antagonists, a rate that was greater than in PARAGON-HF (24%) and similar to that in EMPEROR-PRESERVED (37%)^15,19,20^.

Our results differ from one previous trial of similar size and treatment duration^21^. The EMPERIAL-PRESERVED trial evaluated the effects of the SGLT2 inhibitor empagliflozin on symptoms and functional status in HFpEF and reported a nonsignificant, 2.0-point increase in KCCQ-TS with empagliflozin versus placebo, in contrast to the larger (and highly statistically significant) 5.8-point improvement in both KCCQ-TS and KCCQ-CS in the present study^21^. EMPERIAL-PRESERVED also reported a nonsignificant 4-m improvement in 6MWT distance with empagliflozin versus placebo, while we observed a much larger (and statistically significant) 20-m improvement with dapagliflozin versus placebo. The 5.8-point improvement in KCCQ-CS that was observed in PRESERVED-HF is also larger than the 1.3-point difference in KCCQ-CS seen with empagliflozin versus placebo in EMPEROR-PRESERVED, a large global outcome trial that demonstrated a significant reduction in the primary endpoint of CV death or hospitalizations for HF in participants with chronic HFpEF^15^. However, it should be noted that KCCQ-CS was assessed at 1 year in EMPEROR-PRESERVED versus at 12 weeks in both EMPERIAL-PRESERVED and PRESERVED-HF.

One potential explanation for these discrepancies between EMPERIAL-PRESERVED, EMPEROR-PRESERVED and PRESERVED-HF may be differences in baseline characteristics of the study participants. Specifically, PRESERVED-HF (versus EMPERIAL-PRESERVED and EMPEROR-PRESERVED) included a higher proportion of participants who were women (58 versus 43 and 45%, respectively) and African American (30 versus 10 and 4%), with notably higher BMI (34.7 versus 29.6 and 29.8 kg m^–2^), all characteristics that more closely match those of patients with HFpEF in the US population^1^. Participants in PRESERVED-HF (versus those in EMPERIAL-PRESERVED and EMPEROR-PRESERVED) also had a considerably greater degree of symptomatic and functional impairment as measured by NYHA class (42 versus 22 and 20% with NYHA class III/IV, respectively), and by 6MWT distance (244 versus 298 m in EMPERIAL-PRESERVED but not assessed in EMPEROR-PRESERVED), characteristics that have been strongly linked to poorer quality of life^8^. PRESERVED-HF (as compared with EMPERIAL-PRESERVED, but not with EMPEROR-PRESERVED) also included a higher propotion of participants who required loop-diuretic therapy (88 versus 72 and 86%, respectively) and a higher proportion of participants with AF (53 versus 30 and 52%), which is associated with poorer cardiac reserve and more right-sided HF and pulmonary hypertension^22^. Importantly, our trial was also conducted exclusively at sites in the United States whereas EMPERIAL-PRESERVED enrolled patients from Western and Eastern Europe, Australia and Canada, as well as from the United States, and EMPEROR-PRESERVED had global participation. While it is possible that there are pharmacodynamic differences between empagliflozin and dapagliflozin, this explanation seems less likely as both agents have been found to provide similar benefits on the composite of CV death or hospitalization for HF in large outcome trials of individuals with HFrEF^11,12^.

The observed improvement in 6MWT is relatively unique and highly clinically relevant. Even when patients with HFpEF are stable and well compensated, they have a markedly impaired objectively measured physical function^23^. Impaired physical function is an independent predictor of poorer quality of life, hospitalizations, loss of independence, nursing home placement and death. Formal patient interviews indicate that patients with HF value improved physical function at least equally with avoidance of death^24,25^. To date, 12 trials have formally tested a variety of medications in HFpEF for exercise function outcomes, as measured by 6MWT or cardiopulmonary exercise testing, and nearly all have been neutral^23^. Furthermore, all five classes of agents proven to improve clinical events in HFrEF have minimal impact on exercise function. The magnitude of the increase in 6MWT distance that we observed is proportionally large (8.2%) and is greater than that observed in the HF-ACTION trial of exercise training in HFrEF, where it was associated with an improvement in clinical events, and is similar in magnitude to that observed in exercise training trials of HFpEF^26,27^.

Several potential mechanisms may explain the clinical benefits of dapagliflozin we observed in this trial. First, SGLT2 inhibitors have been shown to rapidly lower pulmonary artery pressure, which aids decongestion and can translate to improvements in both symptoms and exercise function^28,29^. Second, SGLT2 inhibitors may increase myocardial energy production; alleviate systemic microvascular dysfunction, which is prevalent in both the myocardium and skeletal muscle in HFpEF; improve systemic endothelial function; reduce systemic inflammation and oxidative stress; and improve insulin sensitivity and activate fatty acid oxidation in the skeletal muscle^30–33^. Finally, SGLT2 inhibitors also result in modest weight loss. This is of relevance given the high prevalence of overweight/obesity among patients with HFpEF (>80%) in the United States, which was clearly reflected in our trial with an average baseline BMI of 35 (ref. ^34^).

Consistent with previous data from comparably sized trials, dapagliflozin treatment had no significant effect on natriuretic peptides^10^. Although natriuretic peptide levels are predictive of prognosis in HFpEF, they are known to be considerably lower than in HFrEF and previous studies have shown little relationship between natriuretic peptides and health status^8,35^. Future HFpEF trials may benefit from focusing on patient-centered outcomes such as health status and exercise function that are more relevant to a patient’s journey, as was done in PRESERVED-HF. Dapagliflozin’s tolerability profile was also generally consistent with previous SGLT2 inhibitor trials, with no new safety signals identified. Reassuringly, we did not observe any events of DKA or severe hypoglycemia (although about half of the patients did not have T2D and the duration of the study was short).

Despite the strengths in study design and execution, our findings should be interpreted in the context of several potential limitations. First, its relatively short duration of follow-up precludes assessment regarding the durability of the observed benefit on HF-disease-specific symptoms or functional status. Second, all patients were enrolled at sites in the United States and, while this makes the study applicable to the US population with HFpEF, its generalizability outside that country is uncertain. Additional data from global trials of SGLT2 inhibitors, including the recently completed EMPEROR-PRESERVED and the ongoing DELIVER trial, will ultimately address some of these limitations. Nevertheless, even the short-term improvements in health status and exercise function shown here with dapagliflozin represent an important therapeutic advance given the lack of available effective treatments for HFpEF.

In conclusion, dapagliflozin significantly improved symptoms, physical limitations and objectively measured exercise function in patients with HFpEF. The magnitude of treatment benefits was large, clinically meaningful and statistically significant, and consistent across all prespecified subgroups.

## Methods

**Extended Data Fig. 1:**
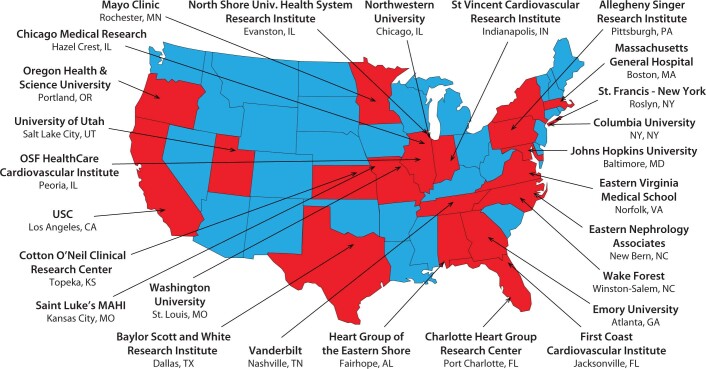
Map of the study sites.

PRESERVED-HF was a randomized, double-blind, placebo-controlled, multicenter trial of individuals with chronic, symptomatic HFpEF (Supplementary Table 2). The trial was conducted across 26 sites in the United States. The list of participating sites and investigators is given in Supplementary Table 3 and Extended Data Fig. 1. The primary outcome was HF-disease-specific health status as assessed by KCCQ-CS^36^. The study protocol is provided in the Supplementary Data.

PRESERVED-HF was an investigator-initiated trial with the concept developed, and the trial sponsored and executed by, the national coordinating center at Saint Luke’s Mid America Heart Institute in collaboration with the Executive Committee (Supplementary Table 4), independent of the funding source (AstraZeneca). The study was monitored by an independent data safety and monitoring committee. Institutional review boards approved the study for all sites (Supplementary Table 3 and Extended Data Fig. 1), and all patients provided informed consent for research participation. The trial was conducted in accordance with the ICH E6(R1) Guidelines of Good Clinical Practice and the Declaration of Helsinki.

### Patient selection

Full inclusion and exclusion criteria can be found in Supplementary Table 5. Adult ambulatory patients with or without T2D, clinical diagnosis of HFpEF, LVEF ≥ 45% and NYHA class II–IV symptoms were screened for participation. Patients additionally had to have elevated natriuretic peptides (NTproBNP ≥ 225 or BNP ≥ 75 pg ml^–1^; if AF, NTproBNP ≥ 375 pg ml^–1^ or BNP ≥ 100 pg ml^–1^); requirement for diuretic therapy (loop, thiazide or potassium-sparing diuretics) and either HF hospitalization or urgent HF visit with intravenous diuretic treatment in the past 12 months; documented elevated filling pressures on right or left heart catheterization; or echocardiographic evidence of structural heart abnormalities. Key exclusion criteria were recent hospitalization (within 7 days) for decompensated HF, eGFR <20 ml min^–1^ 1.73 m^–2^ at the screening visit, type 1 diabetes or previous history of DKA.

### Trial design

**Extended Data Fig. 2:**
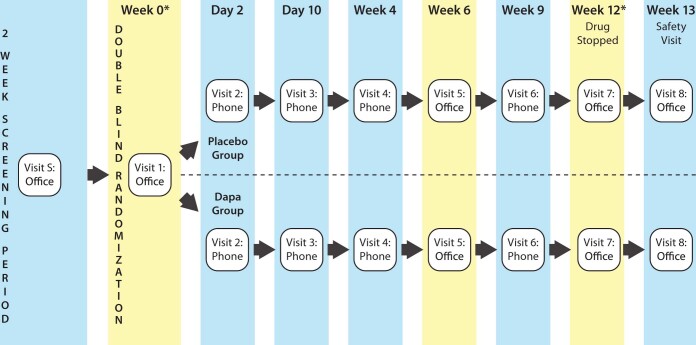
Trial design and schedule of study visits.

Patients considered potentially eligible and who agreed to participate and provided informed consent entered a 2-week screening phase during which their eligibility was confirmed based on central core laboratory evaluation (NTproBNP ≥ 225 pg ml^–1^ or BNP ≥ 75 pg ml^–1^; higher if AF), eGFR ≥ 20 ml min^–1^ 1.73 m^–2^ and clinical stability was ensured. Patients confirmed as eligible were randomized in a double-blind fashion—1:1 to oral dapagliflozin 10 mg or matching placebo once daily (Extended Data Fig. 2). Before administration of the first dose of dapagliflozin or placebo, patients underwent a physical examination, trial-related laboratory assessments, completion of the KCCQ and a 6MWT. Patients then entered a 12-week treatment period during which they were followed via four telephone call visits as well as two in-person study visits (at 6 and 12 weeks), at which times repeat physical examination, laboratory assessment (at 6 and 12 weeks), KCCQ and 6MWT were completed (at 12 weeks). At week 12, study medication was discontinued and patients were followed for one additional week, at the end of which another in-person study visit was conducted to assess for any intercurrent safety events, and to collect additional laboratory samples.

### Outcomes

The primary outcome was KCCQ-CS at 12 weeks. KCCQ-CS was originally a key secondary endpoint, with the original primary outcome defined as mean change in NTproBNP from baseline to 6 and 12 weeks. However, during the trial, compelling external scientific information from a related trial of dapagliflozin in HFrEF showed a greater benefit on patient-reported symptoms and physical limitations (measured using the KCCQ)—outcomes that are more meaningful to patients and clinicians than change in NTproBNP^10^. Accordingly, in March 2020 (17 months before database lock) the protocol was amended to elevate the KCCQ-CS at 12 weeks as the primary endpoint. This decision was made solely by the excutive committee, which was fully blinded at the time.

The KCCQ is a standardized, 23-item, self-administered instrument that quantifies HF-related symptoms (frequency, severity and recent change), physical function, quality of life and social function. KCCQ-CS includes symptoms and physical function (domains considered most likely to be modified by SGLT2 inhibitors). For each domain, the validity, reproducibility, responsiveness and interpretability have been independently established. Scores are transformed to a range of 0–100, in which higher scores reflect better health status^36^.

The key secondary outcome was the 6MWT, which was conducted according to well-established methods^37^. Other secondary outcomes included the proportion of patients with meaningful (5-point or greater) change in KCCQ-CS and -OS; 6MWT distance; NTproBNP and BNP; proportion of patients with 20% or greater decrease in NTproBNP and BNP; proportion of patients with both 5-point or greater increase in KCCQ-CS and 20% or greater decrease in NTproBNP; HbA1c; and weight and systolic blood pressure at 12 weeks. Levels of NTproBNP and BNP, and all other study laboratory assessments, were analyzed at a Quest Diagnostics central laboratory, blinded to treatment assignment (NTproBNP assay Roche electrochemiluminescent method on Elecsys platform, ProBNP II reagent by Roche/Cobas; BNP assay chemiluminescent method on Siemens ADVIA Centaur platform). Exploratory endpoints included the number of hospitalizations for HF or urgent HF visits (Supplementary Table 2).

All serious adverse events were reported by investigators. An independent, blinded clinical events committee adjudicated all deaths, hospitalizations for HF, urgent HF visits, myocardial infraction (MI) and transient ischemic attack/stroke events. In addition, investigator-reported adverse events of special interest included acute kidney injury (defined as doubling of serum creatinine), DKA, volume depletion, severe hypoglycemia (defined as blood glucose <54 mg dl^–1^ (<3.0 mmol l^–1^) and requiring assistance) and lower limb amputations.

### Concomitant medications

The trial was designed to enroll patients receiving standard-of-care therapy for HFpEF. In patients with T2D, plans for reducing the risk of hypoglycemia included a suggested 20% reduction in the total daily dose of insulin and/or 50% reduction in the total daily dose of insulin secretagogs (that is, sulfonylurea and metiglinides) for patients with baseline HbA1c ≤ 7%.

### Statistical analysis

Patient disposition is reported, including all patients who signed the informed consent. All primary and secondary efficacy endpoints were evaluated using the modified intention-to-treat dataset, which included all randomized patients who received at least one dose of study medication and had at least one evaluable endpoint. Patients with no evaluable follow-up data for a particular outcome (for example, NTproBNP) were excluded from those respective analyses. The safety analysis set included all patients who received at least one dose of study medication, and was used for all safety analyses.

Continuous measures were summarized by mean ± s.d. or median and IQR, and compared using Student’s *t*-test or Wilcoxon rank-sum test, as appropriate. Categorical variables were summarized by frequency and percentage and compared using chi-square or Fisher’s exact test, as appropriate.

For the primary endpoint, an analysis of covariance model was used to estimate the effect of dapagliflozin relative to placebo on the 12-week KCCQ-CS, adjusting for baseline value, sex, eGFR, T2D status, AF status and LVEF. Restricted cubic splines were included for continuous variables to accommodate nonlinear effects. Supportive analyses were performed examining the effects of dapagliflozin on the components of the primary endpoint (KCCQ-TS and-PL). Several subgroup analyses were prespecified, including age (<70, ≥70 years), sex (male, female), race, T2D status, BMI (< median, ≥ median), baseline NTproBNP (<median, ≥median), baseline LVEF (≤60%, >60%), AF status, baseline KCCQ-OS (<median, ≥median), baseline eGFR (<60, ≥60 ml min^–1^ 1.73 m^–2^), baseline loop-diuretic dose (furosemide equivalent mean daily dose ≤4 mg, >40 mg), and NYHA class (II, III–IV).

For secondary endpoints, KCCQ-OS was analyzed in a manner analogous to that of the primary endpoint. The unadjusted proportion of patients achieving ≥5-point improvement in KCCQ-CS and -OS at 12 weeks was calculated for the treatment and placebo groups, and a logistic regression model was used to assess the treatment effect adjusted for baseline value, sex, eGFR, T2D, AF and LVEF. A post hoc supportive analysis also compared the proportions of patients that had deterioration (>5-point worsening) or no change, as well as small/moderate (5- to <10-point), moderate/large (10- to <20-point) and very large (20-point or greater) improvement, in KCCQ-CS between dapagliflozin- and placebo-treated participants, using a Mantel-Haenszel chi-square test. A generalized linear mixed model was used to estimate the treatment effect on 6- and 12-week NTproBNP and BNP values, adjusting for log baseline NTproBNP (or BNP), sex, eGFR, T2D, AF and LVEF, with patient included as a random effect and gamma distribution and log link function used to account for the skewed nature of NTproBNP (or BNP). HbA1c, weight and systolic blood pressure were analyzed in a manner analogous to that of natriuretic peptides, although normal distribution and identity link function were used. Analyses were tested at a two-sided significance level of 5%, without adjustment for multiple comparisons.

Safety outcomes were assessed using descriptive statistics only, and no *P* values were calculated. The same approach was used for the number of HF hospitalizations or urgent HF visits.

SAS v.9.4 was used for all analyses (SAS Institute).

### Sample size calculations

For the primary endpoint, a sample size of 145 for each group was estimated to achieve 82% power with *α* = 0.05 to detect a 4.7-point difference in mean KCCQ-CS between dapagliflozin and placebo groups at 12 weeks. The assumptions for this calculation were derived from the DEFINE-HF trial where the mean difference between dapagliflozin and placebo groups was 4.7 points with s.d. = 13.7 (ref. ^10^). Assuming a 10% loss to follow-up, we arrived at a sample size of ~320 patients.

### Reporting Summary

Further information on research design is available in the Nature Research Reporting Summary linked to this article.

## Online content

Any methods, additional references, Nature Research reporting summaries, source data, extended data, supplementary information, acknowledgements, peer review information; details of author contributions and competing interests; and statements of data and code availability are available at 10.1038/s41591-021-01536-x.

## Supplementary information

Supplementary Information Supplementary Tables 1–5.

Reporting Summary

Supplementary Data PRESERVED-HF protocol.

## Data Availability

Deidentified participant data will be made available on reasonable request 2 years after the date of publication. Requests should be directed to the corresponding author (mkosiborod@saint-lukes.org). Requestors will be required to sign a data access agreement to ensure the appropriate use of the study data.
