# Supplementary material for: The SGLT2 inhibitor dapagliflozin in heart failure with preserved ejection fraction: a multicenter randomized trial
Source: Nat Med. 2021 Oct 28;27(11):1954–60. doi: 10.1038/s41591-021-01536-x (PMC8604725; doi:10.1038/s41591-021-01536-x)
Supplement: Supplementary file 1 — Supplementary Tables 1–5. [file 41591_2021_1536_MOESM1_ESM.pdf]

nature portfolio

https://doi.org/10.1038/s41591-021-01536-x

Supplementary information

The SGLT2 inhibitor dapagliflozin in heart failure with preserved ejection fraction: a multicenter randomized trial

In the format provided by the authors and unedited

SUPPLEMENTARY INFORMATION

Table of Contents

Supplementary Table 1. Outcomes analyzed as binary endpoints at 12 weeks
Supplementary Table 2. Abbreviated protocol synopsis
Supplementary Table 3. List of participating sites and investigators
Supplementary Table 4. Executive Committee
Supplementary Table 5. Full inclusion and exclusion criteria

Supplementary Table 1. Outcomes analyzed as binary endpoints at 12 weeks

Binary Secondary Endpoints	Dapagliflozin (n = 162)	Placebo (n = 162)	Adjusted Odds Ratio	95% CI	P value
**KCCQ CS increase ≥5 points**	69 (45.4%)	53 (34.8%)	1.64	0.98, 2.75	0.06
**KCCQ OS increase ≥5 points**	75 (49.4%)	58 (38.2%)	1.73	1.05, 2.85	0.03
**NT-proBNP decrease ≥20%**	48 (32.0%)	44 (29.3%)	1.2	0.72, 2.01	0.48
**BNP decrease ≥20%**	50 (33.8%)	49 (33.1%)	1.1	0.66, 1.81	0.74
**KCCQ CS increase ≥5 points and NT-proBNP decrease ≥20%**	23 (15.5%)	15 (10.0%)	1.94	0.90, 4.15	0.09

Values are shown as absolute numbers (percentages) for the binary outcomes, with odds ratios and 95 percent confidence intervals; adjusted for corresponding baseline value, history of Type 2 diabetes, sex, AF, baseline eGFR and LVEF.

KCCQ OS, Kansas City Cardiomyopathy Questionnaire overall summary score; KCCQ CS, Kansas City Cardiomyopathy Questionnaire clinical summary score summary score; NT-proBNP, N-Terminal Pro B-Type Natriuretic Peptide; BNP, B-Type Natriuretic Peptide;

Supplementary Table 2. Abbreviated Protocol Synopsis

A 12-week randomized, double-blind, placebo-controlled trial to evaluate the effects of once-daily dapagliflozin 10 mg on heart failure disease-specific health status in patients with chronic heart failure with preserved ejection fraction

Study Hypothesis

Treatment with dapagliflozin 10 mg daily for 12 weeks will produce greater improvement in health status as compared with placebo in patients with chronic heart failure with preserved ejection fraction

Study Centers and Number of Patients Proposed

This study will be performed at up to 35 centers in the United States. Approximately 320 patients will be randomized over a target enrollment period of approximately 36 months.

Primary Objective

To evaluate the impact of dapagliflozin on heart failure disease-specific status, and quality of life in patients with chronic heart failure with preserved ejection fraction.

Target Population

Male and female patients with chronic heart failure with preserved systolic function.

Investigational Product, Dosage, and Mode of Administration

Dapagliflozin 10 mg administered orally once daily for 12 weeks, in addition to standard of care for chronic heart failure

Comparator, Dosage and Mode of Administration

Matching placebo administered orally once daily for 12 weeks, in addition to standard of care

Study Duration

After activation of the first site, it is expected that enrolment will take approximately 36 months. After randomization, dapagliflozin or placebo will be administered for 12 weeks. Renal function will be evaluated 1 week after discontinuation of dapagliflozin or placebo.

Primary Outcome Variables (two co-primary endpoints will be evaluated)

1. Change from baseline in the Kansas City Cardiomyopathy Questionnaire Clinical Summary Score (KCCQ-CS) at 12 weeks.

Secondary Outcome Variables

1. Change from baseline in KCCQ Overall Summary (KCCQ-OS) score over 12 weeks
2. Change from baseline in NTproBNP over 12 weeks
3. Change from baseline in BNP over 12 weeks
4. Change in 6-minute walk distance over 12 weeks.
5. Proportion of patients with a ≥ 5 point increase in KCCQ-CS.
6. Proportion of patients with a ≥ 5 point increase in KCCQ-OS.
7. Proportion of patients with a ≥ 20% decrease in NT-proBNP.
8. Proportion of patients with a ≥ 5 point increase in KCCQ and a ≥ 20% decrease in NT-proBNP.
9. Change from baseline in HbA1c over 12 weeks
10. Change from baseline in weight over 12 weeks.
11. Change from baseline in systolic blood pressure over 12 weeks.

Safety Variables

1. All cause death
2. Cardiovascular death
3. Non-fatal myocardial infarction (MI)
4. Stroke
5. Acute kidney injury (defined as doubling of serum creatinine based on the modified RIFLE criteria)
6. Adverse events (AEs) and serious adverse events (SAEs). AEs of special interest will include DKA, volume depletion (defined as hypotension, syncope, orthostatic hypotension or dehydration), severe hypoglycemic events and lower limb amputations.

Supplementary Table 3. Study Sites and Investigators

Institution	Principal Investigators
Saint Luke's Mid America Heart Institute	Taiyeb Khumri, MD
Emory University	Guillermo Umpierrez, MD
First Coast Cardiovascular Institute	Sumant Lamba, MD
The Johns Hopkins Hospital	Kavita Sharma, MD
Northwestern University	Sadiya S. Khan, MD
Chicago Medical Research	Lokesh Chandra, MD
NorthShore University	Robert A. Gordon, MD
University of Utah	John J. Ryan, MD
St. Vincent Heart Center	Sunit-Preet Chaudhry, MD
Mayo Clinic	Barry A. Borlaug, MD
Baylor Scott & White Health	Susan M. Joseph, MD<br>Cesar Guerrero Miranda, MD
Stormont Vail Health	Chen H. Chow, MD
Wake Forest University	Dalane W. Kitzman, MD
Allegheny Health Network	Manreet K. Kanwar, MD
Heart Group of the Eastern Shore	Michael Pursley, MD
Eastern Virginia Medical School	Elias S. Siraj, MD
Massachusetts General Hospital	Gregory D. Lewis, MD
OSF Healthcare Cardiovascular Institute	Barry S. Clemson, MD
University of Southern California	Michael Fong, MD
Washington University	Justin Vader, MD
Eastern Nephrology Associates	Manuel Montero, MD
Charlotte Heart Group Research Center	Ricardo Martinez, MD
Columbia University	Mathew Maurer, MD
Vanderbilt University	Deepak Gupta, MD
St Francis Hospital	Rita Jermyn, MD
Oregon Health & Science University	Samuel Camacho, MD

Additional Investigators

Saint Luke's Mid America Heart Institute	Michael E. Nassif, MD<br>Yevgeniy Khariton, MD<br>Ali O. Malik, MD
First Coast Cardiovascular Institute	Brenda Murphy, MD
Northwestern University	Jay Pandit, MD
Chicago Medical Research	Eden Brandon, MD
Stormont Vail Health	Emily Bohannon, PA<br>Rachel Moats, PA
Wake Forest University	Bharathi Upadhya, MD
Eastern Virginia Medical School	John E. Brush, MD<br>Aaron B. Nelson, MD
OSF Healthcare Cardiovascular Institute	Christopher Sparrow, MD<br>Chetan Bhardwaj, MD

Supplementary Table 4. Executive Committee

Member Name	Institution
Mikhail N. Kosiborod, MD (Chair)	Saint Luke’s Mid America Heart Institute
Barry A. Borlaug, MD	Mayo Clinic
Dalane W. Kitzman, MD	Wake Forest School of Medicine
Sanjiv J. Shah, MD	Northwestern University Feinberg School of Medicine

Supplementary Table 5. Full Inclusion and Exclusion Criteria

Inclusion criteria

1. Age > 18 and < 120 at the screening visit
2. Symptoms of dyspnea (NYHA class II-IV) without evidence of a non-cardiac or ischemic explanation for dyspnea
3. Ejection fraction (EF) ≥ 45% as determined on imaging study within 24 months of enrolment with no change in clinical status suggesting potential for deterioration in systolic function
4. Elevated NT-proBNP (≥ 225 pg/ml) or BNP (≥ 75 pg/ml) Ŧ
5. Stable medical therapy for heart failure for 15 days as defined by:
   i. No addition or removal of ACE, angiotensin receptor blockers (ARBs), valsartan/sacubitril, beta-blockers, calcium channel blockers (CCBs) or aldosterone antagonists
   ii. No substantial change in dosage (100% or greater increase or decrease from baseline dose) of ACE, ARBs, beta-blockers, CCBs or aldosterone antagonists
6. On a diuretic ≥15 days prior to screening visit and a stable diuretic therapy for 7 days
7. At least one of the following:
   i. Hospitalization for decompensated HF in the last 12 months
   ii. Acute treatment for HF with intravenous loop diuretic or hemofiltration in the last 12 months
   iii. Mean pulmonary capillary wedge pressure ≥15 mmHg or LV end diastolic pressure (LVEDP) ≥15 mmHg documented during catheterization at rest, or pulmonary capillary wedge pressure or LVEDP ≥25 mmHg documented during catheterization with exercise.
   iv. Structural heart disease evidenced by at least one of the following echo findings (any local measurement made within the 24 months prior to screening visit):
      1) left atrial (LA) enlargement defined by at least one of the following: LA width ≥3.8cm or LA length ≥5.0 cm **or** LA area ≥20 cm2 **or** LA volume ≥55 mL **or** LA volume index ≥29 mL/m2
      2) OR left ventricular hypertrophy (LVH) defined by septal thickness or posterior wall thickness ≥1.1 cm.

Exclusion criteria

1. Decompensated heart failure (hospitalization for heart failure within 7 days prior to screening)
2. History of type 1 diabetes
3. History of diabetic ketoacidosis
4. Estimated glomerular filtration rate (eGFR) < 20 at the screening visit by modified MDRD equation GFR (mL/min/1.73 m2) = 175 x (Scr) $^{-1.154}$ x (Age$^{)-0.203}$ x (0.742 if female) x (1.210 if African American)
5. Admission for an acute coronary syndrome (ST-elevation MI, non-ST-elevation MI, or unstable angina), percutaneous coronary intervention, or cardiac surgery within 30 days prior to the screening visit.
6. Admission for cardiac resynchronization therapy (CRT) within 90 days prior to the screening visit.
7. Planned cardiovascular revascularization (percutaneous intervention or surgical) or major cardiac surgery (coronary artery bypass grafting, valve replacement, ventricular assist device, cardiac transplantation, or any other surgery requiring thoracotomy, or transcatheter aortic valve replacement) or CRT within the 90 days after the screening visit.
8. Participation in any interventional clinical trial (with an investigational drug or device) that is not an observational registry within 15 days of the screening visit.
9. History of hypersensitivity to dapagliflozin
10. For women of child-bearing potential: Current or planned pregnancy or currently lactating.

    Women of childbearing potential are defined as any female who has experienced menarche and who is NOT permanently sterile or postmenopausal. Post menopausal is defined as 12 consecutive months with no menses without an alternative medical cause. Women of child-bearing potential, who are sexually active, must agree to use a medically-accepted method of birth control for the duration of the study. Acceptable birth control methods include: (1) surgical sterilization (such as a hysterectomy or bilateral tubal ligation), (2) progesterone hormonal contraceptives (birth control pills or implants), (3) barrier methods (such as a condom or diaphragm) used with a spermicide, or (4) an intrauterine device (IUD). Women of child-bearing potential will have a urine pregnancy test at every clinic visit and it must be negative to continue study participation.

11. Life expectancy <1 year at the screening visit
12. Patients who are volume depleted based upon physical examination at the time of the screening or randomization visit
13. BNP <75 pg/mL and NTproBNP<225 pg/mL at the screening visit [£]
14. Patients currently being treated with any SGLT-2 inhibitor (dapagliflozin, canagliflozin, empagliflozin, ertugliflozin) or having received treatment with any SGLT-2 inhibitor within the 12 weeks prior to the screening visit.
15. Average supine systolic BP <100 mmHg at the screening or randomization visit
16. Current history of bladder cancer
17. Donation of blood or bone marrow 12 weeks prior to the screening visit and no planned donations during the study period

18. Heart failure due to restrictive/infiltrative cardiomyopathy, active myocarditis, constrictive pericarditis, severe stenotic valve disease, and HOCM (hypertrophic obstructive cardiomyopathy).
19. Heart failure due to severe aortic or mitral regurgitation
20. Severe COPD thought to be a primary contributor to dyspnea
21. Isolated right heart failure due to pulmonary disease
22. Active and significant ischemia thought to be a primary contributor to dyspnea
23. Documentation of previous EF < 45%, under stable conditions, within the past 36 months
24. Complex congenital heart disease
25. Uncontrolled hypertension, defined as systolic blood pressure ≥200 mmHg during the screening visit (average value of three blood pressure measurements obtained in supine position)
26. Any other condition that in the judgment of the investigator would jeopardize the patient's participation in the study or that may interfere with the interpretation of study data or if the patient is considered unlikely to comply with study procedures, restrictions and requirements
27. Bariatric surgery within the past 6 months or planned bariatric surgery within the study time course.
28. CardioMems device implantation within previous 4 weeks or planned CardioMems implantation during study period
29. For echo substudy only: patients with ventricular paced rhythm or left bundle branch block on the most recent clinically available 12-lead electrocardiogram.
30. For echo substudy only: permanent atrial fibrillation

[Ŧ] For patients with permanent atrial fibrillation inclusion thresholds will be BNP ≥ 100 pg/mL or NTproBNP ≥ 375 pg/mL

[£]For patients with permanent atrial fibrillation exclusion thresholds will be BNP<100 pg/mL and NTproBNP<375pg/mL
