# Supplementary material for: The SGLT2 inhibitor dapagliflozin in heart failure with preserved ejection fraction: a multicenter randomized trial
Source: Nat Med. 2021 Oct 28;27(11):1954–60. doi: 10.1038/s41591-021-01536-x (PMC8604725; doi:10.1038/s41591-021-01536-x)
Supplement: Supplementary file 2 — Reporting Summary [file 41591_2021_1536_MOESM2_ESM.pdf]

nature research

Corresponding author(s): Mikhail Kosiborod, MD

Last updated by author(s): Sep 10, 2021

Reporting Summary

Nature Research wishes to improve the reproducibility of the work that we publish. This form provides structure for consistency and transparency in reporting. For further information on Nature Research policies, see our Editorial Policies and the Editorial Policy Checklist.

Statistics

For all statistical analyses, confirm that the following items are present in the figure legend, table legend, main text, or Methods section.

n/a	Confirmed	
☐	☒	The exact sample size (*n*) for each experimental group/condition, given as a discrete number and unit of measurement
☐	☒	A statement on whether measurements were taken from distinct samples or whether the same sample was measured repeatedly
☐	☒	The statistical test(s) used AND whether they are one- or two-sided<br>*Only common tests should be described solely by name; describe more complex techniques in the Methods section.*
☐	☒	A description of all covariates tested
☐	☒	A description of any assumptions or corrections, such as tests of normality and adjustment for multiple comparisons
☐	☒	A full description of the statistical parameters including central tendency (e.g. means) or other basic estimates (e.g. regression coefficient) AND variation (e.g. standard deviation) or associated estimates of uncertainty (e.g. confidence intervals)
☐	☒	For null hypothesis testing, the test statistic (e.g. *F*, *t*, *r*) with confidence intervals, effect sizes, degrees of freedom and *P* value noted<br>*Give P values as exact values whenever suitable.*
☒	☐	For Bayesian analysis, information on the choice of priors and Markov chain Monte Carlo settings
☒	☐	For hierarchical and complex designs, identification of the appropriate level for tests and full reporting of outcomes
☐	☒	Estimates of effect sizes (e.g. Cohen's *d*, Pearson's *r*), indicating how they were calculated

Our web collection on statistics for biologists contains articles on many of the points above.

Software and code

Policy information about availability of computer code

Data collection	REDCap 10.6.1 electronic CRF / ALEA v15.15851.201736 electronic randomization tool
Data analysis	SAS 9.4

For manuscripts utilizing custom algorithms or software that are central to the research but not yet described in published literature, software must be made available to editors and reviewers. We strongly encourage code deposition in a community repository (e.g. GitHub). See the Nature Research guidelines for submitting code & software for further information.

Data

Policy information about availability of data

All manuscripts must include a data availability statement. This statement should provide the following information, where applicable:

- Accession codes, unique identifiers, or web links for publicly available datasets
- A list of figures that have associated raw data
- A description of any restrictions on data availability

De-identified participant data will be made available on reasonable request two years after the date of publication. Requests should be directed to the corresponding author (mkosiborod@saint-lukes.org). Requestors will be required to sign a data access agreement to ensure the appropriate use of the study data.

Field-specific reporting

Please select the one below that is the best fit for your research. If you are not sure, read the appropriate sections before making your selection.

☒ Life sciences ☐ Behavioural & social sciences ☐ Ecological, evolutionary & environmental sciences

For a reference copy of the document with all sections, see nature.com/documents/nr-reporting-summary-flat.pdf

Life sciences study design

All studies must disclose on these points even when the disclosure is negative.

Sample size	For the primary endpoint a sample size of 145 for each group will achieve 82% power with α=0.05 to detect a 4.7 difference in mean KCCQ CS between dapagliflozin group and placebo group at 12 weeks. The assumptions for this calculation was derived from DEFINE-HF trial where the adjusted mean difference between dapagliflozin group and placebo group is 4.7 and the standard deviation is 13.7. Assuming a 10% loss to follow up, we arrive at a sample size of ~320 patients.
Data exclusions	Patients with no sufficient evaluable data for endpoint ascertainment during follow up were excluded. For example, 20 patients were excluded from the primary endpoint analysis because no followup KCCQ scores were available. This exclusion criterion was pre-specified in the Analysis Data Sets section of the SAP.
Replication	Independent validation was performed successfully by a different statistician for the primary and secondary analyses.
Randomization	Sharp Clinical Services' web-based IRT (ALEA v15.15851.201736 electronic randomization tool) was utilized to randomize participants to their double-blind treatment allocation. The randomization list was generated and maintained by Sharp Clinical Services and had a fixed block size of 8 stratification groups, based on three stratification questions (diabetes status, atrial fibrillation status fib and participation in the echo substudy). The randomization allocation sequence was implemented through sequentially numbered containers. Sites accessed the IRT through the web at the randomization visit and participants were assigned specific containers by the IRT.
Blinding	All the trial team investigators were blinded to treatment allocation throughout the study and during the data analysis. Participants remained blinded to treatment allocation throughout the duration of the study.

Reporting for specific materials, systems and methods

We require information from authors about some types of materials, experimental systems and methods used in many studies. Here, indicate whether each material, system or method listed is relevant to your study. If you are not sure if a list item applies to your research, read the appropriate section before selecting a response.

Materials & experimental systems

n/a	Involved in the study	
☒	☐	Antibodies
☒	☐	Eukaryotic cell lines
☒	☐	Palaeontology and archaeology
☒	☐	Animals and other organisms
☐	☒	Human research participants
☐	☒	Clinical data
☒	☐	Dual use research of concern

Methods

n/a	Involved in the study	
☒	☐	ChIP-seq
☒	☐	Flow cytometry
☒	☐	MRI-based neuroimaging

Human research participants

Policy information about studies involving human research participants

Population characteristics	Overall, median age was 70.0 (63.0, 77.0) years, 57% of patients were women, and 30% African American. The median duration of HF was 3.0 (1.0, 6.5) years and 56% had been hospitalized for HF at least once prior to study enrolment. Overall, 56% had T2D, and 53% had atrial fibrillation; median body mass index was 34.7 (interquartile range (IQR), 30.1-41.5). NYHA class II symptoms were present in 57%, with class III-IV symptoms in 42%. Baseline pharmacotherapy included mineralocorticoid antagonists (MRA) in 36%, ACE-I, ARB or ARNI in 62%, and loop diuretics in 88% of patients. Mean estimated glomerular filtration rate (eGFR) was 55 (41, 69)mL/min/1.73m2, median NT-proBNP 671.0 (IQR 355.0, 1297.0) pg/mL and median left ventricular ejection fraction (LVEF) was 60 (55, 65) percent.
Recruitment	Participants were recruited across 26 sites in the United States. Patients were identified from outpatient clinics and inpatient wards. All potentially eligible patients were invited to take part, thereby minimizing any potential self-selection bias.
Ethics oversight	Institutional review boards approved the study at all sites. Participating sites are listed in Supplementary Table 3 and Extended Figure 1.

Note that full information on the approval of the study protocol must also be provided in the manuscript.

Clinical data

Policy information about clinical studies

All manuscripts should comply with the ICMJE guidelines for publication of clinical research and a completed CONSORT checklist must be included with all submissions.

Clinical trial registration	This trial is registered with ClinicalTrials.gov, The PRESERVED-HF Trial, NCT 03030235
Study protocol	The study protocol is provided with the manuscript (Supplemental Note).
Data collection	Participants were recruited across 26 sites in the United States (listed in Supplementary Table 3 and Extended Figure 1) over an enrollment period of approximately 50 months. Patients were identified from outpatient clinics and inpatient wards.
Outcomes	All of the primary and secondary outcomes were prespecified in the Statistical Analysis Plan. All statistical analyses performed for all outcome measures were thus predefined.
