# Supplementary material for: The SGLT2 inhibitor dapagliflozin in heart failure with preserved ejection fraction: a multicenter randomized trial
Source: Nat Med. 2021 Oct 28;27(11):1954–60. doi: 10.1038/s41591-021-01536-x (PMC8604725; doi:10.1038/s41591-021-01536-x)
Supplement: Supplementary file 3 — PRESERVED-HF protocol. [file 41591_2021_1536_MOESM3_ESM.pdf]

[redacted]

PRESERVED-HF
Effects of Dapagliflozin on Biomarkers, Symptoms and Functional Status in Patients with PRESERVED Ejection Fraction Heart Failure

EFFECTS OF DAPAGLIFLOZIN ON BIOMARKERS, SYMPTOMS AND FUNCTIONAL STATUS IN PATIENTS WITH PRESERVED EJECTION FRACTION HEART FAILURE (PRESERVED-HF TRIAL)

[redacted]

Protocol Number: D1690C00053
Study Number: ESR-16-12141

Protocol Version Date: March 20, 2020

[redacted]

[redacted]

[redacted]

[redacted]

CONFIDENTIAL

PROTOCOL SYNOPSIS

A 12-week randomized, double-blind, placebo-controlled trial to evaluate the effects of once-daily dapagliflozin 10 mg on heart failure disease-specific health status (symptoms and physical limitations) in patients with chronic heart failure with preserved systolic function. An imaging substudy will also be conducted to explore the effects of dapagliflozin vs. placebo on various echocardiographic parameters.

Study Hypothesis

Treatment with dapagliflozin 10 mg daily for 12 weeks will improve health status (symptoms and physical limitations) as compared with placebo in patients with chronic heart failure with preserved systolic function.

Principal Investigator

[redacted]

Study Centers and Number of Patients Proposed

This study will be performed at up to 35 centers in the United States. Approximately 320 patients will be randomized over a target enrollment period of approximately 36 months.

Study Period		Phase of Development
Estimated date of first patient enrolled	March 31, 2017	IV (post marketing)
Estimated date of last patient completed	October 31, 2020	IV (post marketing)

Primary Objective

To evaluate the effects of dapagliflozin, as compared with placebo, on heart failure disease-specific health status (symptoms and physical limitations) in patients with chronic heart failure with preserved systolic function.

Target Population

Male and female patients with chronic heart failure with preserved systolic function.

Investigational Product, Dosage, and Mode of Administration

Dapagliflozin 10 mg administered orally once daily for 12 weeks, in addition to standard of care for chronic heart failure with preserved systolic function.

CONFIDENTIAL

Comparator, Dosage and Mode of Administration

Matching placebo administered orally once daily for 12 weeks, in addition to standard of care for chronic heart failure with preserved systolic function.

Study Duration

After activation of the first site, it is expected that enrollment will take approximately 26 months. After randomization, dapagliflozin or placebo will be administered for 12 weeks. Renal function will be evaluated 1 week after discontinuation of dapagliflozin or placebo.

Primary Outcome Variable

1. Change from baseline in heart failure related health status using the Kansas City Cardiomyopathy Questionnaire (KCCQ) clinical summary score at 12 weeks

.

Secondary Outcome Variables

1. Change from baseline in heart failure related health status using the Kansas City Cardiomyopathy Questionnaire (KCCQ) overall summary score at 12 weeks
2. Change from baseline in NTproBNP at 6 and 12 weeks
3. Change from baseline in BNP at 6 and 12 weeks
4. Change from baseline in 6 minute walk test at 12 weeks
5. Change from baseline in BNP at 6 and 12 weeks
6. Change from baseline in HbA1c over the treatment period (evaluated separately in patients with and without type 2 diabetes)
7. Proportion of patients with a ≥ 5pts increase in KCCQ clinical summary score and KCCQ overall summary score at 12 weeks
8. Proportion of patients with a ≥ 20% decrease in NTproBNP at 6 and 12 weeks
9. Proportion of patients with a ≥ 5pts increase in KCCQ and a ≥ 20% decrease in NTproBNP at 6 and 12 weeks
10. Change in weight at 6 and 12 weeks
11. Change in systolic blood pressure at 6 and 12 weeks

Exploratory Outcome Variables

1. Composite mean hierarchical-rank clinical score between dapagliflozin vs. placebo. All patients will receive a global rank endpoint based on time to death (tier 1) time to HF hospitalization or urgent HF visit (tier 2) or change in KCCQ clinical summary score from baseline to 12 weeks
2. Number of heart failure hospitalizations
3. Number of urgent heart failure visits

CONFIDENTIAL

4. Number of heart failure hospitalizations and urgent heart failure visits
5. Proportion of patients that progress to diabetes during the treatment period (within the subgroup of patients without diabetes at baseline only)
6. Change from baseline in average weekly loop diuretic dose (furosemide equivalent)
7. Change in NYHA Class at 6 and 12 weeks.
8. Change from baseline in left atrial volume index and other measures of left ventricular diastolic function

Safety Variables

1. All cause death
2. Cardiovascular death
3. Non-fatal myocardial infarction (MI)
4. Stroke
5. Acute kidney injury (defined as doubling of serum creatinine based on the modified RIFLE criteria)
6. Adverse events (AEs) and serious adverse events (SAEs). AEs of special interest will include DKA, volume depletion (defined as hypotension, syncope, orthostatic hypotension or dehydration), severe hypoglycemic events and lower limb amputations.

Statistical Methods

Baseline demographic and clinical data will be described between treatment and placebo study arms as mean ± standard deviation for continuous variables and compared using Student's T-test. Whereas discrete variables will be represented as a number and (%) and compared using the χ^2 or Fisher's exact test, as appropriate.

The time course of continuous variables will be presented using standard descriptive summary statistics calculated at each scheduled measuring time point and the last individual measuring time point. Moreover, standard descriptive summary statistics will be calculated for the change (absolute or percent) from baseline to each scheduled measuring time point after baseline and the last individual measuring time point.

Statistical significance will be defined using two-sided tests with α=0.05, unless otherwise specified. All statistical analyses will be performed by the [redacted] of Biostatistics using SAS version 9.4 (SAS Institute, Cary, North Carolina).

The primary endpoint of the study is to compare health status change with dapagliflozin versus placebo as measured by KCCQ clinical summary scores (KCCQ CS) at 12 weeks. This will be analyzed using ANCOVA adjusting for baseline KCCQ CS, gender, eGFR, Diabetes status, permanent atrial fibrillation status, and LVEF. Patient participation in the Echocardiography sub-study will not be

CONFIDENTIAL

controlled for because we don't hypothesize that participation in the Echocardiography sub-study is associated with the outcome (KCCQ CS).

For the primary endpoint a sample size of 145 for each group will achieve 82% power with α=0.05 to detect a 4.7 difference in mean KCCQ CS between dapagliflozin group and placebo group at 12 weeks. The assumptions for this calculation was derived from Define-HF study where the adjusted mean difference between dapagliflozin group and placebo group is 4.7 and the standard deviation is 13.7. **Assuming a 10% loss to follow up, we arrive at a sample size of ~320 patients.**

We plan to repeat the analysis within the subgroups of patients with and without diabetes. Assuming 60% of the patients do not have type 2 diabetes, the non-diabetes stratum is estimated to have ~174 patients (~87 each arm), which will achieve 80% power with α=0.05 to detect a 5.9 difference in mean KCCQ CS between dapagliflozin and placebo at 12 weeks; assuming 10% loss to follow up, we arrive at a sample size of ~192 patients within the no diabetes subgroup (~60% of the total sample). This will provide a large enough subgroup of patients with no diabetes to provide preliminary data on the effects of dapagliflozin vs. placebo in this important subgroup – in which the effects of SGLT2 inhibitors have not been well described. For the diabetes subgroup, a sample size of 58 for each arm will achieve 80% power with α=0.05 to detect a 7.2 difference in mean KCCQ CS between dapagliflozin and placebo at 12 weeks.; assuming 10% loss to follow up, we arrive at a sample size of ~128 patients within the diabetes subgroup (40% of the total sample). The above calculations demonstrate that the current overall study sample size is sufficient to examine the effects of dapagliflozin vs. placebo on the primary endpoint; and will provide sufficient numbers of patients within the key subgroups (diabetes, no diabetes) to examine whether important interactions exist in the effects of dapagliflozin vs. placebo by diabetes status.

Of note, participation of patients with permanent atrial fibrillation is allowed, but may be capped at no more than 33% (105 patients) of the entire cohort.

CONFIDENTIAL

Contents

Protocol Synopsis 2
1 Background and significance 12
1.1 Research Hypothesis 13
1.2 Rationale for conducting this study 13
1.3 Benefit/risk and ethical assessment 13
1.3.1 Risk Category 13
1.3.2 Potential Risks 14
1.3.3 Protection against Risks 15
1.3.4 Benefit to Patients 16
1.3.5 Informed Consent and Alternatives to Patients 16
1.3.6 Conclusion 16
2 Study Objective 16
2.1 Primary Outcome 16
2.2 Secondary Outcomes 17
2.3 Exploratory Outcomes 17
2.4 Safety Outcomes 17
3 Study Plan and Procedures 18
3.1 Study Design 18
3.2 Study Procedures 18
3.3 Definition of Active Treatment 20
3.4 Definition of Control Arm 20
3.5 Overall Study Duration 20
4 Study Population 21
4.1 Inclusion criteria 21
4.2 Exclusion criteria 22

CONFIDENTIAL

5 Study Conduct 24
5.1 Restrictions during the study 24
5.2 Patient enrollment and randomization 24
5.3 Procedures for randomization 24
5.4 Procedures for handling incorrectly enrolled or randomized patients 25
5.5 Blinding and procedures for unblinding the study 25
5.5.1 Methods for ensuring blinding 25
5.5.2 Methods for unblinding the study 26
5.6 Treatments 26
5.6.1 Identity of study medication 26
5.6.2 Doses and treatment regimens 26
5.6.3 Drug Dispensing Scheme 27
5.6.4 Duration of treatment 27
5.6.5 Labeling 27
5.6.6 Storage 27
5.7 Concomitant and post-study treatments 27
5.7.1 Recording of concomitant medication 27
5.8 Treatment Compliance 27
5.8.1 Accountability 28
5.9 Discontinuations of study medication 28
5.9.1 General discontinuation criteria 28
5.9.2 Study-specific discontinuation criteria 28
5.9.3 Procedures for permanent discontinuation of a patient from study medication 28
5.9.4 Patient agrees to undergo the Premature Treatment Discontinuation Visit and then continue in-person study visits 29

CONFIDENTIAL

5.9.5 Patient refuses to continue in-person study visits but agrees to undergo modified follow-up 29
5.9.6 Patient refuses any form of follow-up 29
5.9.7 Restart of study medication 29
5.9.8 Study Closure Visit 29
5.10 Withdrawal from study 29
5.10.1 Patients permanently discontinuing from study medication should be given conventional therapy, if applicable, and should always be asked to continue to attend protocol visits 30
5.11 Study committees 30
5.11.1 Executive Committee (EC) 30
5.11.2 Steering Committee 30
5.11.3 Clinical Endpoint Adjudication Committee (CEC) 30
5.11.4 Independent Data and Safety Monitoring Committee (IDSMC) 30
6 Collection of Study Variables 31
6.1 Recording of data 31
6.2 Data Collection at enrollment and follow-up 31
6.2.1 Screening Visit Procedures (Visit S) 32
6.2.2 Randomization Visit (Visit 1) 32
6.2.3 Visit 2, 3, 4 and 6 (phone visits) 33
6.2.4 Visit 5 and 7 (office visits) 33
6.2.5 Visit 8 (Study Closure Visit) 34
6.2.6 Premature Treatment Discontinuation Visit (PTDV) 34
6.3 Patient Monitoring During Study Visits 34
6.3.1 Physical examination 34
6.3.2 Phone Visits 35
6.4 Vital signs and weight 35

CONFIDENTIAL

6.4.1 Blood pressure and pulse 35
6.4.2 Orthostatic blood pressure 36
6.4.3 Supine BP and pulse 36
6.4.4 Standing BP and pulse 36
6.4.5 Weight 36
6.5 Six Minute Walk Test 36
6.6 Collection of Kansas City Cardiomyopathy Questionnaire (KCCQ) 37
6.7 Laboratory assessments 37
7 Biological Sampling Procedures 37
7.1 Volume of blood 37
7.2 Handling, storage and destruction of biological samples 38
8 Safety 38
8.1 Definition of adverse events (AE) 38
8.2 Definitions of serious adverse event (SAE) 38
8.2.1 Classification of Death 39
8.2.2 Universal classification of Myocardial Infarction (MI) 39
8.2.3 Definition of Stroke 39
8.2.4 Primary ischemic stroke 39
8.2.5 Primary hemorrhagic stroke 39
8.2.6 Unclassified stroke 39
8.2.7 Hospitalizations for heart failure 39
8.2.8 Acute Kidney Injury 39
8.2.9 Ketoacidosis (DKA) 39
8.2.10 Volume Depletion 40
8.3 Recording of adverse events 40
8.3.1 Collection of Adverse Events 40

CONFIDENTIAL

8.3.2 Follow-up of unresolved Adverse Events 40
8.3.3 Variables 41
8.3.4 Causality collection 41
8.3.5 Adverse Events based on signs and symptoms 42
8.3.6 Adverse Events based on examinations and tests 42
8.3.7 Hypoglycemic events 42
8.4 Reporting of serious adverse events 42
8.4.1 Reporting of serious adverse events to FDA [redacted] 43
9 Ethical and Regulatory Requirements 44
9.1 Ethical conduct of the study 44
9.2 Subject data protection 44
9.3 Ethics and regulatory review 44
9.4 Informed consent 44
9.5 Changes to the protocol and informed consent form 45
9.6 Audits and inspections 45
9.7 Posting of information on clinicaltrials.gov 45
10 Study Management by [redacted] 45
10.1 Pre-study activities 45
10.2 Training of study site personnel 46
10.3 Monitoring of the study 46
10.4 Source data 46
10.5 Study agreements 46
10.6 Archiving of study documents 46
10.7 Study timetable and end of study 46
11 Data Management by [redacted] 47
12 Statistical Methods and Sample Size Determination 47

CONFIDENTIAL

12.1 Description of analysis sets 47
12.1.1 Efficacy analysis set 47
12.1.2 Safety analysis set 47
12.2 Methods of statistical analyses 47
12.2.1 Primary Outcome 49
12.2.2 Secondary Outcomes 49
12.2.3 Exploratory Outcomes 49
12.2.4 Safety Outcomes 50
12.2.5 Analysis for safety 50
12.3 Determination of sample size 50
13 Important Medical Procedures to be Followed by the Investigator 50
13.1 Medical emergencies and [redacted] 50
13.2 Overdose 51
13.3 Pregnancy 51
14 Echocardiography substudy 51
15 Future Research 52
16 References 52
17 Appendix A: Heart Failure hospitalization/URGENT outpatient visit 55
18 Appendix B: Kansas City Cardiomyopathy Questionnaire (KCCQ) 57
19 Appendix C: Echocardiography Substudy 61
20 Appendix D: COVID-19 66
21 Appendix E: Principal investigator signature 67

CONFIDENTIAL

1 BACKGROUND AND SIGNIFICANCE

Heart failure with preserved ejection fraction (HFpEF), which currently represents approximately half of all HF cases, is a common clinical syndrome and a leading cause of morbidity and mortality.[4] Furthermore, the prevalence of HFpEF is increasing.[5] In spite of this rapidly rising economic and healthcare burden, no treatment has yet been shown to reduce morbidity and mortality in patients with HFpEF.

Diabetes adversely affects outcomes of all types of cardiovascular diseases.[6] In particular, diabetes is associated with a 70% to 80% increase in mortality and hospitalizations in patients with HFpEF.[7-9] Both diabetes and pre-diabetes are highly prevalent amongst the HFpEF population (diabetes is present in up to 40 percent, and pre-diabetes in up to 30% of these patients).[9-11] Lindman et al found that patients with HFpEF and diabetes had a more severe disease phenotype characterized by decreased exercise capacity, increased left ventricular hypertrophy, and increased circulating markers of vasoconstriction, oxidative stress, inflammation, and fibrosis.[9, 12] Patients with diabetes or pre-diabetes and concomitant HFpEF face a double challenge: not only are their outcomes particularly poor, with no currently available treatments for HFpEF proven to improve survival and/or reduce HF hospitalizations, but there are also no evidence-based options for diabetes management (or diabetes prevention), with none of the currently available glucose-lowering medications specifically tested for efficacy or safety in this high-risk group.[13]

The recently published EMPA-REG OUTCOME trial noted a 38% relative risk reduction of cardiovascular death with empagliflozin, a SGLT-2 inhibitor, versus placebo in patients with diabetes and established CVD.[3] Though the trial was predominantly of diabetic patients with coronary artery disease, the majority of the benefit appeared to be due to the reduction in hospitalizations for heart failure (a 35% relative risk reduction). The significant reductions in HF hospitalizations and death from HF are particularly intriguing since only a small minority (10%) of the patients in the trial had a HF diagnosis at baseline. Therefore, the majority of the HF benefit appears to be due to prevention of new HF. Although the etiology of hospitalizations for heart failure was not captured (HFpEF vs. HFrEF) it stands to reason that given a population of overweight, diabetic patients with a baseline 40% diuretic use, that undiagnosed HFpEF was likely highly prevalent.

While the mechanism of action for such a dramatic benefit in prevention of HF and cardiovascular mortality is not well understood, there are multiple theoretical benefits of SGLT2 inhibitors on diastolic heart function. There have been numerous animal and preclinical models showing that diabetic patients have increased markers of oxidative stress and poor endothelial function making them high risk for HFpEF.[14] We hypothesize that the majority of the benefit of SGLT-2 inhibitors in HFpEF is due to a reduction in oxidative stress, (improving diastolic function), improvement in endothelial function (thus decreasing vessel stiffness), anti-inflammatory effects and inhibitory effects on sympathetic nervous system (which could be one of the key mechanisms given the potential role of increased catecholamine levels in the pathophysiology of HFpEF).[15]Supporting this hypothesis are rat models where SGLT-2

CONFIDENTIAL

inhibition was shown to normalize endothelial function, reduce oxidative stress in aortic vessels, reverse a pro-inflammatory phenotype, improve AGE/RAGE signaling all pathways of potential importance to a reduction in arterial stiffness, and have an apparent protective effect on the myocardium in the setting of increased circulating epinephrine and norepinephrine levels.[15] Given the combination of EMPA-REG results and pre-clinical studies, it is highly likely that SGLT2 inhibition may have a significant clinical benefit in patients with HFpEF and diabetes or pre-diabetes – which could have a profound impact in a population that is in great need of novel treatments that improve outcomes.

Dapagliflozin, a sodium-glucose co-transporter 2 (SGLT2) inhibitor, is currently approved in the US for treatment of type 2 diabetes, and is being investigated for its potentially beneficial effects in patients with diabetes and HFrEF. Accordingly, we propose to perform a randomized clinical trial to evaluate the effects of dapagliflozin on disease-specific heart failure biomarkers, symptoms, health status, and quality of life, in patients with type 2 diabetes or no diabetes (many of whom will be expected to have pre-diabetes) and chronic heart failure with preserved ejection fraction.

1.1 Research Hypothesis

Treatment with dapagliflozin 10 mg daily for 12 weeks will improve health status (symptoms and physical limitations)_ in patients with chronic heart failure with preserved systolic function.

1.2 Rationale for conducting this study

This is a Phase IV study that will determine whether dapagliflozin provides a unique benefit to patients with chronic heart failure with preserved systolic function by improving patients' heart failure-related health status (symptoms and physical limitations).

1.3 Benefit/risk and ethical assessment

Dapagliflozin is approved for the treatment of type 2 diabetes and to reduce the risk of hospitalization for heart failure in adults with type 2 diabetes and established cardiovascular disease or multiple cardiovascular risk factors, therefore patients enrolled in the study that have type 2 diabetes will have an established indication for dapagliflozin therapy. Although dapagliflozin is currently not approved in patients without diabetes, when dapagliflozin is used either as monotherapy or in addition to metformin, it does not cause excess hypoglycemia as compared with placebo. No additional safety issues (beyond those observed with dapagliflozin in patients with type 2 diabetes) are anticipated in patients without diabetes treated with dapagliflozin. Accordingly, we consider the benefit/risk balance to patients enrolled in the study to be comparable to that encountered in the usual clinical practice, with no additional ethical concerns. Of note, several large cardiovascular outcome trials are currently evaluating various SGLT-2i as potential therapies for HF (including HFpEF), and include patients with and without diabetes.[19-21]

1.3.1 Risk Category

Considering dapagliflozin's mechanism of action, the previous clinical experience with dapagliflozin, the study's design features (including the inclusion, exclusion, and discontinuation criteria), and the planned safety procedures, participation in this study presents a minimal and thus acceptable risk to the individual patients that will be included.

CONFIDENTIAL

1.3.2 Potential Risks

The potential risks associated with dapagliflozin that have been identified based upon the mechanism of action, the preclinical results, and the clinical experience to date, as well as precautions included in the Phase III program to monitor and/or minimize these risks, are included in the dapagliflozin prescribing information.

In clinical Phase III studies, events suggestive of UTI were reported in a slightly higher proportion of dapagliflozin-treated patients than the placebo group. Increased urinary glucose excretion may also lead to an increased risk of developing genital infections. In Phase III studies, the proportions of patients treated with dapagliflozin who reported adverse events that were indicative of genital infection were higher than those seen for placebo.

In a pooled analysis of all phase 2b and 3 studies in the dapagliflozin development program there was an imbalance in the frequency of subjects who had a serious adverse event of breast cancer or bladder cancer. The significance of these findings is not clear at present; however a causal relationship with the use of dapagliflozin seems unlikely.

Overall there were no imbalances of liver function test parameters in Phase III studies. One subject on dapagliflozin 5 mg had a serious adverse event reported as drug-induced acute hepatitis and was later also diagnosed with probable autoimmune hepatitis.

Due to the diuretic effect of dapagliflozin, volume depletion (dehydration, hypovolemia and/or hypotension) is a potential concern. In the clinical program, from which subjects who in the judgment of investigator may be at risk of dehydration or volume depletion were excluded, very few serious events related to volume depletion were reported and they were equally distributed between dapagliflozin and placebo groups. In the limited experience in subjects with type 2 diabetes on concomitant loop diuretics, events related to volume depletion were more common in the dapagliflozin groups compared with the placebo group. Temporary interruption of dapagliflozin should be considered for subjects who develop volume depletion. In the recent analysis of patients with preexisting heart failure using pooled data from previous dapagliflozin studies, the rate of hypovolemic events was similar between dapagliflozin and placebo. Of note, all patients in this study will be required to have elevated BNP at baseline, which will further minimize the risk of hypovolemic adverse events.

The U.S. Food and Drug Administration (FDA) recently reported a warning for sodium-glucose cotransporter-2 (SGLT2) inhibitors which may lead to ketoacidosis, a serious condition where the body produces high levels of blood acids called ketones that may require hospitalization. At the time of this report there were 20 cases of acidosis reported as diabetic ketoacidosis (DKA), ketoacidosis, or ketosis in patients treated with SGLT2 inhibitors. DKA, a subset of ketoacidosis or ketosis in diabetic patients, is a type of acidosis that usually develops when insulin levels are too low or during prolonged fasting. DKA most commonly occurs in patients with type 1 diabetes and is usually accompanied by high blood sugar levels. The FDA reported cases were not typical for DKA because most of the patients had type 2 diabetes and their blood sugar levels, when reported, were only slightly increased compared to typical cases of DKA. Factors identified in some reports as having potentially triggered the ketoacidosis included major illness, reduced food and fluid intake, and reduced insulin dose. Although the risk of euglycemic DKA is estimated to be very low in this study (given the short duration of treatment, and the fact that patients with type 2 diabetes are at lower risk for DKA than type 1 diabetes, and patient without diabetes are likely not at risk for DKA) all patients will be provided with home urine ketone testing kits, and patients will be monitored for symptoms of DKA during in-person visits and study-related phone calls. Patients will be instructed to

CONFIDENTIAL

self-test for urine ketones and be directed to the closest emergency department if the urine ketone test is more than mildly positive. The instances of DKA (if any) will be closely monitored as SAE of special interest by the study investigators, as well as the Independent Data and Safety Monitoring Committee. In addition, all patients in this study will continue taking glucose-lowering medications (other than open-label SGLT-2 inhibitors) as background therapy. These drugs are widely used anti-hyperglycemic treatments and will be prescribed according to the approved label in patients with known diabetes.

Dapagliflozin prescribing information states that the drug should not be started in patients with eGFR <60. Per the FDA submission documents the main reason dapagliflozin clinical studies were not designed to include patients with eGFR between 30-45 was because "glycemic efficacy was not expected in the absence of adequate renal function."[4] The focus of the PRESERVED Trial is not glycemic control, but rather dapagliflozin effects on heart failure endpoints. There are many reasons to believe that SGLT2 inhibitors may have beneficial effects on heart failure and renal endpoints regardless of baseline eGFR, including in patients with eGFR between 30-60. In fact, a recent secondary analysis from the EMPA-REG Outcome large scale clinical trial of empagliflozin showed dramatic reduction in cardiovascular mortality and hospitalizations for heart failure in patients with Type 2 diabetes; patients with eGFR as low as 30 were allowed to be included in that trial.[4,5] Furthermore, in the same trial (EMPA-REG Outcome) marked benefit was observed with empagliflozin vs. placebo for clinically important renal endpoints, including doubling of creatinine and progression to ESRD.[3,6] These effects were observed consistently across the range of baseline eGFR. Meta analyses of completed dapagliflozin trials suggest a similar effect of dapagliflozin on cardiovascular and renal parameters.[7]

The US FDA also issued a more recent safety alert in regards to SGLT2-inihibtors and potential risk for acute kidney injury. The FDA letter mentions 101 cases of acute kidney injury with SGLT-2 inhibitors, of which only 28 involved dapagliflozin. While the exact denominator is unknown to calculate an incidence rate, these 28 open label cases were reported during a time period when over 300,000 prescriptions were filled for dapagliflozin in the US. Further, these spontaneous reports do not prove a cause-and effect link between SGLT2-inhibitors and renal events. The renal safety (and in fact nephroprotective effects) of these agents have been demonstrated in clinical trials as stated above. The safety meta analysis of dapagliflozin trials also showed no evidence for increase in acute kidney injury or acute renal failure events.[16]

We plan to monitor renal function carefully in the PRESERVED-HF study, and doubling of serum creatinine is a safety variable that is being carefully ascertained; furthermore, all patients in PRESERVED-HF Trial are volume overloaded at baseline given the requirement for significantly elevated NTproBNP, and therefore should be at low risk for hypovolemic events. The Independent Safety and Data Monitoring Committee will also be reviewing safety data continuously.

Thus, the benefits and risks associated with the background medication and comparator treatment are well established and presented in their respective approved prescribing information. No study procedure will put patients at a risk significantly beyond those ordinarily encountered during the performance of routine medical examinations or routine tests.

1.3.3 Protection against Risks

This study has been designed with appropriate measures in place so as to monitor and minimize any of the potential health risks to participating patients. This includes careful monitoring of patients'

CONFIDENTIAL

vital signs and laboratory values, and the temporary and if necessary permanent discontinuation of investigational product in individual patients in whom a potential health risk or a laboratory abnormality of clinical concern has been identified. Further, in order to ensure the safety of all patients participating in this study, an Independent Data and Safety Monitoring Committee (IDSMC) will be formed that will continuously review safety data, including the incidence of serious adverse events (SAEs), and conduct assessments to ensure the ongoing safety of study patients. The IDSMC responsibilities, authorities, and procedures are documented in an IDSMC charter. The personnel involved in the clinical study at [redacted] will remain blinded to these analyses and will have no knowledge of the results presented to the IDSMC.

1.3.4 Benefit to Patients

All patients will continue taking their active background therapy; although a direct benefit from randomized treatment cannot be assured as one half of patients will receive placebo, those with type 2 diabetes or prediabetes randomized to dapagliflozin may obtain better glucose control. In this study, the dose of dapagliflozin 10 mg once daily was chosen to provide efficacy in improving heart failure symptoms and biomarkers, as well as reducing HbA1c while mitigating the potential for AEs, based on previous clinical experience. In addition, among patients randomized to active drug, dapagliflozin is expected to help maintain better glucose control among those with type 2 diabetes or prediabetes, decrease body weight (or prevent weight gain) as well as help lower blood pressure especially in patients with elevated baseline blood pressure (which is very common in patients with HFpEF). All patients are also expected to receive some benefit in the form of increased medical care/attention when participating in study procedures, which includes at least 5 clinic visits with at least 5 physical examinations over the 13-week study.

1.3.5 Informed Consent and Alternatives to Patients

All prospective participants will be informed of the possible risks and benefits associated with this study, and their consent will be received prior to performing any study-related activity. When a prospective participant elects to not participate in the study or to withdraw from the study, other medications are available to treat their heart failure, and the patient will not be disadvantaged in any way.

1.3.6 Conclusion

Considering the pre-clinical and clinical experience with dapagliflozin and the precautions included in the study protocol, participation in this study presents a minimal and thus acceptable risk to patients who meet the inclusion/exclusion criteria and consent to take part in the study.

For additional details on benefits and risk, please see the dapagliflozin prescribing information.

2 STUDY OBJECTIVE

To evaluate the impact of dapagliflozin, as compared with placebo, on heart failure disease-specific biomarkers, symptoms, health status, and quality of life in patients with chronic heart failure with preserved systolic function.

2.1 <u>Primary Outcome</u>

To evaluate the effects of dapagliflozin, as compared with placebo, on heart failure disease-specific health status (symptoms and physical limitations) in patients with chronic heart failure with preserved systolic function.

CONFIDENTIAL

2.2 Secondary Outcomes

1. Change from baseline in heart failure related health status using the Kansas City Cardiomyopathy Questionnaire (KCCQ) overall summary score at 12 weeks
2. Change from baseline in NTproBNP at 6 and 12 weeks
3. Change from baseline in BNP at 6 and 12 weeks
4. Change from baseline in 6 minute walk test at 12 weeks
5. Change from baseline in BNP at 6 and 12 weeks
6. Change from baseline in HbA1c over the treatment period
7. Proportion of patients with a ≥ 5pts increase in KCCQ clinical summary score and KCCQ overall summary score at 12 weeks
8. Proportion of patients with a ≥ 20% decrease in NTproBNP at 6 and 12 weeks
9. Proportion of patients with a ≥ 5pts increase in KCCQ and a ≥ 20% decrease in NTproBNP at 6 and 12 weeks
10. Change in weight at 6 and 12 weeks
11. Change in systolic blood pressure at 6 and 12 weeks

2.3 Exploratory Outcomes

1. Composite mean hierarchical-rank clinical score between dapagliflozin vs. placebo. All patients will receive a global rank endpoint based on time to death (tier 1) time to HF hospitalization or urgent HF visit (tier 2) or change KCCQ clinical summary score from baseline to 12 weeks
2. Number of heart failure hospitalizations
3. Number of urgent heart failure visits
4. Number of heart failure hospitalizations and urgent heart failure visits
5. Proportion of patients that progress to diabetes during the treatment period (within the subgroup of patients without diabetes at baseline only)
6. Change from baseline in average weekly loop diuretic dose (furosemide equivalent)
7. Change in NYHA Class at 6 and 12 weeks.
8.
9. Change from baseline in left atrial volume index and other measures of left ventricular diastolic function

2.4 Safety Outcomes

1. All cause death

CONFIDENTIAL

2. Cardiovascular death
3. Non-fatal myocardial infarction (MI)
4. Stroke
5. Acute kidney injury (defined as doubling of serum creatinine based on modified RIFLE criteria)
6. Adverse events (AEs) and serious adverse events (SAEs). AEs of special interest will include DKA, volume depletion (defined as hypotension, syncope, orthostatic hypotension or dehydration), severe hypoglycemic events and lower limb amputations.

3 STUDY PLAN AND PROCEDURES

3.1 Study Design

Randomized, double-blind, placebo-controlled trial. The control group will receive placebo administered orally once daily for 12 weeks plus standard of care. The treatment group will receive dapagliflozin 10 mg administered orally once daily for 12 weeks plus standard of care. A follow-up visit at week 13 will be performed to evaluate markers of renal function.

3.2 Study Procedures

At the screening visit, participants will undergo a physical exam (including vital signs and weight assessment), a laboratory panel, including HbA1c, BNP, NTproBNP, and a renal panel will be performed to determine study eligibility (Table 1: Study Plan). At the randomization visit, participants will undergo a physical exam (including vital signs and weight assessment), laboratory testing, including HbA1c, BNP, NTproBNP and a renal panel, complete the KCCQ, and perform a 6 minute walk test. Treatment or placebo will be administered for 12 weeks, with follow-up visits at 6 and 12 weeks during which a physical exam (including vital signs and weight assessment), labs, KCCQ (at the 12 week visit only), a 6-minute walk test (at the 12 week visit only), will be performed and AEs/SAEs will be recorded. On days 2 and 10, as well as weeks 4 and 9 participants will be contacted by phone to evaluate for AEs/SAEs, and encourage compliance with the study medication. One week after treatment ends, renal function will be evaluated at a follow-up office visit.

Table 1: Study Plan

			12 –week double-blind treatment period						
	Screening	Randomization							
**Visit**	**S[J]**	**1**	**2**	**3**	**4**	**5**	**6**	**7**	**8**
**Week[g)]**	**-2**	**0**	**2d**	**10d**	**4**	**6**	**9**	**12** PTDV	**13**

CONFIDENTIAL

Office Visit	X	X				X		X	X
Phone Visit[f]			X	X	X		X		
Informed consent	X								
Physical Exam [a]	X	X				X		X	X
Vital signs (BP, pulse)	X	X				X		X	X
Orthostatic BP, pulse	X	X				X		X	X
NYHA Class	X	X				X		X	X
Weight	X	X				X		X	X
Height	X								
Body Mass Index (BMI)	X								
Waist circumference	X	X				X		X	X
Hip circumference	X								
Medical History	X								
Concomitant medication	X	X	X	X	X	X	X	X	X
Laboratory assessments	X[b]	X [c,k]				X [c]		X [c,k]	X [d]
Urine pregnancy test[e]	X	X				X		X	X
Urine albumin/ creatinine ratio test[i]	X	X				X		X	X
Echocardiogram [h,l]		X						X	
6 min walk test		X						X	
KCCQ		X						X	
AEs		X	X	X	X	X	X	X	X
SAEs		X	X	X	X	X	X	X	X
Hospitalizations		X	X	X	X	X	X	X	X
ER Visits		X	X	X	X	X	X	X	X
Urgent outpatient heart failure visits		X	X	X	X	X	X	X	X
Dispense urine ketone strips		X							
Dispense study medication		X							
Return/redispense study medication						X		X	
Study medication accountability						X		X	

[a] **Physical Exam includes**: complete physical examination consisting of general appearance, head, eyes, ears, nose, throat, neck, cardiovascular system, lungs, abdomen, lymph nodes, extremities, neurological

CONFIDENTIAL

system, skin, musculoskeletal system, height (screening only), weight, pulse, blood pressure, monitoring for volume depletion, and assessment for ketoacidosis (difficulty breathing, nausea, vomiting, abdominal pain, confusion, and unusual fatigue or sleepiness).

b) Screening laboratory assessment includes HbA1c, BNP, NTproBNP and renal panel.

c) Randomization, Week 6 and Week 12 laboratory assessment includes HbA1c, Fasting Glucose, BNP, NTproBNP, CBC, renal panel, and collection of samples for future potential biomarker analyses (Randomization and Week 12 only).

d) Week 13 laboratory assessment includes only renal panel.

e) Only for women with childbearing potential.

f) Phone visits include recording any AE or SAE, self-monitoring of weight and blood glucose (patients with established type 2 diabetes only), and encouraging compliance with study medication.

g) Visit Windows: There may be up to 2 weeks between the screening and randomization visits. Week 6, 12 and 13 clinic visits have a +/- 2-day visit window. Phone Visits have a +/-2-day visit window.

h) An echocardiogram will be completed in those subjects participating in echo sub study at Visit 1 (Week 0 Randomization) and Visit 7 (Week 12)

i) The urine specimens collected at 0, 6 and 12 weeks will only be checking urine albumin and urine creatinine. It will not be a standard urinalysis, and any clinical suspicion of urinary tract infection will be left to the local investigator or patient's primary care physician to order a proper screening test to evaluate.

j) If a patient is a screen failure at the initial screening visit, they may be rescreened on three additional occasions at the discretion of the investigator. At the rescreening visit, the patient should be treated like a new patient and assigned a new subject number and all screening visit procedures should be completed, including obtaining informed consent.

k) Subjects should be resting in the supine position for at least 5 minutes prior to collection of blood samples for biomarkers at Visit 1 (Week 0 Randomization) and Visit 7 (Week 12).

l) For all patients, their most recent clinically available echocardiogram prior to study enrollment will be submitted using the WebPAX system, if feasible. This will be a historical Echo which is a part of the patient record and is independent to any study-related Echo image acquisition.

**COVID-19: For modified study visit options applicable during the time period of the COVID-19 emergency, please see appendix D.

3.3 Definition of Active Treatment

Dapagliflozin 10 mg daily + Standard of Care for heart failure

3.4 Definition of Control Arm

Matching Placebo + Standard of Care for heart failure

3.5 Overall Study Duration

Subjects will participate for a total of 13 weeks. It is estimated that the total study duration will be 26 months.

CONFIDENTIAL

4 STUDY POPULATION

Voluntary participation will be sought from patients with chronic heart failure with preserved systolic function at outpatient general cardiology and specialized heart failure clinics. Informed consent will be obtained from potentially eligible participants prior to initiating screening visit procedures.

It is expected that approximately 60%% (174 patients) of the study cohort will have pre-diabetes or normal glucose metabolism (no diabetes), and therefore, the proportion of patients with established Type 2 diabetes may be capped at 45% (~146 patients). The proportion of patients with permanent atrial fibrillation may be capped at 33% of the study cohort (105 patients).

Randomization will be stratified by diabetes status (diabetes vs. no diabetes), permanent atrial fibrillation status (Yes vs. No), and by patients' participation in the echocardiography sub-study.

4.1 Inclusion criteria

1. Age > 18 and < 120 at the screening visit
2. Symptoms of dyspnea (NYHA class II-IV) without evidence of a non-cardiac or ischemic explanation for dyspnea
3. Ejection fraction (EF) ≥ 45% as determined on imaging study within 24 months of enrolment with no change in clinical status suggesting potential for deterioration in systolic function
4. Elevated NT-proBNP (≥ 225 pg/ml) or BNP (≥ 75 pg/ml) [ǂ]
5. Stable medical therapy for heart failure for 15 days as defined by:
    i. No addition or removal of ACE, angiotensin receptor blockers (ARBs), valsartan/sacubitril, beta-blockers, calcium channel blockers (CCBs) or aldosterone antagonists
    ii. No substantial change in dosage (100% or greater increase or decrease from baseline dose) of ACE, ARBs, beta-blockers, CCBs or aldosterone antagonists
6. On a diuretic ≥15 days prior to screening visit and a stable diuretic therapy for 7 days
7. At least one of the following:
    i. Hospitalization for decompensated HF in the last 12 months
    ii. Acute treatment for HF with intravenous loop diuretic or hemofiltration in the last 12 months
    iii. Mean pulmonary capillary wedge pressure ≥15 mmHg or LV end diastolic pressure (LVEDP) ≥15 mmHg documented during catheterization at rest, or pulmonary capillary wedge pressure or LVEDP ≥25 mmHg documented during catheterization with exercise.
    iv. Structural heart disease evidenced by at least one of the following echo findings (any local measurement made within the 24 months prior to screening visit):

1) left atrial (LA) enlargement defined by at least one of the following: LA width ≥3.8cm or LA length ≥5.0 cm **or** LA area ≥20 cm2 **or** LA volume ≥55 mL **or** LA volume index ≥29 mL/m2

2) OR left ventricular hypertrophy (LVH) defined by septal thickness or posterior wall thickness ≥1.1 cm.

4.2 Exclusion criteria

1. Decompensated heart failure (hospitalization for heart failure within 7 days prior to screening)
2. History of type 1 diabetes
3. History of diabetic ketoacidosis
4. Estimated glomerular filtration rate (eGFR) < 20 at the screening visit by modified MDRD equation GFR (mL/min/1.73 m2) = 175 x (Scr) $^{-1.154}$ x (Age)$^{-0\ 203}$ x (0.742 if female) x (1.210 if African American)
5. Admission for an acute coronary syndrome (ST-elevation MI, non-ST-elevation MI, or unstable angina), percutaneous coronary intervention, or cardiac surgery within 30 days prior to the screening visit.
6. Admission for cardiac resynchronization therapy (CRT) within 90 days prior to the screening visit.
7. Planned cardiovascular revascularization (percutaneous intervention or surgical) or major cardiac surgery (coronary artery bypass grafting, valve replacement, ventricular assist device, cardiac transplantation, or any other surgery requiring thoracotomy, or transcatheter aortic valve replacement) or CRT within the 90 days after the screening visit.
8. Participation in any interventional clinical trial (with an investigational drug or device) that is not an observational registry within 15 days of the screening visit.
9. History of hypersensitivity to dapagliflozin
10. For women of child-bearing potential: Current or planned pregnancy or currently lactating.

    Women of childbearing potential are defined as any female who has experienced menarche and who is NOT permanently sterile or postmenopausal. Post menopausal is defined as 12 consecutive months with no menses without an alternative medical cause. Women of child-bearing potential, who are sexually active, must agree to use a medically-accepted method of birth control for the duration of the study. Acceptable birth control methods include: (1) surgical sterilization (such as a hysterectomy or bilateral tubal ligation), (2) progesterone hormonal contraceptives (birth control pills or implants), (3) barrier methods (such as a condom or diaphragm) used with a spermicide, or (4) an intrauterine device (IUD). Women of child-bearing potential will have a urine pregnancy test at every clinic visit and it must be negative to continue study participation.

11. Life expectancy <1 year at the screening visit

CONFIDENTIAL

12. Patients who are volume depleted based upon physical examination at the time of the screening or randomization visit
13. BNP <75 pg/mL and NTproBNP<225 pg/mL at the screening visit [£]
14. Patients currently being treated with any SGLT-2 inhibitor (dapagliflozin, canagliflozin, empagliflozin, ertugliflozin) or having received treatment with any SGLT-2 inhibitor within the 12 weeks prior to the screening visit.
15. Average supine systolic BP <100 mmHg at the screening or randomization visit
16. Current history of bladder cancer
17. Donation of blood or bone marrow 12 weeks prior to the screening visit and no planned donations during the study period
18. Heart failure due to restrictive/infiltrative cardiomyopathy, active myocarditis, constrictive pericarditis, severe stenotic valve disease, and HOCM (hypertrophic obstructive cardiomyopathy).
19. Heart failure due to severe aortic or mitral regurgitation
20. Severe COPD thought to be a primary contributor to dyspnea
21. Isolated right heart failure due to pulmonary disease
22. Active and significant ischemia thought to be a primary contributor to dyspnea
23. Documentation of previous EF < 45%, under stable conditions, within the past 36 months
24. Complex congenital heart disease
25. Uncontrolled hypertension, defined as systolic blood pressure ≥200 mmHg during the screening visit (average value of three blood pressure measurements obtained in supine position)
26. Any other condition that in the judgment of the investigator would jeopardize the patient's participation in the study or that may interfere with the interpretation of study data or if the patient is considered unlikely to comply with study procedures, restrictions and requirements
27. Bariatric surgery within the past 6 months or planned bariatric surgery within the study time course.
28. CardioMems device implantation within previous 4 weeks or planned CardioMems implantation during study period
29. For echo substudy only: patients with ventricular paced rhythm or left bundle branch block on the most recent clinically available 12-lead electrocardiogram.
30. For echo substudy only: permanent atrial fibrillation

[ŧ] For patients with permanent atrial fibrillation inclusion thresholds will be BNP ≥ 100 pg/mL or NTproBNP ≥ 375 pg/mL

£For patients with permanent atrial fibrillation exclusion thresholds will be BNP<100 pg/mL and NTproBNP<375pg/mL

5 STUDY CONDUCT

5.1 Restrictions during the study

Patients should be fasting from all food and beverages (except water) at least 6 hours before blood samples are taken for laboratory analysis at a clinic visit with the exception of the screening visit. It is preferred but not required that patients be fasting at the screening visit. Patients should not use alcohol for 24 hrs or use tobacco for 12 hrs prior to testing at a clinic visit. Patients with established diabetes or prediabetes should not take any glucose-lowering medication when they are fasting. On the day of a clinic visit, investigational product and other concomitant medications will be taken in the morning, after completion of certain required study procedures. For patients with established type 2 diabetes on basal insulin, it is recommended they only take ½ of their basal dose the evening before they are planning to fast for an office visit. Patients shall not be allowed to use any prescribed SGLT-2 inhibitors (dapagliflozin, canagliflozin, empagliflozin), other than the investigational product, at any time during the study. Patients shall not be allowed to donate blood or bone marrow at any time during the study. Patients shall not be allowed to participate in any other interventional clinical trial (with a drug or device) for the duration of the study.

5.2 Patient enrollment and randomization

The Principal Investigator or delegate will:

1. Obtain signed informed consent from the potential patient before any study specific procedures are performed
2. Determine patient eligibility
3. Assign potential patients a sequential enrollment number in the form of Site ID and enrollment number i.e.: XXX-XXX
4. Assign enrolled patient a unique randomization code using Sharp Clinical Interactive Voice and Web Response Systems (IVR/IWR).

If a patient withdraws from participation in the study, then their enrollment number cannot be reused. Patients can only be randomized into the study once.

5.3 Procedures for randomization

Sharp Clinical Services will provide a state-of-the-art Clinical Interactive Voice and Web Response Systems (IVR/IWR). Sharp Clinical IVR/IWR is an innovative value-based product for Subject enrollment, randomization, capturing clinical data, drug shipments, and managing drug supply.

Sharp Clinical IVR/IWR Solutions are 21 CFR Part 11 compliant, user-friendly, and provide value to all users with big reductions in study start up times. The IVR/IWR System ensures data integrity, accelerates clinical site initiations, and can provide real-time metrics for subjects, sites and study inventory for approved users.

Solutions and Services include:

- 24/7 Operation
- Site Administration and Tracking
- Study Drug Distribution and Resupply Management

Training and User-Materials:

During system development, the Sharp PM creates a study-specific user manual and Quick Reference Guide for the IVR/IWR System. Site and client users are trained at investigator meetings or scheduled web-based training sessions conducted by the Sharp PM.

5.4 Procedures for handling incorrectly enrolled or randomized patients

Patients who fail to meet the inclusion/exclusion criteria should not, under any circumstances, be enrolled or randomized. There can be no exceptions to this rule.

The following steps should be taken in the event that a patient, who does not meet inclusion/exclusion criteria, is found to have been inadvertently randomized in the study:

1. The investigator should inform the [redacted] study team physician immediately. Ensuring patient safety must always be the number one priority.
2. Study treatment must be discontinued in all cases where continued treatment is deemed to pose a safety risk to the patient. After a discussion between the study team physician and investigator, a decision may be reached that the patient should discontinue study medication. The rationale for discontinuing study medication must be clearly documented. The patient should remain in the study for follow up in accordance with defined study procedures including follow-up on endpoints through the end of the study consistent with the FAS principle.
3. In those cases where continuation of study therapy is judged not to present a concern related to safety and disease management, the rationale for continuing study therapy must be clearly documented. The patient should continue follow up in accordance with defined study procedures.

5.5 Blinding and procedures for unblinding the study

5.5.1 Methods for ensuring blinding

The treatment allocation in this study will be double blind. Dapagliflozin (10 mg) tablets and matching dapagliflozin placebo tablets will be provided, identical in appearance and with the same number, size, and packaging of tablets. Each bottle will be labeled with a unique bottle ID number that will be used to assign the treatment to the patient but will not indicate treatment allocation to the investigator.

No member of the extended study team at [redacted], the EC, the CEC, or personnel at investigational centers will have access to the randomization scheme during the conduct of the study, with the exception of the Sharp Clinical Services, and the Biostatistics department at [redacted].

The IDSMC will have access to the individual treatment codes and will be able to merge these with the collected study data while the study is ongoing. The IDSMC will review safety data on a periodic basis, including the incidence of SAEs, and conduct safety assessments to ensure the ongoing safety of study patients. The IDSMC responsibilities, authorities, and procedures will be documented in a IDSMC charter. The personnel involved in the clinical study at [redacted] will remain blinded to these analyses and will have no knowledge of the results presented to the IDSMC.

5.5.2 Methods for unblinding the study

Individual treatment codes, indicating the treatment allocation for each randomized patient, will be available to the investigator(s) or pharmacists from the Sharp Clinical IVR/IWR system. Routines for this will be described in the Sharp Clinical IVR/IWR system user manual that will be provided to each study site.

The treatment code should not be broken except in medical emergencies when the appropriate management of the patient requires knowledge of the treatment. The [redacted] physician ([redacted]) should be consulted whenever possible prior to the investigator breaking the blind. The investigator documents and reports the action to [redacted], without revealing the treatment given to the patient to the [redacted] staff. The number of individuals at the study site who become aware of the treatment status should be kept to an absolute minimum including keeping the patient blinded if possible. Treatment with study medication should be continued if considered appropriate.

[redacted] retains the right to break the code for SAEs that are unexpected and are suspected to be causally related to a study drug and that potentially require expedited reporting to regulatory authorities. Treatment codes will not be broken for the planned analyses of data until all decisions on the availability of the data from each individual patient have been made and documented.

5.6 Treatments

5.6.1 Identity of study medication

Table 2 Identity of study medication

Study Medication	Dosage form and strength	Manufacturer
Dapagliflozin	Biconvex, diamond shape, green tablet 10 mg (Size: 11 mm)	[redacted]
Matching placebo for dapagliflozin 10 mg	Biconvex, diamond shape, green tablet (Size: 11 mm)	[redacted]

5.6.2 Doses and treatment regimens

At the randomization visit eligible patients will be randomly assigned to 1 of 2 treatments:

- Dapagliflozin 10 mg, administered orally once daily for the 12 weeks.
- Matching placebo for dapagliflozin 10 mg, administered orally once daily for the 12 weeks.

The investigational product dapagliflozin and matching placebo will be taken orally. The investigational product should be taken once daily in the morning and at approximately the same time of the day during the study period. Nevertheless prior to each office visit, patients with established type 2 diabetes or prediabetes should be instructed not to take any glucose-lowering

CONFIDENTIAL

medication in the morning and to abstain from all food and beverages for 6 hours; however, drinking water is allowed. On the day of an office visit, investigational product and other concomitant medications will be taken in the morning, after completion of certain required study procedures.

5.6.3 Drug Dispensing Scheme

At randomization, three (3) bottles of dapagliflozin 10 mg or matching placebo will be dispensed, with each bottle containing 35 tablets. A total of 105 tablets will be dispensed.

5.6.4 Duration of treatment

The control group will receive placebo administered orally once daily for 12 weeks plus standard of care. The treatment group will receive dapagliflozin 10 mg administered orally once daily for 12 weeks plus standard of care. Subjects will participate for a total of 13 weeks.

5.6.5 Labeling

Labels will be prepared in accordance with Good Manufacturing Practice (GMP) and local regulatory guidelines. The labels will fulfill GMP Annex 13 requirements for labeling. The label will include at least the following information:

- Name of sponsor: [redacted]
- Study drug(s) dosage form, route of administration, and quantity of dosage units
- Study code
- Enrollment code (will be added by the investigator when investigational product is dispensed)
- Kit ID
- Directions for use (For oral use)
- Storage conditions
- "for clinical trial use only"
- "keep out of reach of children"

5.6.6 Storage

All study drugs should be kept in a secure place under appropriate storage conditions and in the original container. The study medication label and dapagliflozin prescribing information specify appropriate storage.

5.7 Concomitant and post-study treatments

5.7.1 Recording of concomitant medication

Detailed recording of all concomitant medications will be made at screening, randomization, and all subsequent visits. It will include all medication changes, but glucose lowering and heart failure medications in particular.

5.8 Treatment Compliance

The administration of study medication should be recorded. All stops of study medication prescribed by the investigator should be recorded. In addition, any non-prescribed temporary stops (>1 week) of study medication should be recorded.

Missed doses of dapagliflozin or placebo blinded study medication should not be taken. If a dose is missed, the next regularly scheduled dose should be taken and should not be doubled.

CONFIDENTIAL

5.8.1 Accountability

The study medication provided for this study will be used only as directed in the study protocol. The study personnel will account for all study medication dispensed to and returned from the patient. Patients will be asked to bring all unused study medication and empty packages to the site at each office visit. The investigator or delegate will record the number of returned tablets and make an assessment regarding patient treatment compliance. Any patient found to be noncompliant would be counseled on the importance of taking their study medication as prescribed.

Any study medication deliberately or accidentally destroyed must be recorded. Any discrepancy between dispensed and returned study medication should be explained.

The investigator will retain the returned medication until the termination of the Clinical Study. Then the investigator will return any unused medication to Sharp Clinical Services for destruction of all unused study medication [redacted] is responsible for confirming the investigator or delegate has recorded the quantities of returned and unused tablets at a patient level before medication is returned to Sharp Clinical Services.

5.9 <u>Discontinuations of study medication</u>

Patients should be discontinued from study medication in the following situations:

5.9.1 General discontinuation criteria

1. Patient decision. The patient is at any time free to discontinue treatment, without prejudice to further treatment.
2. Adverse Events, i.e., any clinical AE, laboratory abnormality or concurrent illness which, in the opinion of the investigator, indicates that continued participation in the study is not in the best interest of the patient.
3. Severe non-compliance to protocol as judged by the investigator and/or [redacted].
4. Risk to patients as judged by the investigator.
5. Incorrectly enrolled patients.
6. Patient lost to follow-up.

5.9.2 Study-specific discontinuation criteria

1. Doubling of serum creatinine above the baseline value confirmed by a repeated measurement within one week.
2. Recurrent severe hypoglycemic events, defined as ≥2 severe events (a severe hypoglycemic event is defined as symptomatic events requiring external assistance due to severe impairment in consciousness or behavior with a capillary or plasma glucose value <54 mg/dL or blood glucose <54 mg/dL). This definition should be applied after possible contributing factors (eg, excessive physical activity, dietary and medication factors) have been excluded or addressed by the investigator.
3. Pregnancy confirmed by a positive pregnancy test or otherwise verified.

5.9.3 Procedures for permanent discontinuation of a patient from study medication

Patients permanently discontinuing study medication should be given conventional therapy, if applicable, and should continue routine care visits with their primary physician.

A patient that decides to discontinue study medication will always be asked about the reason(s) for their desire to discontinue study medication and the presence of AEs (if any). These data will be ascertained and documented by the investigator. AEs will be followed up and the patient should return all study medications.

It is essential to collect as much data as possible for all patients throughout the study and especially all potential endpoint events. Discontinuation from study medication is not the same as complete withdrawal from the study (withdrawal of consent), which has a direct impact on the potential validity of all study data, and should be avoided whenever possible.

5.9.4 Patient agrees to undergo the Premature Treatment Discontinuation Visit and then continue in-person study visits

The patient agrees to undergo the Premature Treatment Discontinuation Visit (PTDV) and then continue in-person study visits according to plan. This is the preferred option and patients who discontinue study medication will always be asked if they agree to this approach. If agreed, as above, the patient will undergo their PTDV at the next scheduled office visit after the study medication is stopped. The patient will continue attending subsequent study visits according to schedule (Table 1).

5.9.5 Patient refuses to continue in-person study visits but agrees to undergo modified follow-up

If the patient refuses to continue in-person study visits, but agrees to undergo modified follow up, the in-person PTDV visit should be performed as soon as possible after the study medication is stopped. All subsequent visits until the end of study date will be done as modified follow-ups (eg, regular telephone contacts, a contact at study closure, or other means) in order to ascertain whether any endpoints or safety events had occurred. Such a patient has not withdrawn his/her consent or withdrawn from the study.

5.9.6 Patient refuses any form of follow-up

If the patient refuses any form of follow-up, he/she officially withdraws from the study and withdraws consent. This decision must be documented. At the end of the study, vital status on all such patients will be collected from publicly available sources, in accordance with local regulations.

5.9.7 Restart of study medication

Whenever possible, and at every study visit, restart of randomized study medication should be encouraged, even if a PTDV was previously completed.

5.9.8 Study Closure Visit

All randomized patients should return for their study closure visit (visit 8) as soon as possible, but no later than 1 week after the previously scheduled visit 7.

If a patient is unable to attend the study closure visit in person, telephone contact should be made to ascertain endpoint and AE information. At the study closure visit, physicians caring for the patient will decide which medication the patient should receive as part of his/her ongoing clinical care.

5.10 <u>Withdrawal from study</u>

Patients are at any time free to withdraw from the study (i.e., discontinue study medication permanently and withdraw from visit assessments), without prejudice to further treatment (withdrawal of consent). Withdrawal of consent from the study must be ascertained and documented by the investigator. Such patients will always be asked about the reason(s) and the presence of any AEs. The reason for permanent discontinuation of treatment with the study medication and the date of the last intake of the study medication must be documented.

CONFIDENTIAL

5.10.1 Patients permanently discontinuing from study medication should be given conventional therapy, if applicable, and should always be asked to continue to attend protocol visits

If the patient denies any additional protocol follow-up and officially withdraws consent from the study one of the alternatives a) to c) should be followed:

- At the time of discontinuation of treatment and withdrawal of consent from continued assessment the patient should, if possible, undergo the PTDV. The patient should return all study medication
- If the patient does not agree to this option (which must be documented), a modified PTDV (eg, a telephone contact) should be arranged. The approach taken should be documented. The patient should return all study medication
- If the patient does not agree to a) or b) this must be documented in the patient's medical record. The patient should return all study medication.

To ensure validity of study data, it is very important to collect as much data as possible throughout the study and especially vital status (dead or alive) at the SCV. The investigator or delegate will therefore attempt to collect information on all patients' vital status from publicly available sources at the SCV, in accordance with local regulations, even if informed consent has been withdrawn completely.

5.11 Study committees

5.11.1 Executive Committee (EC)

The EC will be responsible for the overall design, including the development of the protocol and any protocol amendments, supervision, interpretation and reporting (presentations at international congresses and publications in peer reviewed journals) of the study. The EC will make recommendations to [redacted] with regard to early stopping or modifications of the study based on the information received from the IDSMC. The EC will be comprised of the overall study PI (EC Chair) and designated academic leaders with expertise in the fields of heart failure and cardiometabolic disease. The precise responsibilities and procedures applicable for the EC will be detailed in a separate EC charter.

5.11.2 Steering Committee

A steering committee will be formed and composed of PIs from each participating site. A publication plan will be developed with input from this committee.

5.11.3 Clinical Endpoint Adjudication Committee (CEC)

An independent CEC will be appointed and will adjudicate all heart failure hospitalizations, urgent heart failure visits and major cardiovascular events (cardiovascular death, myocardial infarction, stroke). The committee members will not have access to individual treatment codes for any patient or clinical efficacy and safety event. The precise responsibilities and procedures applicable for the CEC will be detailed in a separate CEC charter.

5.11.4 Independent Data and Safety Monitoring Committee (IDSMC)

An independent DSMC will be appointed.

CONFIDENTIAL

The IDSMC will be responsible for safeguarding the interests of the patients in the study by assessing the safety of the intervention during the study, and for reviewing the overall conduct of the clinical study. The IDSMC will have access to the individual treatment codes and will be able to merge these with the collected study data while the study is ongoing.

The EC and [redacted] will not be made aware of the treatment codes until after clean file and database lock are declared. Similarly, all summary output reviewed at each IDSMC meeting will be held in confidence by the IDSMC members until the end of the study when clean file and database lock are declared.

The IDSMC charter will be prepared to detail precise roles and responsibilities and procedures to ensure maintenance of the blinding and integrity of the study in the review of accumulating data and interactions with the Study PI (EC Chair).

The IDSMC may elect to request an Interim efficacy and/or futility analysis at its discretion in consultation with the overall study PI (EC Chair). If this option is elected, the IDSMC charter will be amended accordingly.

6 COLLECTION OF STUDY VARIABLES

6.1 Recording of data

The REDCap Web Based Data Capture (WBDC) system will be used for data collection and query handling. The site Principal Investigator will ensure that data are recorded in the electronic Case Report Forms (eCRF) and will ensure the accuracy, completeness, and timeliness of the data recorded and of the provision of answers to data queries according to the Clinical Study Agreement (CSA).

Data will be entered in the eCRF using the REDCap Web Based Data Capture (WBDC) system by trained personnel at the study site. When data have been entered, reviewed, edited, and source data verification has been performed, as appropriate, by an [redacted] representative, the data will be frozen to prevent further editing. The site Principal Investigator will be notified to sign the eCRF electronically. A copy of the eCRF data will be archived at the study site.

6.2 Data Collection at enrollment and follow-up

TABLE 3 Laboratory variables

Visit	S	1	2	3	4	5	6	7	8
**Week**	**-2**	**0**	**2d***	**10d***	**4**	**6**	**9**	**12**	**13**
HbA1c	X	X				X		X	
Glucose (included in the renal panel)		X				X		X	

BNP	X	X	X	X	
NTproBNP	X	X	X	X	
Urine albumin creatinine Ratio	X	X	X	X	X
Urine Pregnancy Test	X	X	X	X	X
CBC		X	X	X	
Renal Panel**	X	X	X	X	X
Biomarkers***		X		X	

*days after randomization

** The renal panel includes albumin, BUN/Creatinine Ratio (calculated), calcium, carbon dioxide, chloride, creatinine, estimated glomerular filtration rate (calculated), glucose, phosphate (as phosphorus), potassium, sodium, urea nitrogen

*** serum, plasma, buffy coat, RNA will be stored for future potential biomarker analyses

6.2.1 Screening Visit Procedures (Visit S)

- Informed consent
- Blood sampling for laboratory assessments
- Physical exam
- Vital Signs (seated pulse and BP and orthostatic pulse and BP)
- Weight
- Height
- Waist circumference
- Hip circumference
- Calculate BMI (Formula: weight (kg) / [height (m)]^2)
- Urine pregnancy test (only applicable for women of childbearing potential)
- Urine albumin/creatinine ratio
- Medical history
- Concomitant medications
- Eligibility criteria

6.2.2 Randomization Visit (Visit 1)

Patients that fulfill the eligibility criteria will undergo randomization procedures.

- Eligibility criteria
- Physical exam
- Vital Signs (seated pulse and BP and orthostatic pulse and BP)
- Weight
- Waist circumference

CONFIDENTIAL

- Fasting blood sampling for laboratory assessments and biomarkers
- Urine pregnancy test (only for women of childbearing potential)
- Urine albumin/creatinine ratio
- Dispensed study medication
- Dispense Urine Ketone Strips
- Concomitant medications
- 6 minute walk test
- KCCQ questionnaire
- Adverse events
- Serious adverse events
- Hospitalizations, ER visits, urgent outpatient visits for heart failure
- Contact IVR/IWR for randomization
- Dispense study medication
- Echocardiogram for those participating in echo substudy

6.2.3 Visit 2, 3, 4 and 6 (phone visits)

- Concomitant medications
- Adverse events
- Serious adverse events
- Hospitalizations, ER visits, urgent outpatient visits for heart failure
- Self-monitored weight
- Self-monitored Blood Glucose (for patients with type 2 diabetes only)
- Volume depletion monitoring
- Ketoacidosis monitoring
- Encourage compliance with study medication

6.2.4 Visit 5 and 7 (office visits)

- Physical exam
- Vital Signs (seated pulse and BP and orthostatic pulse and BP)
- Weight
- Waist circumference
- Fasting blood sampling for laboratory assessments and biomarkers (biomarkers to be collected at visit 7 only)
- Urine pregnancy test (only for women of childbearing potential)
- Urine albumin/creatinine ratio
- Concomitant medications
- 6 minute walk test (at Visit 7 only)
- KCCQ questionnaire (at Visit 7 only)
- Concomitant medications
- Adverse events
- Serious adverse events
- Hospitalizations, ER visits, urgent outpatient visits for heart failure
- Volume depletion monitoring
- Ketoacidosis monitoring
- Study medication return (redispense at Visit 5) and accountability
- Contact IVR/IWR at Visit 7 for treatment completion

CONFIDENTIAL

- Participants in echo substudy will undergo echocardiogram at the 12 week visit.

6.2.5 Visit 8 (Study Closure Visit)

- Physical exam
- Vital Signs (seated pulse and BP and orthostatic pulse and BP)
- Weight
- Waist circumference
- Fasting blood sampling for laboratory assessments (only for renal panel).
- Urine pregnancy test (only for women of childbearing potential)
- Urine albumin/creatinine ratio
- Concomitant medications
- Adverse events
- Serious adverse events
- Hospitalizations, ER visits, urgent outpatient visits for heart failure
- Volume depletion monitoring
- Ketoacidosis monitoring

6.2.6 Premature Treatment Discontinuation Visit (PTDV)

- Performed at next on-site visit or as soon as possible (see section 5.9)
- Physical exam
- Vital Signs (seated pulse and BP and orthostatic pulse and BP)
- Weight
- Waist circumference
- Fasting blood sampling for laboratory assessments
- Urine pregnancy test (only applicable for women of childbearing potential)
- Urine albumin/creatinine ratio
- Concomitant medications
- 6 minute walk test
- KCCQ questionnaire
- Concomitant medications Adverse events
- Serious adverse events
- Hospitalizations, ER visits, urgent outpatient visits for heart failure
- Volume status monitoring
- Ketoacidosis monitoring
- Study medication return and accountability
- Contact IVR/IWR for treatment discontinuation
- Participants in echo substudy will undergo echocardiogram at PTDV.

6.3 <u>Patient Monitoring During Study Visits</u>

6.3.1 Physical examination

A physical examination should be done according to schedule shown in Study Plan (Table 1).

- A complete physical examination should include general appearance, head, eyes, ears, nose, throat, neck, cardiovascular system, lungs, abdomen, lymph nodes, extremities, neurological system, skin, and musculoskeletal system. Collection of pulse and blood

CONFIDENTIAL

pressure should also be collected as described in section 6.4. The patient should always be evaluated for the presence of edema and other signs of volume overload (jugular venous distention, rales, ascites, etc).

- Evaluation of volume depletion, including orthostatic vital signs and other physical exam findings consistent with dehydration
- Evaluation for the presence of ketoacidosis, in patients experiencing signs or symptoms of ketoacidosis, such as tachypnea or hyperventilation, anorexia, abdominal pain, nausea, vomiting, lethargy, or mental status changes; administer appropriate testing for ketoacidosis and direct patients to the emergency department if ketoacidosis is confirmed.
- For patients with type 2 diabetes, and if HbA1c ≤ 7.0% and receiving insulin at baseline, it is recommended to reduce total daily insulin dose by 20%. For patients with type 2 diabetes and if HbA1c ≤ 7.0% and receiving sulfonylurea at baseline, it is recommended to reduce total daily sulfonylurea dose by 50%, or discontinue sulfonylureas in patients receiving the minimal dose of sulfonylurea at baseline.
- Baseline data are collected at Visit 1 and any new or aggravated findings discovered on subsequent physical examinations should be recorded as AE if clinically relevant.
- It is recommended that self monitoring of blood glucose values is performed by the patients with established type 2 diabetes and reviewed during the study visits
- Review of self-monitoring of weight, with adjustments in loop diuretic dose if appropriate for optimization of volume status
- Review of SMBG values and glucose lowering medications for patients with established type 2 diabetes. Although investigators are encouraged not to routinely titrate glucose-lowering medications during the study in patients with type 2 diabetes, such adjustments should be considered for optimization of glucose control if considered necessary from a patient safety standpoint due to a significant hyper- or hypoglycemia.

6.3.2 Phone Visits

Phone visits should be done according to the schedule shown in Study Plan (Table 1). Evaluate possible AE and SAEs, medication usage, self-monitoring of weight and blood glucose (in patients with established type 2 diabetes).

Upon review of self-monitoring of weight and self-monitoring glucose (in patients with established type 2 diabetes) consider adjustments in loop diuretics and glucose-lowering medications for optimization of glucose control (in patients with established type 2 diabetes) and volume status if considered necessary from patient safety standpoint.

6.4 <u>Vital signs and weight</u>

6.4.1 Blood pressure and pulse

Blood pressure and pulse measurement will be taken after the patient has been sitting and resting for at least 5 minutes and before blood samples are taken. Blood pressure (BP) and pulse should be measured three times with at least 1 minute between each measurement. BP readings should be taken while the patient is in a comfortable seated position with the arm supported at the level of the heart. All readings should be recorded. The average of the three BP readings will be used for study analyses. At screening, the seated BP will be recorded three times in both the

CONFIDENTIAL

left and the right arms. All three measurements should be made in one arm before transferring the cuff to the other arm. The arm with the highest average seated systolic BP readings will be the one used for all subsequent readings. If there is a contraindication for measuring blood pressure in an arm, then no measurements should be taken in that arm.

Ideally, all blood pressure and pulse measurements should be taken with the same automated or manual blood pressure device, at the same time of day, and by the same personnel at each visit. Patients with persistent atrial fibrillation should have all of their blood pressure and pulse measured with a manual cuff.

6.4.2 Orthostatic blood pressure

At visits where orthostatic BP and pulse are collected, supine and standing measurements should be made after the seated BP and pulse measurements have been made, using the same arm that was used for the seated BP measurements. All readings should be recorded. Ideally, blood pressure should be measured with the same machine, at the same time of day, and by the same personnel at each visit.

6.4.3 Supine BP and pulse

The supine BP and pulse must be measured prior to the standing BP and pulse. After the patient rests in the supine position for at least 5 minutes, supine BP and pulse will be determined from three replicate measurements obtained at least 1 minute apart. All three readings must be recorded. For study analyses, the average of the three BP and pulse readings will be used.

6.4.4 Standing BP and pulse

After the supine BP and pulse measurements are obtained, the patient will stand for 2 to 3 minutes. After this time, the BP will be measured with the arm supported at the level of the heart. Standing BP and pulse will be determined from three replicate measurements obtained at least 1 minute apart. All 3 readings must be recorded. For study analyses, the average of the three BP and pulse readings will be used.

If a new occurrence of previously absent orthostatic hypotension is demonstrated, it should be recorded as AE. The investigator may consider reducing concomitant anti- hypertensive medication to alleviate signs and symptoms of orthostatic hypotension.

6.4.5 Weight

The patient's weight will be recorded in kilogram (kg) to one decimal place, with light clothing and no shoes. All readings should be recorded as accurately as possible and the same calibrated scale should be used for all assessments for a given patient. Body weight assessments should preferably be conducted in the morning after an overnight fast and after emptying the bladder.

6.5 <u>Six Minute Walk Test</u>

The 6MWT is a practical simple test that requires a 100-ft hallway but no exercise equipment or advanced training for technicians. Walking is an activity performed daily by all but the most severely impaired patients. This test measures the distance that a patient can quickly walk on a flat, hard surface in a period of 6 minutes (the 6MWD). It evaluates the global and integrated responses of all the systems involved during exercise, including the pulmonary and cardiovascular systems, systemic circulation, peripheral circulation, blood, neuromuscular units, and muscle metabolism. It does not provide specific information on the function of each of the different organs and systems involved in exercise or the mechanism of exercise limitation, as is possible with maximal cardiopulmonary

CONFIDENTIAL

exercise testing. The self-paced 6MWT assesses the submaximal level of functional capacity. Most patients do not achieve maximal exercise capacity during the 6MWT; instead, they choose their own intensity of exercise and are allowed to stop and rest during the test. However, because most activities of daily living are performed at submaximal levels of exertion, the 6MWD may better reflect the functional exercise level for daily physical activities.[17]

6.6 Collection of Kansas City Cardiomyopathy Questionnaire (KCCQ)

The KCCQ (see appendix B) is a disease-specific health status instrument composed of 23 items that quantify the domains of physical limitation, symptoms, self-efficacy, social limitation, and quality of life limitation from heart failure. The overall summary score and all domains have been independently demonstrated to be valid, reliable, and responsive to clinical change. Scores range from 0 to 100. For the KCCQ overall summary score, a small but clinically meaningful change is considered to be ≥ 5 points.[18] The patients will fill in PRO (KCCQ) paper form under the supervision of the site staff.

6.7 Laboratory assessments

Blood and urine samples for determination of clinical chemistry, hematology, urinalysis and biomarker testing will be taken at the times indicated in the Study Plan (see Table 1). Any additional laboratory safety samples taken at the investigator's discretion will be analyzed locally.

It is recommended that patients do not have their baseline diuretics changed based upon naturietic peptide levels, and that the patient's primary cardiologist be blinded to the BNP and NTproBNP data collected throughout the trial. There have been varying results with trials of NTproBNP guided clinical management of heart failure. Two randomized clinical trials have shown no clear benefit.[24-25] Given the uncertainty of benefit, neither serial collection nor treatment based upon NTproBNP levels are currently endorsed by ACC/AHA guidelines.[26] For these reasons, it is recommended that heart failure medication changes not be made based on naturietic peptide levels. If a patient is clinically symptomatic or endorses signs or symptoms of volume overload, clinical judgment should be used and diuretic adjustments made as warranted.

7 BIOLOGICAL SAMPLING PROCEDURES

7.1 Volume of blood

The total volume of blood that will be drawn from each patient for this study is listed in Table 4 below. The collection of additional samples is performed locally at the discretion of the investigator and recorded in the eCRF as appropriate, thus requiring additional sample volumes.

Table 4 Volume of blood to be drawn from each patient

Assessment	Sample volume (mL)	Number of Samples	Total Volume (mL)
HbA1c	4 mL whole blood	4	16 mL
Glucose	2 mL whole blood	3	6 mL
BNP	2 mL whole blood	4	8 mL
NTproBNP	2 mL whole blood	4	8 mL
CBC	4 mL whole blood	3	12 mL

CONFIDENTIAL

Renal Panel/Uric Acid	4 mL whole blood	5	20 mL
Serum, plasma, buffy coat, biobanking	40 mL whole blood	2	80 mL
Total			150 mL

7.2 Handling, storage and destruction of biological samples

Blood and urine samples will be processed by local staff for shipment to the central laboratory (Quest Diagnostics). All samples should be taken by adequately trained study personnel and handled in accordance with instructions in the central laboratory manual. Up to date reference ranges will be provided during the study and laboratory results will be compared to the laboratory standard normal ranges and flagged if they are outside the normal range. The Investigator should make an assessment of any clinically significant abnormalities in the laboratory reports. The laboratory reports should be signed, dated and retained at the study site as source data for laboratory variables.

The clinical chemistry, hematology, and urinalysis samples will be disposed after analyses.

8 SAFETY

8.1 Definition of adverse events (AE)

An AE is the development of an undesirable medical condition or the deterioration of a pre-existing medical condition following or during exposure to a pharmaceutical product, whether or not considered causally related to the product. An undesirable medical condition can be symptoms (eg, nausea, chest pain), signs (e.g., tachycardia, enlarged liver) or the abnormal results of an investigation (e.g., laboratory findings, electrocardiogram). In clinical studies, an AE can include an undesirable medical condition occurring at any time, including run-in or washout periods, even if no study treatment has been administered. The term AE is used to include both serious and non-serious AEs.

8.2 Definitions of serious adverse event (SAE)

An SAE is an AE occurring during any study phase (i.e., run-in, treatment, follow-up), that fulfills one or more of the following criteria:

- Results in death
- Is immediately life-threatening
- Requires in-patient hospitalization or prolongation of existing hospitalization
- Results in persistent or significant disability or incapacity
- Is a congenital abnormality or birth defect
- Is an important medical event that may jeopardize the patient or may require medical intervention to prevent one of the outcomes listed above.

Any event of cancer, drug dependency/abuse, laboratory abnormalities fulfilling the Hy's law definition or overdose (defined as the accidental or intentional ingestion of any dose of the

CONFIDENTIAL

investigational product that is considered both excessive and medically important) should be reported as an SAE using the most relevant SAE criteria, as judged by the Investigator.

8.2.1 Classification of Death

Deaths will be sub-classified by CV and non-CV primary cause. CV death includes sudden cardiac death, death due to acute MI, death due to heart failure, death due to a cerebrovascular event, death due to other CV causes (e.g., pulmonary embolism, aortic disease, CV intervention), and deaths for which there was no clearly documented non-CV cause (presumed CV death).

Additionally, deaths will be sub-classified by coronary heart diseases death (CHD death) and non-CHD death. CHD death includes Sudden Cardiac Death, Death due to Acute MI, and the subset of Death due to other CV Causes that are secondary to a coronary revascularization procedure.

8.2.2 Universal classification of Myocardial Infarction (MI)

The Third Universal MI definition[19] will be used as study specific MI criteria.

8.2.3 Definition of Stroke

Stroke is defined as an acute episode of neurologic dysfunction attributed to a central nervous system vascular cause. Stroke should be documented by imaging (eg, CT scan or magnetic resonance imaging [MRI] scan). Evidence obtained from autopsy can also confirm the diagnosis. Stroke will be sub classified, when possible, as either:

8.2.4 Primary ischemic stroke

Primary ischemic stroke is defined as an acute episode of focal brain, spinal, or retinal dysfunction caused by an infarction of central nervous system tissue and documented by imaging. A primary ischemic stroke may also undergo hemorrhagic transformation (i.e., no evidence of hemorrhage on an initial imaging study, but appearance on a subsequent scan).

8.2.5 Primary hemorrhagic stroke

Primary hemorrhagic stroke is defined as an acute episode of focal or global brain, spinal, or retinal dysfunction caused by non-traumatic intraparenchymal, intraventricular, or subarachnoid hemorrhage as documented by neuroimaging or autopsy. Microhemorrhages (<10 mm) evident only on MRI are not considered to be a hemorrhagic stroke. Subdural and epidural bleeding will be considered intracranial hemorrhage, but not strokes.

8.2.6 Unclassified stroke

Stroke with unknown etiology will be classified as unclassified stroke if the type of stroke could not be determined by imaging or other means.

8.2.7 Hospitalizations for heart failure

In the event that a patient is hospitalized for heart failure over the course of the study, source documents will be obtained to adjudicate events. See appendix A for detailed information.

8.2.8 Acute Kidney Injury

Acute kidney injury is defined as a doubling of creatinine, consistent with the modified RIFLE criteria for stage 2 acute kidney injury.

8.2.9 Ketoacidosis (DKA)

Be aware that postmarketing cases show a possible association between sodium-glucose cotransporter-2 (SGLT2) inhibitor use and the development of a high anion gap metabolic acidosis accompanied by elevation in urine or serum ketones, frequently in the setting of only mildly elevated glucose levels (euglycemic DKA). Investigators are strongly encouraged to

CONFIDENTIAL

instruct patients and caregivers about the signs and symptoms of ketoacidosis, such as tachypnea or hyperventilation, anorexia, abdominal pain, nausea, vomiting, lethargy, or mental status changes; evaluate for the presence of ketoacidosis in patients experiencing such signs or symptoms – using provided urine ketone testing kits; discontinue study medication and advice patients to go to the nearest emergency department if ketoacidosis is confirmed; and take appropriate measures to correct the ketoacidosis and to monitor glucose levels. Investigators are also strongly encouraged to avoid concomitant risk factors potentially predisposing to DKA, including carbohydrate-restricted diets and marked reductions in insulin dose (in patients with established type 2 diabetes) during the study among patients receiving insulin at baseline (above and beyond reductions in insulin dose (for patients with established type 2 diabetes) specified in the study protocol). Advise patients to alert you and seek medical attention immediately if they experience symptoms consistent with DKA such as: nausea, vomiting, abdominal pain, confusion, change in breathing pattern and unusual fatigue or sleepiness.

8.2.10 Volume Depletion

Dapagliflozin has a modest diuretic effect. The risk of volume depletion is enhanced when two diuretics are used in combination and in patients that otherwise are at risk for volume depletion. Therefore, caution should be exercised when administering to patients at risk for volume depletion due to co-existing conditions or concomitant medications, such as loop diuretics. These patients should be carefully monitored for volume status, electrolytes, and renal function, and encouraged to self-monitor weight during the study.

CONFIDENTIAL

8.3 Recording of adverse events

8.3.1 Collection of Adverse Events

AEs and SAEs (including hospitalizations for heart failure) will be recorded from Screening throughout the treatment period and including the follow-up period (Visit 8).

All AEs/SAEs are to be recorded by the site. SAEs, DAEs and AEs of Special Interest will be captured in the e-CRF. Non-serious AEs will not be captured in the e-CRF.

SAEs are defined in section 8.2.

A drug adverse event (DAE) is an adverse event which leads to premature and permanent discontinuation of study medication.

AEs of Special Interest will include DKA, volume depletion (defined as hypotension, syncope, orthostatic hypotension or dehydration), severe hypoglycemic events and lower limb amputations.

Information about all urgent outpatient heart failure visits will also be recorded by the site and captured in the e-CRF.

8.3.2 Follow-up of unresolved Adverse Events

Any AEs that are unresolved at the patient's last visit in the study are followed up by the investigator for as long as medically indicated. [redacted] retains the right to request additional information for any patient with ongoing AE(s)/SAE(s) at the end of the study, if judged necessary. The requirement to follow-up is not intended to delay database lock or

production of the clinical study report. Both these activities should proceed as planned with ongoing AEs if necessary.

Any follow-up of ongoing SAEs after database lock will be reported to [redacted], who will notify the appropriate regulatory authorities of [redacted] and study drug manufacturer.

8.3.3 Variables

The following variables will be collected for each AE;

- AE (verbatim)
- Date when the AE started and stopped
- Maximum intensity
- Whether the AE is serious or not
- Investigator causality rating against the Investigational Product (yes or no)
- Action taken with regard to investigational product
- Outcome

In addition, the following variables will be collected for SAEs:

- Date AE met criteria for serious AE
- Date Investigator became aware of serious AE
- AE is serious due to
- Date of hospitalization
- Date of discharge
- Probable cause of death
- Date of death
- Autopsy performed
- Causality assessment in relation to Study procedure(s)
- Description of AE

Maximum intensity will be graded according to the following rating scale:

1. mild (awareness of sign or symptom, but easily tolerated)
2. moderate (discomfort sufficient to cause interference with normal activities)
3. severe (incapacitating, with inability to perform normal activities)

It is important to distinguish between serious and severe AEs. Severity is a measure of intensity. An AE of severe intensity need not necessarily be considered serious. For example, nausea that persists for several hours may be considered severe nausea, but not a SAE. On the other hand, a stroke that results in only a limited degree of disability may be considered a mild stroke but would be a SAE.

8.3.4 Causality collection

The Investigator will assess causal relationship between Investigational Product and each Adverse Event, and answer 'yes' or 'no' to the question 'Do you consider that there is a reasonable possibility that the event may have been caused by the investigational product?'

CONFIDENTIAL

For SAEs causal relationship will also be assessed for other medication and study procedures. Note that for SAEs that could be associated with any study procedure the causal relationship is implied as 'yes'.

8.3.5 Adverse Events based on signs and symptoms

All AEs spontaneously reported by the patient or reported in response to the open question from the study personnel: 'Have you had any health problems since the previous visit/you were last asked?', or revealed by observation will be collected. When collecting AEs, the recording of diagnoses is preferred (when possible) to recording of a list of signs and symptoms. However, if a diagnosis is known and there are other signs or symptoms that are not generally part of the diagnosis, the diagnosis and each sign or symptom will be recorded separately.

8.3.6 Adverse Events based on examinations and tests

The results from protocol mandated laboratory test and vital signs will be summarized in the clinical study report. Deterioration as compared to baseline in protocol-mandated laboratory values and vital signs will only be reported as AEs if they are clinically significant, fulfill any of the SAE criteria or are the reason for discontinuation of treatment with the investigational product, or require the patient to receive specific corrective therapy.

If deterioration in a laboratory value/vital sign is associated with clinical signs and symptoms, the sign/symptom will be reported as an AE and the associated laboratory result/vital sign will be considered as additional information. Wherever possible the reporting investigator uses the clinical, rather than the laboratory term (e.g., anemia versus low hemoglobin value). In the absence of clinical signs or symptoms, clinically relevant deteriorations in non-mandated measurements will be reported as AE(s).

Any new or aggravated clinically relevant abnormal medical finding at a physical examination as compared with the baseline assessment will be reported as an AE.

8.3.7 Hypoglycemic events

Information about all hypoglycemic events should be collected. Only severe hypoglycemic episodes should be captured in the eCRF. A severe hypoglycemic event is defined as a symptomatic event requiring external assistance due to severe impairment in consciousness or behavior with a capillary or plasma glucose value <54 mg/dL.

8.4 Reporting of serious adverse events

All SAEs have to be reported to [redacted], whether or not considered causally related to the investigational product. The site investigator is responsible for informing their local IRB as per local requirements.

Investigators and other center personnel must inform appropriate [redacted] representatives via the web based data capture (WBDC) system of any SAE that occurs in the course of the study within 1 day (i.e., immediately but no later than the end of the next business day) of when he or she becomes aware of it. Follow-up information on SAEs must also be reported by the Investigator within the same time frame.

An automated email alert will be sent to the designated [redacted] representative, when the page with SAE information is saved in WBDC system by the Investigators or other site personnel. If the WBDC system is not available, then the Investigator or other study site personnel reports by fax an SAE to the appropriate [redacted] representative. A paper back- up SAE report is

used for this purpose. The same reporting time frames still apply. The investigator is responsible for completing the eCRF as soon as the system becomes available again.

The [redacted] representative will work with the Investigator to compile all the necessary information and ensure that all the necessary information is provided to [redacted] within one calendar day of initial receipt for fatal and life threatening events and within five calendar days of initial receipt for all other SAEs.

8.4.1 Reporting of serious adverse events to FDA [redacted]

The Sponsor [redacted]) will inform the FDA, via a MedWatch/AdEERs form, of any serious or unexpected adverse events that occur in accordance with the reporting obligations of 21 CFR 312.32, and will concurrently forward all such reports to [redacted] A copy of the MedWatch/AdEERs report must be faxed to [redacted] at the time the event is reported to the FDA. It is the responsibility of the investigator to compile all necessary information and ensure that the FDA receives a report according to the FDA reporting requirement timelines and to ensure that these reports are also submitted to [redacted] at the same time.

When reporting to [redacted] a cover page should accompany the MedWatch/AdEERs form indicating the following:

Externally Sponsored Research (ESR)

The investigator IND number assigned by the FDA

The investigator's name and address

The trial name/title and [redacted] ESR reference number

Investigative site must also indicate, either in the SAE report or the cover page, the causality of events in relation to all study medications and if the SAE is related to disease progression, as determined by the principal investigator.

Send SAE report and accompanying cover page by way of fax [redacted]

Serious adverse events that do not require expedited reporting to the FDA need to be reported to [redacted] preferably using the MedDRA coding language for serious adverse events.

In the case of blinded trials, [redacted] may request that the Sponsor either provide a copy of the randomization code/ code break information or unblind those SAEs which require expedited reporting.

All SAEs will be reported to [redacted], whether or not considered causally related to the investigational product. All SAEs will be documented.

CONFIDENTIAL

9 ETHICAL AND REGULATORY REQUIREMENTS

9.1 Ethical conduct of the study

The study will be performed in accordance with ethical principles that have their origin in the Declaration of Helsinki and are consistent with ICH/Good Clinical Practice (GCP), applicable regulatory requirements and the [redacted] policy on Bioethics.

9.2 Subject data protection

The Informed Consent Form will incorporate (or, in some cases, be accompanied by a separate document incorporating) wording that complies with relevant data protection and privacy legislation.

9.3 Ethics and regulatory review

An Ethics Committee/Institutional Review Board (IRB) should approve the final study protocol, including the final version of the Informed Consent Form and any other written information and/or materials to be provided to the patient. The investigator/Head of the study site will ensure the distribution of these documents to the applicable Ethics Committee/IRB, and to the study site staff.

The opinion of the Ethics Committee should be received in writing. The investigator should submit a notification of direction/determination as well as a copy of the IRB written approval to [redacted] before enrolment of any patient into the study.

The Ethics Committee/IRB should approve all advertising used to recruit patients for the study.

[redacted] should approve any modifications to the Informed Consent Form that are needed to meet local requirements.

If required by local regulations, the protocol should be re-approved by the Ethics Committee annually.

[redacted] will provide Regulatory Authorities (as applicable), Ethics Committees/IRB and Principal Investigators with safety updates/reports according to local requirements, including SUSARs (Suspected Unexpected Serious Adverse Reactions), where relevant.

Each Principal Investigator is responsible for providing the Ethics Committees/IRB with reports of any serious and unexpected adverse drug reactions that occur with the study medication during the study, according to their local Ethics Committee/IRB regulations.

9.4 Informed consent

The Principal Investigator or delegate at each center will:

- Ensure each patient is given full and adequate oral and written information about the nature, purpose, possible risk and benefit of the study
- Ensure each patient is notified that they are free to discontinue from the study at any time
- Ensure that each patient is given the opportunity to ask questions and allowed time to consider the information provided
- Ensure each patient provides signed and dated informed consent before conducting any study specific procedure
- Ensure the original, signed Informed Consent Form(s) is/are stored in the study regulatory binder Ensure a copy of the signed Informed Consent Form is given to the patient

CONFIDENTIAL

- Ensure that any incentives for patients who participate in the study as well as any provisions for patients harmed as a consequence of study participation are described in the informed consent form that is approved by an Ethics Committee.

9.5 Changes to the protocol and informed consent form

Study procedures will not be changed without the mutual agreement of the EC and [redacted]

If there are any substantial changes to the study protocol, then these changes will be documented in a study protocol amendment and where required in a new version of the study protocol (Revised Clinical Study Protocol).

[redacted] will distribute any subsequent amendments and new versions of the protocol to each Principal Investigator(s). If a protocol amendment requires a change to a site's Informed Consent Form, [redacted] and the site's IRB are to approve the revised Informed Consent Form before the revised form is used.

Any administrative change(s) and protocol amendment(s) will be prepared and approved by the study sponsor ([redacted] subsequently communicated to the EC.

9.6 Audits and inspections

Authorized representatives of [redacted], a regulatory authority, or an Ethics Committee may perform audits or inspections at the center, including source data verification. The purpose of an audit or inspection is to systematically and independently examine all study- related activities and documents, to determine whether these activities were conducted, and data were recorded, analyzed, and accurately reported according to the protocol, GCP, guidelines of the International Conference on Harmonization (ICH), and any applicable regulatory requirements. The investigator will contact [redacted] immediately if contacted by a regulatory agency about an inspection at the center.

9.7 Posting of information on clinicaltrials.gov

Study information from this protocol will be posted on clinicaltrials.gov before enrollment of patients begins.

10 STUDY MANAGEMENT BY [redacted]

10.1 Pre-study activities

Before the first patient is entered into the study, it is necessary for a representative of [redacted] to evaluate the investigational study site to:

- Determine the adequacy of the facilities
- Determine availability of appropriate patients for the study
- Discuss with the investigator(s) (and other personnel involved with the study) their responsibilities with regard to protocol adherence, and the responsibilities of [redacted] or its representatives. This will be documented in a Clinical Study Agreement between [redacted] and the investigator.

10.2 Training of study site personnel

Before the first patient is entered into the study, a [redacted] representative will review and discuss the requirements of the Clinical Study Protocol and related documents with the investigational staff and also train them in any study specific procedures and the WBDC system(s) utilized.

The Principal Investigator will ensure that appropriate training relevant to the study is given to all of these staff, and that any new information relevant to the performance of this study is forwarded to the staff involved.

The Principal Investigator will maintain a record of all individuals involved in the study (medical, nursing and other staff).

10.3 Monitoring of the study

During the study, a [redacted] representative will conduct regular monitoring visits with the study site. The monitoring visits may be conducted by phone, e-mail or by in-person visits to the study site. The monitoring visits will:

- Provide information and support to the investigator(s)
- Confirm that facilities remain acceptable
- Confirm that the investigational team is adhering to the protocol, that data are being accurately and timely recorded in the eCRF and that study drug accountability checks are being performed
- Perform source data verification (a comparison of the data in the eCRF with the patient's medical records at the hospital or practice, and other records relevant to the study) including verification of informed consent of participating patients.

The [redacted] representative will be available between visits if the investigator(s) or other study site personnel need information and advice about the study conduct.

10.4 Source data

The Clinical Study Agreement (CSA) will specify the location of source data. Access to source documents and source data is essential to inspection and review of clinical studies by the Food and Drug Administration (FDA).

10.5 Study agreements

The Principal Investigator at each center should comply with all the terms, conditions, and obligations of the CSA, or equivalent, for this study. In the event of any inconsistency between this Clinical Study Protocol and the CSA, the terms of Clinical Study Protocol shall prevail with respect to the conduct of the study and the treatment of patients and in all other respects, not relating to study conduct or treatment of patients, the terms of the CSA shall prevail.

Agreements between [redacted] and the Principal Investigator should be in place before any study-related procedures can take place, or patients are enrolled.

10.6 Archiving of study documents

The Investigator follows the principles outlined in the CSA.

10.7 Study timetable and end of study

The study is expected to start in December 2016 and to end in October 2018. Planned treatment duration in the study is 13 weeks. [redacted] will notify investigators when

CONFIDENTIAL

recruitment is complete. The end of the entire study is defined as 'the last visit of the last patient undergoing the study'.

The study may be terminated at individual centers if the study procedures are not being performed according to GCP, or if recruitment is slow. [redacted] may also terminate the entire study prematurely if concerns for safety arise within this study or in any other study with dapagliflozin.

11 DATA MANAGEMENT BY [redacted]

Data management will be performed by [redacted] Data Management Center staff. Data will be entered in the WBDC system at the study site. Trained site staff will be responsible for entering data on the observations, tests and assessments specified in the protocol into the WBDC system. Data entered in the WBDC system will be immediately saved to a central database and changes tracked to provide an audit trail. The data will then be source data verified, reviewed/queried and updated as needed. The Principal Investigator is responsible for electronically signing the eCRF. Data queries will be raised for inconsistent, improbable or missing data. All entries to the study database will be available in an audit trail. The data will be frozen and then locked to prevent further editing. When all data have been coded, validated, signed, and locked, a clean file will be declared. Any treatment revealing data may thereafter be added and the final database will be locked. A copy of the eCRF will be archived at the study site when the study has been locked.

12 STATISTICAL METHODS AND SAMPLE SIZE DETERMINATION

12.1 Description of analysis sets

12.1.1 Efficacy analysis set

Intention to treat (ITT) is defined as all patients who have been randomized to study treatment and completed at least one follow-up where NTproBNP is collected. The ITT data set will be used for the primary and secondary efficacy endpoints and exploratory endpoints.

12.1.2 Safety analysis set

All patients who received at least 1 dose of randomized dapagliflozin or placebo, and for whom post-dose data are available, will be included in the safety analysis set. Throughout the safety results sections, erroneously treated patients (eg, those randomized to dapagliflozin but actually given placebo) will be accounted for in the actual treatment group. If a patient received study drug from the wrong kit for only a part of the treatment duration and then switched to another, the associated actual treatment group for that patient will be the treatment group the patient had the longest exposure to.

12.2 Methods of statistical analyses

Randomization will be stratified by diabetes status (diabetes vs. no-diabetes), permanent atrial fibrillation status (Yes vs. No), and by patients' participation in the echocardiography sub-study.

Baseline demographic and clinical data will be described between treatment and placebo study arms as mean ± standard deviation for continuous variables and compared using Student's T-test. Whereas discrete variables will be represented as a number and (%) and compared using the χ^2 or Fisher's exact test, as appropriate.

The time course of continuous variables will be presented using standard descriptive summary statistics calculated at each scheduled measuring time point and the last individual measuring time point. Moreover, standard descriptive summary statistics will be calculated for the change (absolute or percent) from baseline to each scheduled measuring time point after baseline and the last individual measuring time point.

Statistical significance will be defined using two-sided tests with α=0.05, unless otherwise specified. All statistical analyses will be performed by the [redacted] of Biostatistics using SAS version 9.4 (SAS Institute, Cary, North Carolina).

The primary endpoint of the study is to compare health status change with dapagliflozin versus placebo as measured by KCCQ clinical summary scores (KCCQ CS) at 12 weeks. This will be analyzed using ANCOVA adjusting for baseline KCCQ CS, gender, eGFR, Diabetes status, permanent atrial fibrillation status, and LVEF. Patient participation in the Echocardiography sub-study will not be controlled for because we don't hypothesize that participation in the Echocardiography sub-study is associated with the outcome (KCCQ CS).

For the primary endpoint a sample size of 145 for each group will achieve 82% power with α=0.05 to detect a 4.7 difference in mean KCCQ CS between dapagliflozin group and placebo group at 12 weeks. The assumptions for this calculation was derived from Define-HF study where the adjusted mean difference between dapagliflozin group and placebo group is 4.7 and the standard deviation is 13.7. Assuming a 10% loss to follow up, we arrive at a sample size of ~320 patients.

We plan to repeat the analysis within the subgroups of patients with and without diabetes. Assuming 60% of the patients do not have type 2 diabetes, the non-diabetes stratum is estimated to have ~174 patients (~87 each arm), which will achieve 80% power with α=0.05 to detect a 5.9 difference in mean KCCQ CS between dapagliflozin and placebo at 12 weeks; assuming 10% loss to follow up, we arrive at a sample size of ~192 patients within the no diabetes subgroup (~60% of the total sample). This will provide a large enough subgroup of patients with no diabetes to provide preliminary data on the effects of dapagliflozin vs. placebo in this important subgroup – in which the effects of SGLT2 inhibitors have not been well described. For the diabetes subgroup, a sample size of 58 for each arm will achieve 80% power with α=0.05 to detect a 7.2 difference in mean KCCQ CS between dapagliflozin and placebo at 12 weeks.; assuming 10% loss to follow up, we arrive at a sample size of ~128 patients within the diabetes subgroup (40% of the total sample). The above calculations demonstrate that the current overall study sample size is sufficient to examine the effects of dapagliflozin vs. placebo on the primary endpoint; and will provide sufficient numbers of patients within the key subgroups (diabetes, no diabetes) to examine whether important interactions exist in the effects of dapagliflozin vs. placebo by diabetes status.

Of note, participation of patients with permanent atrial fibrillation is allowed, but may be capped at no more than 33% (105 patients) of the entire cohort.

Due to the large number of study sites and the expected low number of patients per site it will not be appropriate to explore site effects.

12.2.1 Primary Outcome

The primary endpoint of the study is to compare health status change with dapagliflozin versus placebo as measured by KCCQ clinical summary scores (KCCQ CS) at 12 weeks. This will be analyzed using ANCOVA adjusting for baseline KCCQ CS, gender, eGFR, Diabetes status, permanent atrial fibrillation status, and LVEF. Patient participation in the Echocardiography sub-study will not be controlled for because we don't hypothesize that participation in the Echocardiography sub-study is associated with the outcome (KCCQ CS).

12.2.2 Secondary Outcomes

1. Change from baseline in heart failure related health status using the Kansas City Cardiomyopathy Questionnaire (KCCQ) overall summary score at 12 weeks
2. Change from baseline in NTproBNP at 6 and 12 weeks
3. Change from baseline in BNP at 6 and 12 weeks
4. Change from baseline in 6 minute walk test at 12 weeks
5. Change from baseline in BNP at 6 and 12 weeks
6. Change from baseline in HbA1c over the treatment period
7. Proportion of patients with a ≥ 5pts increase in KCCQ clinical summary score and KCCQ overall summary score at 12 weeks
8. Proportion of patients with a ≥ 20% decrease in NTproBNP at 6 and 12 weeks
9. Proportion of patients with a ≥ 5pts increase in KCCQ and a ≥ 20% decrease in NTproBNP at 6 and 12 weeks
10. Change in weight at 6 and 12 weeks
11. Change in systolic blood pressure at 6 and 12 weeks

12.2.3 Exploratory Outcomes

1. Composite mean hierarchical-rank clinical score between dapagliflozin vs. placebo. All patients will receive a global rank endpoint based on time to death (tier 1) time to HF hospitalization or urgent HF visit (tier 2) or change in KCCQ clinical summary score from baseline to 12 weeks
2. Number of heart failure hospitalizations
3. Number of urgent heart failure visits

CONFIDENTIAL

4. Number of heart failure hospitalizations and urgent heart failure visits
5. Proportion of patients that progress to diabetes during the treatment period (within the subgroup of patients with no diabetes at baseline only)
6. Change from baseline in average weekly loop diuretic dose (furosemide equivalent)
7. Change in NYHA Class at 6 and 12 weeks.
8.
9. Change from baseline in left atrial volume index and other measures of left ventricular diastolic function

12.2.4 Safety Outcomes

1. All cause death
2. Cardiovascular death
3. Non-fatal myocardial infarction (MI)
4. Stroke
5. Acute kidney injury (defined as doubling of serum creatinine based on modified RIFLE criteria)
6. Adverse events (AEs) and serious adverse events (SAEs). AEs of special interest will include DKA, volume depletion (defined as hypotension, syncope, orthostatic hypotension or dehydration), severe hypoglycemic events and lower limb amputations.

12.2.5 Analysis for safety

Safety analyses will be done periodically during the study and reported to the IDSMC. A formal IDSMC charter will be developed.

12.3 Determination of sample size

See section 12.2.

13 IMPORTANT MEDICAL PROCEDURES TO BE FOLLOWED BY THE INVESTIGATOR

13.1 Medical emergencies and [redacted] Contacts

The Principal Investigator is responsible for ensuring that procedures and expertise are available to handle medical emergencies during the study. A medical emergency usually constitutes an SAE and is to be reported as such.

In the case of a medical emergency the investigator may contact the Study Team Physician at [redacted]

CONFIDENTIAL

Name	Role in the study	Address & telephone number
[redacted]	Lead Study Team Physician responsible for the protocol	[redacted]
[redacted]	Study Manager	[redacted]

13.2 Overdose

Overdose is defined as the accidental or intentional ingestion of any dose of investigational product that is considered both excessive and medically important. Dapagliflozin has been well tolerated at doses of up to 500 mg/day in single dose testing in healthy volunteers and up to 100 mg/day in repeat dose testing for 14 days in healthy volunteers and patients with type 2 diabetes. If an overdose is suspected, monitoring of vital functions as well as treatment, as appropriate, should be performed. If an overdose occurrence meets the criteria for a Serious Adverse Event, then it must be reported as Serious Adverse Event.

13.3 Pregnancy

Any pregnancy during the course of this study should be recorded. Pregnancy itself is not regarded as an Adverse Event unless there is a suspicion that the investigational product under study may have interfered with the effectiveness of a contraceptive medication.

If any pregnancy occurs in the course of the study, the patient should be discontinued, the investigational product should be stopped and then investigators or other site personnel must inform appropriate [redacted] representatives immediately but no later than the end of the next business day of when he or she becomes aware of it.

All pregnancies and outcomes of pregnancy should be reported to [redacted] designated fax line: +1 302 886 4114 or email if a secure line is set up: [redacted].

14 ECHOCARDIOGRAPHY SUBSTUDY

An echocardiography substudy will be conducted. Patients will undergo echocardiogram at the Randomization and Week 12 visits to assess the impact of dapagliflozin versus placebo on left atrial volume index and other parameters of diastolic function. Detailed collection, processing, and uploading instructions will be provided in the central echocardiography core imaging laboratory manual. Information regarding the echo substudy is included in appendix C.

CONFIDENTIAL

15 FUTURE RESEARCH

A portion of the blood samples (banked biospecimens) collected at the Randomization and Week 12 visits will be stored indefinitely to allow for possible future research to study genes (DNA), RNA, proteins and biomarkers. Separate research proposals will be developed and IRB approvals obtained prior to this research.

The banked biospecimens will be kept in a secure location at [redacted]. To protect patient confidentiality, the banked biospecimens and data generated from them will be coded only with a study ID number. Data will be stored on password-protected computer systems. The key between the code and personal identifiers will be held at the study site collecting the samples. The researchers using the biospecimens (and data generated from them) will not have access to the key nor any personally identifying information. Data generated from the banked biospecimens will be kept by [redacted].

It is very unlikely that results generated from the banked biospecimens will have any clinical, diagnostic, or therapeutic implications for individual study participants. Results from the banked biospecimens will not be returned to the study site or to the study participant, their doctor, family members or recorded in their medical record.

16 REFERENCES

1. Solomon SD, Zile M, Pieske B, Voors A, Shah A, Kraigher-Krainer E, Shi V, Bransford T, Takeuchi M, Gong J, Lefkowitz M, Packer M, McMurray JJ and Prospective comparison of AwARBoMOhfwpefI. The angiotensin receptor neprilysin inhibitor LCZ696 in heart failure with preserved ejection fraction: a phase 2 double-blind randomised controlled trial. *Lancet*. 2012;380:1387-95.
2. McMurray JJ, Packer M, Desai AS, Gong J, Lefkowitz MP, Rizkala AR, Rouleau JL, Shi VC, Solomon SD, Swedberg K, Zile MR, Investigators P-H and Committees. Angiotensin-neprilysin inhibition versus enalapril in heart failure. *N Engl J Med*. 2014;371:993-1004.
3. Zinman B, Wanner C, Lachin JM, Fitchett D, Bluhmki E, Hantel S, Mattheus M, Devins T, Johansen OE, Woerle HJ, Broedl UC, Inzucchi SE and Investigators E-RO. Empagliflozin, Cardiovascular Outcomes, and Mortality in Type 2 Diabetes. *N Engl J Med*. 2015.
4. Owan TE and Redfield MM. Epidemiology of diastolic heart failure. *Prog Cardiovasc Dis*. 2005;47:320-32.
5. Owan TE, Hodge DO, Herges RM, Jacobsen SJ, Roger VL and Redfield MM. Trends in prevalence and outcome of heart failure with preserved ejection fraction. *N Engl J Med*. 2006;355:251-9.
6. Lindman BR, Dávila-Román VG, Mann DL, McNulty S, Semigran MJ, Lewis GD, de las Fuentes L, Joseph SM, Vader J, Hernandez AF and Redfield MM. Cardiovascular Phenotype in HFpEF Patients With or Without Diabetes: A RELAX Trial Ancillary Study. *Journal of the American College of Cardiology*. 2014;64:541-549.
7. Kannel WB and McGee DL. Diabetes and cardiovascular disease. The Framingham study. *JAMA*. 1979;241:2035-8.
8. MacDonald MR, Petrie MC, Varyani F, Ostergren J, Michelson EL, Young JB, Solomon SD, Granger CB, Swedberg K, Yusuf S, Pfeffer MA, McMurray JJ and Investigators C. Impact of diabetes on outcomes in patients with low and preserved ejection fraction heart failure: an analysis of the Candesartan in

CONFIDENTIAL

Heart failure: Assessment of Reduction in Mortality and morbidity (CHARM) programme. *Eur Heart J*. 2008;29:1377-85.
9. Kao DP, Lewsey JD, Anand IS, Massie BM, Zile MR, Carson PE, McKelvie RS, Komajda M, McMurray JJ and Lindenfeld J. Characterization of subgroups of heart failure patients with preserved ejection fraction with possible implications for prognosis and treatment response. *Eur J Heart Fail*. 2015;17:925-35.
10. Redfield MM, Chen HH, Borlaug BA and et al. Effect of phosphodiesterase-5 inhibition on exercise capacity and clinical status in heart failure with preserved ejection fraction: A randomized clinical trial. *JAMA*. 2013;309:1268-1277.
11. Shah SJ, Heitner JF, Sweitzer NK, Anand IS, Kim HY, Harty B, Boineau R, Clausell N, Desai AS, Diaz R, Fleg JL, Gordeev I, Lewis EF, Markov V, O'Meara E, Kobulia B, Shaburishvili T, Solomon SD, Pitt B, Pfeffer MA and Li R. Baseline characteristics of patients in the treatment of preserved cardiac function heart failure with an aldosterone antagonist trial. *Circ Heart Fail*. 2013;6:184-92.
12. Ingwall JS and Weiss RG. Is the failing heart energy starved? On using chemical energy to support cardiac function. *Circ Res*. 2004;95:135-45.
13. Pitt B, Pfeffer MA, Assmann SF, Boineau R, Anand IS, Claggett B, Clausell N, Desai AS, Diaz R, Fleg JL, Gordeev I, Harty B, Heitner JF, Kenwood CT, Lewis EF, O'Meara E, Probstfield JL, Shaburishvili T, Shah SJ, Solomon SD, Sweitzer NK, Yang S, McKinlay SM and Investigators T. Spironolactone for heart failure with preserved ejection fraction. *N Engl J Med*. 2014;370:1383-92.
14. Inzucchi SE, Zinman B, Wanner C, Ferrari R, Fitchett D, Hantel S, Espadero RM, Woerle HJ, Broedl UC and Johansen OE. SGLT-2 inhibitors and cardiovascular risk: proposed pathways and review of ongoing outcome trials. *Diab Vasc Dis Res*. 2015;12:90-100.
15. Oelze M, Kroller-Schon S, Welschof P, Jansen T, Hausding M, Mikhed Y, Stamm P, Mader M, Zinssius E, Agdauletova S, Gottschlich A, Steven S, Schulz E, Bottari SP, Mayoux E, Munzel T and Daiber A. The sodium-glucose co-transporter 2 inhibitor empagliflozin improves diabetes-induced vascular dysfunction in the streptozotocin diabetes rat model by interfering with oxidative stress and glucotoxicity. *PLoS One*. 2014;9:e112394.
16. Guyatt GH, Sullivan MJ, Thompson PJ, Fallen EL, Pugsley SO, Taylor DW and Berman LB. The 6-minute walk: a new measure of exercise capacity in patients with chronic heart failure. *Can Med Assoc J*. 1985;132:919-23.
17. Hauptman PJ, Masoudi FA, Weintraub WS, Pina I, Jones PG and Spertus JA. Variability in the clinical status of patients with advanced heart failure. *J Card Fail*. 2004;10:397-402.
18. Thygesen K, Alpert JS, Jaffe AS, Simoons ML, Chaitman BR, White HD, Katus HA, Lindahl B, Morrow DA, Clemmensen PM, Johanson P, Hod H, Underwood R, Bax JJ, Bonow RO, Pinto F, Gibbons RJ, Fox KA, Atar D, Newby LK, Galvani M, Hamm CW, Uretsky BF, Steg PG, Wijns W, Bassand JP, Menasche P, Ravkilde J, Ohman EM, Antman EM, Wallentin LC, Armstrong PW, Januzzi JL, Nieminen MS, Gheorghiade M, Filippatos G, Luepker RV, Fortmann SP, Rosamond WD, Levy D, Wood D, Smith SC, Hu D, Lopez-Sendon JL, Robertson RM, Weaver D, Tendera M, Bove AA, Parkhomenko AN, Vasilieva EJ and Mendis S. Third universal definition of myocardial infarction. *Circulation*. 2012;126:2020-35.
19. Mak KH and Topol EJ. Emerging concepts in the management of acute myocardial infarction in patients with diabetes mellitus. *J Am Coll Cardiol*. 2000;35:563-8.
20. Devereux RB, Roman MJ, Paranicas M, O'Grady MJ, Lee ET, Welty TK, Fabsitz RR, Robbins D, Rhoades ER and Howard BV. Impact of diabetes on cardiac structure and function: the strong heart study. *Circulation*. 2000;101:2271-6.
21. Abbott RD, Donahue RP, Kannel WB and Wilson PW. The impact of diabetes on survival following myocardial infarction in men vs women. The Framingham Study. *JAMA*. 1988;260:3456-60.

CONFIDENTIAL

22. Bella JN, Devereux RB, Roman MJ, Palmieri V, Liu JE, Paranicas M, Welty TK, Lee ET, Fabsitz RR and Howard BV. Separate and joint effects of systemic hypertension and diabetes mellitus on left ventricular structure and function in American Indians (the Strong Heart Study). *Am J Cardiol*. 2001;87:1260-5.
23. Palmieri V, Bella JN, Arnett DK, Liu JE, Oberman A, Schuck MY, Kitzman DW, Hopkins PN, Morgan D, Rao DC and Devereux RB. Effect of type 2 diabetes mellitus on left ventricular geometry and systolic function in hypertensive subjects: Hypertension Genetic Epidemiology Network (HyperGEN) study. *Circulation*. 2001;103:102-7.
24. Lainchbury JG, Troughton RW, Strangman KM, Frampton CM, Pilbrow A, Yandle TG, Hamid AK, Nicholls MG and Richards AM. N-terminal pro-B-type natriuretic peptide-guided treatment for chronic heart failure: results from the BATTLESCARRED (NT-proBNP-Assisted Treatment To Lessen Serial Cardiac Readmissions and Death) trial. *J Am Coll Cardiol*. 2009;55:53-60.
25. Pfisterer M, Buser P, Rickli H, Gutmann M, Erne P, Rickenbacher P, Vuillomenet A, Jeker U, Dubach P, Beer H, Yoon SI, Suter T, Osterhues HH, Schieber MM, Hilti P, Schindler R, Brunner-La Rocca HP and Investigators T-C. BNP-guided vs symptom-guided heart failure therapy: the Trial of Intensified vs Standard Medical Therapy in Elderly Patients With Congestive Heart Failure (TIME-CHF) randomized trial. *JAMA*. 2009;301:383-92
26. Yancy CW, Jessup M, Bozkurt B, Butler J, Casey DE, Jr., Drazner MH, Fonarow GC, Geraci SA, Horwich T, Januzzi JL, Johnson MR, Kasper EK, Levy WC, Masoudi FA, McBride PE, McMurray JJ, Mitchell JE, Peterson PN, Riegel B, Sam F, Stevenson LW, Tang WH, Tsai EJ, Wilkoff BL, American College of Cardiology F and American Heart Association Task Force on Practice G. 2013 ACCF/AHA guideline for the management of heart failure: a report of the American College of Cardiology Foundation/American Heart Association Task Force on Practice Guidelines. *J Am Coll Cardiol*. 2013;62:e147-239
27. EMPagliflozin outcomE tRial in Patients With chrOnic heaRt Failure With Reduced Ejection Fraction (EMPEROR-Reduced). ClinicalTrials.gov 2017; https://clinicaltrials.gov/ct2/show/NCT03057977?term=03057977&rank=1
28. EMPagliflozin outcomE tRial in Patients With chrOnic heaRt Failure With Preserved Ejection Fraction (EMPEROR-Preserved). ClinicalTrials.gov 2017; https://clinicaltrials.gov/ct2/show/NCT03057951?term=03057951&rank=1
29. Study to Evaluate the Effect of Dapagliflozin on the Incidence of Worsening Heart Failure or Cardiovascular Death in Patients With Chronic Heart Failure (Dapa-HF). ClinicalTrials.gov 2017; https://clinicaltrials.gov/ct2/show/NCT03036124?term=03036124&rank=1

CONFIDENTIAL

17 APPENDIX A: HEART FAILURE HOSPITALIZATION/URGENT OUTPATIENT VISIT

A **Heart Failure Event** includes hospitalization for heart failure and may include urgent outpatient visits. Heart failure hospitalizations should remain delineated from urgent visits.

A **Heart Failure Hospitalization** is defined as an event that meets ALL of the following criteria:

- The patient is admitted to the hospital with a primary diagnosis of heart failure
- The patient's length-of-stay in hospital extends for at least 24 hours (or a change in calendar date if the hospital admission and discharge times are unavailable)
- The patient exhibits documented new or worsening symptoms due to heart failure on presentation, including at least ONE of the following:
    - Dyspnea (dyspnea with exertion, dyspnea at rest, orthopnea, paroxysmal nocturnal dyspnea)
    - Decreased exercise tolerance
    - Fatigue
    - Other symptoms of worsened end-organ perfusion or volume overload

- The patient has objective evidence of new or worsening heart failure, consisting of at least TWO physical examination findings OR one physical examination finding and at least ONE laboratory criterion), including:
    - Physical examination findings considered to be due to heart failure, including new or worsened:
        - Peripheral edema
        - Increasing abdominal distention or ascites (in the absence of primary hepatic disease)
        - Pulmonary rales/crackles/crepitations
        - Increased jugular venous pressure and/or hepatojugular reflux
        - S3 gallop
        - Clinically significant or rapid weight gain thought to be related to fluid retention
    - Laboratory evidence of new or worsening heart failure, if obtained within 24 hours of presentation, including:
        - Increased B-type natriuretic peptide (BNP)/ N-terminal pro-BNP (NT- proBNP) concentrations consistent with decompensation of heart failure. In patients with chronically elevated natriuretic peptides, a significant increase should be noted above baseline.
        - Radiological evidence of pulmonary congestion
        - Non-invasive or invasive diagnostic evidence of clinically significant elevated left- or right-sided ventricular filling pressure or low cardiac output. For example, echocardiographic criteria could include: E/e' > 15 or D-dominant pulmonary venous inflow pattern, plethoric inferior vena cava with minimal collapse on inspiration

          OR
        - Invasive diagnostic evidence with right heart catheterization showing a pulmonary capillary wedge pressure (pulmonary artery occlusion pressure) •

CONFIDENTIAL

≥18 mmHg, central venous pressure ≥ 12 mmHg, or a cardiac index < 2.2 L/min/m^2

- The patient receives initiation or intensification of treatment specifically for heart failure, including <u>at least ONE</u> of the following:
    - Augmentation in oral diuretic therapy
    - Intravenous diuretic, inotrope, or vasodilator therapy
    - Mechanical or surgical intervention, including:
        - Mechanical circulatory support (e.g., intra-aortic balloon pump, ventricular assist device)
        - Mechanical fluid removal (e.g., ultrafiltration, hemofiltration, dialysis) Using available information, Heart Failure will be categorized based on the following:
            - Left ventricular ejection fraction (LVEF)
            - Type
            - Etiology

- An **Urgent Heart Failure Visit** is defined as an event that meets all of the following:
        - The patient has an urgent, unscheduled office/practice or emergency department visit for a primary diagnosis of heart failure, but not meeting the criteria for a heart failure hospitalization.
        - All signs and symptoms for heart failure hospitalization (i.e., 3) symptoms; 4) physical examination findings/laboratory evidence of new or worsening heart failure, as indicated above) must be met
        - The patient receives initiation or intensification of treatment specifically for heart failure, as detailed in the above section.

CONFIDENTIAL

18 APPENDIX B: KANSAS CITY CARDIOMYOPATHY QUESTIONNAIRE (KCCQ)

Kansas City Cardiomyopathy Questionnaire (KCCQ)

The following questions refer to your **heart failure** and how it may affect your life. Please read and complete the following questions. There are no right or wrong answers. Please mark the answer that best applies to you.

1. **Heart failure** affects different people in different ways. Some feel shortness of breath while others feel fatigue. Please indicate how much you are limited by **heart failure** (shortness of breath or fatigue) in your ability to do the following activities over the past 2 weeks.

**Activity**	**Extremely Limited**	**Quite a bit Limited**	**Moderately Limited**	**Slightly Limited**	**Not at all Limited**	**Limited for other reasons or did not do the activity**
Dressing yourself	☐	☐	☐	☐	☐	☐
Showering/Bathing	☐	☐	☐	☐	☐	☐
Walking 1 block on level ground	☐	☐	☐	☐	☐	☐
Doing yardwork, housework or carrying groceries	☐	☐	☐	☐	☐	☐
Climbing a flight of stairs without stopping	☐	☐	☐	☐	☐	☐
Hurrying or jogging (as if to catch a bus)	☐	☐	☐	☐	☐	☐

2. Compared with 2 weeks ago, have your symptoms of **heart failure** (shortness of breath, fatigue, or ankle swelling) changed?

My symptoms of **heart failure** have become…

Much worse	Slightly worse	Not changed	Slightly better	Much better	I've had no symptoms over the last 2 weeks
☐	☐	☐	☐	☐	☐

CONFIDENTIAL

3. Over the past 2 weeks, how many times did you have **swelling** in your feet, ankles or legs when you woke up in the morning?

Every morning	3 or more times a week, but not every day	1-2 times a week	Less than once a week	Never over the past 2 weeks
☐	☐	☐	☐	☐

4. Over the past 2 weeks, how much has **swelling** in your feet, ankles or legs bothered you?

It has been ...

**Extremely** bothersome	**Quite a bit** bothersome	**Moderately** bothersome	**Slightly** bothersome	**Not at all** bothersome	I've had **no swelling**
☐	☐	☐	☐	☐	☐

5. Over the past 2 weeks, on average, how many times has **fatigue** limited your ability to do what you want?

All of the time	Several times per day	At least once a day	3 or more times per week but not every day	1-2 times per week	Less than once a week	Never over the past 2 weeks
☐	☐	☐	☐	☐	☐	☐

6. Over the past 2 weeks, how much has your **fatigue** bothered you?

It has been ...

**Extremely** bothersome	**Quite a bit** bothersome	**Moderately** bothersome	**Slightly** bothersome	**Not at all** bothersome	I've had **no fatigue**
☐	☐	☐	☐	☐	☐

7. Over the past 2 weeks, on average, how many times has **shortness of breath** limited your ability to do what you wanted?

All of the time	Several times per day	At least once a day	3 or more times per week but not every day	1-2 times per week	Less than once a week	Never over the past 2 weeks
☐	☐	☐	☐	☐	☐	☐

CONFIDENTIAL

8. Over the <u>past 2 weeks,</u> how much has your **shortness of breath** bothered you?

It has been ...

**Extremely** bothersome	**Quite a bit** bothersome	**Moderately** bothersome	**Slightly** bothersome	**Not at all** bothersome	I've had **no shortness of breath**
☐	☐	☐	☐	☐	☐

9. Over the <u>past 2 weeks,</u> on average, how many times have you been forced to sleep sitting up in a chair or with at least 3 pillows to prop you up because of **shortness of breath**?

Every night	3 or more times a week, but not every day	1-2 times a week	Less than once a week	Never over the past 2 weeks
☐	☐	☐	☐	☐

10. **Heart failure** symptoms can worsen for a number of reasons. How sure are you that you know what to do, or whom to call, if your **heart failure** gets worse?

**Not at all** sure	**Not very** sure	**Somewhat** sure	**Mostly** sure	**Completely** sure
☐	☐	☐	☐	☐

11. How well do you understand what things you are able to do to keep your **heart failure** symptoms from getting worse? (for example, weighing yourself, eating a low salt diet etc.)

Do not understand at all	Do not understand very well	Somewhat understand	Mostly understand	Completely understand

12. Over the <u>past 2 weeks</u>, how much has your **heart failure** limited your enjoyment of life?

It has **extremely** limited my enjoyment of life	It has limited my enjoyment of life **quite a bit**	It has **moderately** limited my enjoyment of life	It has **slightly** limited my enjoyment of life	It has **not limited** my enjoyment of life at all
☐	☐	☐	☐	☐

CONFIDENTIAL

13. If you had to spend the rest of your life with your **heart failure** the way it is <u>right now</u>, how would you feel about this?

Not at all satisfied	Mostly dissatisfied	Somewhat satisfied	Mostly satisfied	Completely satisfied
☐	☐	☐	☐	☐

14. Over the <u>past 2 weeks</u>, how often have you felt discouraged or down in the dumps because of your **heart failure**?

I felt that way **all of the time**	I felt that way **most of the time**	I **occasionally** felt that way	I **rarely** felt that way	I **never** felt that way
☐	☐	☐	☐	☐

15. How much does your **heart failure** affect your lifestyle? Please indicate how your **heart failure** may have limited your participation in the following activities <u>over the past 2 weeks.</u>

Please place an **X** in one box on each line

**Activity**	**Severely** limited	Limited **quite a bit**	**Moderately** limited	**Slightly** limited	**Did not** limit at all	Does not apply or did not do for other reasons
Hobbies, recreational activities	☐	☐	☐	☐	☐	☐
Working or doing household chores	☐	☐	☐	☐	☐	☐
Visiting family or friends out of your home			☐	☐	☐	☐
Intimate relationships with loved ones	☐	☐	☐	☐	☐	☐

Place an **X** in one box on each line

CONFIDENTIAL

19 APPENDIX C: ECHOCARDIOGRAPHY SUBSTUDY

Echocardiography Sub-study

Heart failure with preserved ejection fraction (HFpEF), which currently represents approximately half of all HF cases, is a common clinical syndrome and a leading cause of morbidity and mortality, especially among the elderly.[4] Furthermore, the prevalence of heart failure with preserved systolic function is increasing.[5] In spite of this rapidly increasing economic and healthcare burden, no treatment has yet been shown to reduce morbidity and mortality in heart failure patients with HFpEF.

Diabetes adversely affects outcomes of all types of cardiovascular diseases.[6] In particular, diabetes is associated with a 70% to 80% increase in mortality and hospitalizations in patients with heart failure with HFpEF.[7-9] The recently published EMPA-REG OUTCOME trial noted a 38% relative risk reduction of cardiovascular death with SGLT-2 inhibitors, versus placebo in patients with diabetes and established CVD.[3] Though the trial was predominantly of diabetic patients with coronary artery disease, the majority of the benefit appeared to be due to the reduction in hospitalizations for heart failure (a 35% relative risk reduction). The significant reductions in HF hospitalizations and death from HF are particularly impressive given only a small minority (10%) of the patients in the trial had a heart failure diagnosis at baseline. Although the etiology of hospitalizations for heart failure was not captured (HFpEF vs. HFrEF) it stands to reason that given a population of overweight, diabetic patients with a baseline 40% diuretic use, that undiagnosed HFpEF was highly prevalent.

While the mechanism of action for such a dramatic benefit in prevention of HF and cardiovascular mortality is not well understood, there are multiple theoretical benefits of dapagliflozin on diastolic heart function. There have been numerous animal and preclinical model that showing that diabetic patients have increased markers of oxidative stress and poor endothelial function making them high risk for HFpEF.[14] We hypothesize that the majority of the benefit of SGLT-2 inhibitors in HFpEF is due to a reduction in oxidative stress, (improving diastolic function), improvement in endothelial function (thus decreasing vessel stiffness), anti-inflammatory effects and possibly inhibitory effects on sympathetic nervous system. [15]Supporting this hypothesis are rat models where SGLT-2 inhibition was shown to normalize endothelial function, reduce oxidative stress in aortic vessels, reverse a pro-inflammatory phenotype, and improve AGE/RAGE signaling all pathways of potential importance to a reduction in arterial stiffness.[15]

Adverse cardiac remodeling is highly prevalent amongst the HFpEF population, and often used as inclusion criteria in clinical trials. An echo sub study of I-PRESERVE noted LV hypertrophy or concentric remodeling was present in 59%, LA enlargement was present in 66%, and diastolic dysfunction was present in 69% of the patients.[22] Change in left atrial size has more recently been used as a primary endpoint in HFpEF trials, as it has been shown to decrease over short period of time with decongestive treatments. As far as surrogate endpoints NT-proBNP and LA size are complementary in the underlying mechanisms and time courses of their changes. Increases in NT-proBNP result from wall stress, and often indicate short-term changes in congestion. In contrast, LA size reflects longer-term changes of diastolic function reflecting chronically elevated LV filling pressure. Beyond a marker of diastolic function, decreases in LA size represent actual therapeutic structural reverse remodeling of an enlarged

LA. In multivariable analyses controlling for 7 clinical variables (including log N-terminal pro-B–type natriuretic peptide), left atrial size was independently associated with an increased risk of morbidity and mortality.[22] A moderate sized clinical trial of LCZ-696 was able to demonstrate a modest decrease in left atrial volume and volume index over a 12-week period, and a statistically significant decrease over a 36-week period, in patients with HFpEF.[1] There are multiple reasons to think dapagliflozin will be at least as if not more efficacious - and have a more rapid effect on LA volume and LA volume index than LCZ-696. LCZ-696 is titrated up over a 4-week period (due to concerns over tolerance and angioedema), whereas full dose dapagliflozin 10 mg will be started immediately in PRESERVED-HF. Additionally, in the EMPA-REG outcomes trial Kaplan Meier curves for HF hospitalization began to separate within days, whereas in PARADIGM-HF there was no separation in HF rehospitalization curves until 6 months.[2,3] Finally, LCZ-696 was compared with enalapril (known to improve outcomes in HF patients), whereas we will compare dapagliflozin with placebo in PRESERVED-HF.

Echo Sub Study brief protocol

Echocardiography will be done at randomization and at week 12. Analyses will be done at a core laboratory ([redacted]). Measurements will be made in triplicate in accordance with the recommendations of the American Society of Echocardiography. A detailed central imaging core echocardiography laboratory manual will be developed.

Patients with permanent atrial fibrillation will be excluded from the echo substudy. Randomization will be stratified by participation in the echo substudy of the PRESERVED-HF Trial to ensure equal distribution between dapagliflozin and placebo among the echo substudy participants

Research Hypothesis

Treatment with dapagliflozin 10 mg daily for 12 weeks will produce greater reductions in left atrial volume index compared with placebo in patients with type 2 diabetes (or no diabetes) and an established diagnosis of heart failure with preserved ejection fraction.

Primary Endpoint

Primary endpoint of the echo sub study will be change in left atrial volume index.

Exploratory Endpoints

Additional Echo measurement will be taken and change compared between dapagliflozin and placebo as exploratory end points:

CONFIDENTIAL

Change From Baseline in Echocardiography Parameters: Left Ventricular End (LVE) Diastolic Diameter, LVE Systolic Diameter, Septal End Diastolic Thickness, Posterior LV Wall End Diastolic Thickness, Relative Wall Thickness, Left Atrial Dimension, LVE Diastolic Volume, LVE Systolic Volume, Left Ventricular Stroke Volume, Left Atrial Volume, Left Ventricular Ejection Fraction Left Ventricular Mass, Left Ventricular Mass Index, E wave Velocity, A Wave Velocity, e' at Septal Mitral Annulus, e' at Lateral Mitral Annulus, Ratio of E to A Velocity, E/e' Ratio, Isovolumic Relaxation Time, Tricuspid Regurgitation Velocity, Left ventricular myocardial strain measurements.

Sample size calculation for primary endpoint

Assumption: left atrial index mean 35.9 ml/m^2, standard deviation 12.5 ml/m^2 based on Paramount Trial.[1] Based on the Table below, we estimate that a sample size of 124 patients will provide 80% power for the echo substudy to detect a 17.5% reduction in left atrial volume index with dapagliflozin vs. placebo. Assuming a 5% loss to follow up, we propose 130 patients to be randomized in the echo substudy of the PRESERVED-HF Trial

Reduction		80% Power	90% power
10%	3.59 ml/m^2	191	255
12.50%	4.48 ml/m^2	122	163
15%	5.38 ml/m^2	85	113
17.5%	6.28 ml/m^2	62	83
20%	7.18 ml/m^2	48	64
22.50%	8.07 ml/m^2	38	51
25%	8.97 ml/m^2	31	41
27.50%	9.87 ml/m^2	22	29

References

1. Solomon SD, Zile M, Pieske B, Voors A, Shah A, Kraigher-Krainer E, Shi V, Bransford T, Takeuchi M, Gong J, Lefkowitz M, Packer M, McMurray JJ and Prospective comparison of AwARBoMOhfwpefI. The angiotensin receptor neprilysin inhibitor LCZ696 in heart failure with preserved ejection fraction: a phase 2 double-blind randomised controlled trial. *Lancet*. 2012;380:1387-95.
2. McMurray JJ, Packer M, Desai AS, Gong J, Lefkowitz MP, Rizkala AR, Rouleau JL, Shi VC, Solomon SD, Swedberg K, Zile MR, Investigators P-H and Committees. Angiotensin-neprilysin inhibition versus enalapril in heart failure. *N Engl J Med*. 2014;371:993-1004.

3. Zinman B, Wanner C, Lachin JM, Fitchett D, Bluhmki E, Hantel S, Mattheus M, Devins T, Johansen OE, Woerle HJ, Broedl UC, Inzucchi SE and Investigators E-RO. Empagliflozin, Cardiovascular Outcomes, and Mortality in Type 2 Diabetes. *N Engl J Med*. 2015.
4. Owan TE and Redfield MM. Epidemiology of diastolic heart failure. *Prog Cardiovasc Dis*. 2005;47:320-32.
5. Owan TE, Hodge DO, Herges RM, Jacobsen SJ, Roger VL and Redfield MM. Trends in prevalence and outcome of heart failure with preserved ejection fraction. *N Engl J Med*. 2006;355:251-9.
6. Kannel WB and McGee DL. Diabetes and cardiovascular disease. The Framingham study. *JAMA*. 1979;241:2035-8.
7. MacDonald MR, Petrie MC, Varyani F, Ostergren J, Michelson EL, Young JB, Solomon SD, Granger CB, Swedberg K, Yusuf S, Pfeffer MA, McMurray JJ and Investigators C. Impact of diabetes on outcomes in patients with low and preserved ejection fraction heart failure: an analysis of the Candesartan in Heart failure: Assessment of Reduction in Mortality and morbidity (CHARM) programme. *Eur Heart J*. 2008;29:1377-85.
8. Kao DP, Lewsey JD, Anand IS, Massie BM, Zile MR, Carson PE, McKelvie RS, Komajda M, McMurray JJ and Lindenfeld J. Characterization of subgroups of heart failure patients with preserved ejection fraction with possible implications for prognosis and treatment response. *Eur J Heart Fail*. 2015;17:925-35.
9. Lindman BR, Dávila-Román VG, Mann DL, McNulty S, Semigran MJ, Lewis GD, de las Fuentes L, Joseph SM, Vader J, Hernandez AF and Redfield MM. Cardiovascular Phenotype in HFpEF Patients With or Without Diabetes: A RELAX Trial Ancillary Study. *Journal of the American College of Cardiology*. 2014;64:541-549.
10. Redfield MM, Chen HH, Borlaug BA and et al. Effect of phosphodiesterase-5 inhibition on exercise capacity and clinical status in heart failure with preserved ejection fraction: A randomized clinical trial. *JAMA*. 2013;309:1268-1277.
11. Shah SJ, Heitner JF, Sweitzer NK, Anand IS, Kim HY, Harty B, Boineau R, Clausell N, Desai AS, Diaz R, Fleg JL, Gordeev I, Lewis EF, Markov V, O'Meara E, Kobulia B, Shaburishvili T, Solomon SD, Pitt B, Pfeffer MA and Li R. Baseline characteristics of patients in the treatment of preserved cardiac function heart failure with an aldosterone antagonist trial. *Circ Heart Fail*. 2013;6:184-92.
12. Ingwall JS and Weiss RG. Is the failing heart energy starved? On using chemical energy to support cardiac function. *Circ Res*. 2004;95:135-45.
13. Pitt B, Pfeffer MA, Assmann SF, Boineau R, Anand IS, Claggett B, Clausell N, Desai AS, Diaz R, Fleg JL, Gordeev I, Harty B, Heitner JF, Kenwood CT, Lewis EF, O'Meara E, Probstfield JL, Shaburishvili T, Shah SJ, Solomon SD, Sweitzer NK, Yang S, McKinlay SM and Investigators T. Spironolactone for heart failure with preserved ejection fraction. *N Engl J Med*. 2014;370:1383-92.
14. Inzucchi SE, Zinman B, Wanner C, Ferrari R, Fitchett D, Hantel S, Espadero RM, Woerle HJ, Broedl UC and Johansen OE. SGLT-2 inhibitors and cardiovascular risk: proposed pathways and review of ongoing outcome trials. *Diab Vasc Dis Res*. 2015;12:90-100.
15. Oelze M, Kroller-Schon S, Welschof P, Jansen T, Hausding M, Mikhed Y, Stamm P, Mader M, Zinssius E, Agdauletova S, Gottschlich A, Steven S, Schulz E, Bottari SP, Mayoux E, Munzel T and Daiber A. The sodium-glucose co-transporter 2 inhibitor empagliflozin improves diabetes-induced vascular dysfunction in the streptozotocin diabetes rat model by interfering with oxidative stress and glucotoxicity. *PLoS One*. 2014;9:e112394.
16. Kosiborod M, Gause-Nilsson I, Sonesson C and Johnsson E. Efficacy and safety of dapagliflozin in patients with type 2 diabetes mellitus and concomitant heart failure. *DIABETOLOGIA*. 2015;58:S368-S368.

CONFIDENTIAL

17. Guyatt GH, Sullivan MJ, Thompson PJ, Fallen EL, Pugsley SO, Taylor DW and Berman LB. The 6-minute walk: a new measure of exercise capacity in patients with chronic heart failure. *Can Med Assoc J*. 1985;132:919-23.
18. Hauptman PJ, Masoudi FA, Weintraub WS, Pina I, Jones PG and Spertus JA. Variability in the clinical status of patients with advanced heart failure. *J Card Fail*. 2004;10:397-402.
19. Thygesen K, Alpert JS, Jaffe AS, Simoons ML, Chaitman BR, White HD, Katus HA, Lindahl B, Morrow DA, Clemmensen PM, Johanson P, Hod H, Underwood R, Bax JJ, Bonow RO, Pinto F, Gibbons RJ, Fox KA, Atar D, Newby LK, Galvani M, Hamm CW, Uretsky BF, Steg PG, Wijns W, Bassand JP, Menasche P, Ravkilde J, Ohman EM, Antman EM, Wallentin LC, Armstrong PW, Januzzi JL, Nieminen MS, Gheorghiade M, Filippatos G, Luepker RV, Fortmann SP, Rosamond WD, Levy D, Wood D, Smith SC, Hu D, Lopez-Sendon JL, Robertson RM, Weaver D, Tendera M, Bove AA, Parkhomenko AN, Vasilieva EJ and Mendis S. Third universal definition of myocardial infarction. *Circulation*. 2012;126:2020-35.
20. Redfield MM, Anstrom KJ, Levine JA, Koepp GA, Borlaug BA, Chen HH, LeWinter MM, Joseph SM, Shah SJ, Semigran MJ, Felker GM, Cole RT, Reeves GR, Tedford RJ, Tang WH, McNulty SE, Velazquez EJ, Shah MR, Braunwald E and Network NHFCR. Isosorbide Mononitrate in Heart Failure with Preserved Ejection Fraction. *N Engl J Med*. 2015.
21. Edelmann F, Wachter R, Schmidt AG, Kraigher-Krainer E, Colantonio C, Kamke W, Duvinage A, Stahrenberg R, Durstewitz K, Loffler M, Dungen HD, Tschope C, Herrmann-Lingen C, Halle M, Hasenfuss G, Gelbrich G, Pieske B and Aldo DHFI. Effect of spironolactone on diastolic function and exercise capacity in patients with heart failure with preserved ejection fraction: the Aldo-DHF randomized controlled trial. *JAMA*. 2013;309:781-91.
22. Zile MR, Gottdiener JS, Hetzel SJ, McMurray JJ, Komajda M, McKelvie R, Baicu CF, Massie BM, Carson PE and Investigators IP. Prevalence and significance of alterations in cardiac structure and function in patients with heart failure and a preserved ejection fraction. *Circulation*. 2011;124:2491-501.

CONFIDENTIAL

20 APPENDIX D: COVID-19

On March 11, 2020, the World Health Organization declared the 2019 novel coronavirus – and COVID-19, disease caused by the virus – a global pandemic. In response, efforts to contain the spread of COVID-19 have been implemented across the United States and have impacted the conduct of clinical research trials. In an effort to maintain, as much as possible, the integrity of the PRESERVED-HF trial so that the data can be used to answer the questions that the trial intended, certain adjustments in visit procedures may be considered on a case-by-case basis, as outlined below. The Sponsor should be notified before making these adjustments.

- **Screening Visit (Visit S) – office visit:**
  - Screening activities may be suspended at sites until such time that it is considered appropriate by the Sponsor and the site to resume activities
- **Randomization Visit (Visit 1) – office visit:**
  - For those patients that have already been screened and qualify but have yet not been randomized, the time between Screening and Randomization may be extended past 2 weeks without the need for rescreening
- **Visit 2 (Day 2) , 3 (Day 10) , 4 (Week 4) and 6 (Week 9) - phone visits:**
  - Conduct visits, as scheduled, by phone
- **Visit 5 (Week 6) - office visit:**
  - An office visit is preferred
  - The visit could be delayed and the visit window extended to accommodate an office visit
  - Conducting the visit by phone could be considered
  - If conducted by phone, complete the phone visit procedures outlined in section 6.2.3
- **Visit 7 (Week 12) – office visit:**
  - An office visit is needed to collect study endpoints (BNP, NTproBNP, KCCQ, 6-minute walk test)
  - The visit could be delayed and the visit window extended to accommodate an office visit
  - If needed, study drug could potentially be mailed by sites to a patient to keep them on study drug (depending upon local regulations) until such time that the Week 12 visit can be conducted in the office
  - If conducted by phone, complete the phone visit procedures outlined in section 6.2.3
- **Visit 8 (Week 13) – office visit:**
  - An office visit is preferred
  - Conducting the visit by phone could be considered

In the event that the COVID-19 emergency requires a protracted halt in patient enrollment, early termination of the trial may be possible. The decision will be made, if necessary, in consultation with the Executive Committee.

CONFIDENTIAL

21 APPENDIX E: PRINCIPAL INVESTIGATOR SIGNATURE

SIGNATURE OF PRINCIPAL INVESTIGATOR

EFFECTS OF DAPAGLIFLOZIN ON BIOMARKERS, SYMPTOMS AND FUNCTIONAL STATUS IN PATIENTS WITH PRESERVED EJECTION FRACTION HEART FAILURE (PRESERVED-HF TRIAL)

I agree to the terms of this study protocol. I will conduct the study according to the procedures specified herein, and according to the principles of Good Clinical Practice and local regulations.

Site Number: ____________

Signature:

________________________________ ________________
Signature of Principal Investigator Date

____
Principal Investigator Name (print or type)

This document contains confidential information, which should not be copied, referred to, released or published without written approval. Investigators are cautioned that the information in this protocol may be subject to change and revision.

CONFIDENTIAL
